# Synthesis of 4-Aminopyrazol-5-ols as Edaravone Analogs and Their Antioxidant Activity

**DOI:** 10.3390/molecules27227722

**Published:** 2022-11-09

**Authors:** Yanina V. Burgart, Galina F. Makhaeva, Olga P. Krasnykh, Sophia S. Borisevich, Natalia A. Agafonova, Nadezhda V. Kovaleva, Natalia P. Boltneva, Elena V. Rudakova, Evgeny V. Shchegolkov, Galina A. Triandafilova, Denis A. Gazizov, Olga G. Serebryakova, Maria V. Ulitko, Sergey L. Khursan, Victor I. Saloutin, Rudy J. Richardson

**Affiliations:** 1 Postovsky Institute of Organic Synthesis of the Ural Branch of the Russian Academy of Science, S. Kovalevskoi St., 22, Ekaterinburg 620108, Russia; 2 Institute of Physiologically Active Compounds at Federal Research Center of Problems of Chemical Physics and Medicinal Chemistry, Russian Academy of Sciences (IPAC RAS), Severny proezd 1, Chernogolovka 142432, Russia; 3 Scientific and Educational Center for Applied Chemical-Biological Research, Perm National Research Polytechnic University, Komsomolsky Av., 29, Perm 614990, Russia; 4 Ufa Institute of Chemistry of Russian Academy of Science, Octyabrya Av., 71, Ufa 450078, Russia; 5 Institute of Natural Sciences and Mathematics of the Ural Federal University Named after the First President of Russia B. N. Yeltsin, Lenina Av., 51, Ekaterinburg 620083, Russia; 6 Department of Environmental Health Sciences, University of Michigan, Ann Arbor, MI 48109, USA; 7 Department of Neurology, University of Michigan, Ann Arbor, MI 48109, USA; 8 Center of Computational Medicine and Bioinformatics, University of Michigan, Ann Arbor, MI 48109, USA; 9 Michigan Institute for Computational Discovery and Engineering, University of Michigan, Ann Arbor, MI 48109, USA

**Keywords:** Edaravone, 4-aminopyrazol-5-ols, 4-hydroxyiminopyrazol-5-ones, antioxidants, antiradical and ferric reducing activity, quantum-chemical calculations

## Abstract

One of the powerful antioxidants used clinically is Edaravone (EDA). We synthesized a series of new EDA analogs, 4-aminopyrazol-5-ol hydrochlorides, including polyfluoroalkyl derivatives, via the reduction of 4-hydroxyiminopyrazol-5-ones. The primary antioxidant activity of the compounds in comparison with EDA was investigated in vitro using ABTS, FRAP, and ORAC tests. In all tests, 4-Amino-3-pyrazol-5-ols were effective. The lead compound, 4-amino-3-methyl-1-phenylpyrazol-5-ol hydrochloride (APH), showed the following activities: ABTS, 0.93 TEAC; FRAP, 0.98 TE; and ORAC, 4.39 TE. APH and its NH-analog were not cytotoxic against cultured normal human fibroblasts even at 100 μM, in contrast to EDA. According to QM calculations, 4-aminopyrazolols were characterized by lower gaps, IP, and η compared to 4-hydroxyiminopyrazol-5-ones, consistent with their higher antioxidant activities in ABTS and FRAP tests, realized by the SET mechanism. The radical-scavenging action evaluated in the ORAC test occurred by the HAT mechanism through OH bond breaking in all compounds, directly dependent on the dissociation energy of the OH bond. All the studied compounds demonstrated the absence of anticholinesterase activity and moderate inhibition of CES by some 4-aminopyrazolols. Thus, the lead compound APH was found to be a good antioxidant with the potential to be developed as a novel therapeutic drug candidate in the treatment of diseases associated with oxidative stress.

## 1. Introduction

Oxidative stress is a crucial factor in the development of serious pathological conditions including cancer, aging, atherosclerosis, rheumatoid arthritis, cardiovascular and autoimmune diseases, and neurodegenerative disorders. It is characterized by an imbalance between the formation and neutralization of free radicals arising from the dysfunction of endogenous antioxidant protection systems. This redox abnormality leads to the critical accumulation of reactive oxygen and nitrogen species (ROS and RNS, respectively), such as peroxides and free radicals that damage cellular proteins, lipids, and nucleic acids. Antioxidants and radical scavengers play vital defensive roles by modulating the concentrations of ROS and RNS. Consequently, they can reduce the occurrence of disorders associated with oxidative stress [1,2].

The pyrazole core is considered to be a unique pharmacophore for the design of promising antioxidants [3,4,5]. One of the powerful antioxidants and neuroprotective drugs in clinical practice is 3-methyl-1-phenyl-2-pyrazolin-5-one (Edaravone, EDA, Radicut, Radicava). This prominent drug was originally used to treat brain infarction (stroke) [6,7,8] and was subsequently approved for the treatment of amyotrophic lateral sclerosis [9,10,11]. In addition, EDA can be used to treat Alzheimer’s disease, glaucoma, pulmonary diseases, cardiovascular dysfunctions, and medical chronic kidney damage, and to reduce specific side effects in the treatment of cancer [12,13,14]. Broadly speaking, EDA can be used to treat a range of diseases involving oxidative stress, and it has no severe adverse effects [12,15,16].

EDA has been found to be active in quenching free radicals, because the phenolic hydroxyl (exhibiting a propensity for scavenging free radicals) is generated from the tautomerization of its C=O group [17,18,19,20,21]. EDA effectively binds hydroxyl radicals by a consistent mechanism for electron–proton transfer [22,23] enabling it to react with alkoxyl radicals, superoxide anions, peroxyl radicals of lipids, peroxynitrite, and nitrogen(II) oxide [18,20,24]. EDA’s scavenging activity against multiple free radical species is as robust as other known potent antioxidants such as uric acid, glutathione, and Trolox [19].

Among analogs of EDA, 3-methyl-1-(pyridin-2-yl)-5-pyrazolone had the highest ability to scavenge radicals due to the increase in its active anion form stabilized by an intramolecular hydrogen bond in [25]. EDA derivatives bearing the NO-donor group in an aryl substituent showed different degrees of balance between antioxidant and vasodilating activity in vitro in [26,27]. Analogs having thiazolyl residues also demonstrated pronounced antioxidant properties in the ABTS test in [28]. Among 4-aryl- and 4-carboxyamide-MeO-derivatives of EDA, compounds with antioxidant properties in vitro were found in [29]. Furthermore, in another study, a series of DL-3-*n*-butylphthalide-EDA hybrids were synthesized as novel dual inhibitors of β-amyloid aggregation and monoamine oxidases for potential application as Alzheimer’s disease therapeutics, and these conjugates also possessed high antioxidant activity [30]. 

Thus, the EDA pyrazolone structure can be considered as a promising scaffold for the design of new effective antioxidants. This encouraged us to create new 4-amino-substituted EDA analogs and study their antioxidant properties. We believe that pyrazoles combining amino and hydroxyl groups in their structures should be promising as antioxidants.

For the synthesis of the 4-amino-5-alkoxy-3-methyl-1-phenylpyrazole hydrochloride, the use of reduction of the 4-nitro group in 5-RO-pyrazoles under the action of SnCl_2_ in concentrated HCl was described in [31]. Furthermore, 1-(sulfonated phenyl)-4-amino-5-pyrazolones were obtained through the reduction of hydroxyimino derivatives by zinc in a mixture of hydrochloric and acetic acids in [32] and by catalytic hydrogenation under the action of Pd/C in [33]. For the synthesis of N-glycoconjugated 4-amino-3-methyl-5-pyrazolones, the reaction was carried out with zinc in methanol in the presence of NH_4_Cl in [34]. In addition, a method was proposed for the synthesis of 3-substituted 4-aminopyrazol-5-ol hydrochlorides via cyclization of 2-amino-3-oxo esters with hydrazine in [35]. However, it should be noted that although the 4-amino derivative of EDA and its NH-unsubstituted analog were obtained in this way, there was no information on their structures.

Data on polyfluoroalkyl-containing 4-aminopyrazolols are limited to two examples describing the synthesis of 4-alkylamino-3-trifluoromethylpyrazol-5-ols via the recyclization of the relatively inaccessible compound 4-trifluoroacetyl-l,3-oxazolium-5-olate [36] or 5-trifluoromethyl-4-ethoxycarbonyl-1,3-oxazole [35] under the action of hydrazines. However, fluorinated derivatives, including pyrazoles, are undoubtedly promising as pharmacological agents due to the presence of fluorine atoms that change the physical, chemical, and biological properties of organic molecules [37,38,39,40,41,42,43,44]. For example, incorporation of fluorine atoms into drugs can increase their metabolic stability and lipophilicity, thereby facilitating penetration of the molecules through biological membranes [45]. 

Herein, we focused on the synthesis of 4-amino-substituted EDA derivatives, including polyfluoroalkyl-containing analogs, and the study of their primary antioxidant activity in comparison with EDA, CF_3_-EDA, and Trolox using ABTS, FRAP, and ORAC tests. To explain the factors influencing antioxidant activity, we performed quantum-chemical calculations. Taking into account the literature data on the ability of pyrazole derivatives to inhibit acetylcholinesterase [46,47,48,49,50] and human carboxylesterase-1 [51], we also investigated the esterase profiles of the new compounds, i.e., their inhibitory activity against acetylcholinesterase (AChE), butyrylcholinesterase (BChE), and carboxylesterase (CES). In addition, the effect of the compounds on cell viability was evaluated in normal human dermal fibroblast cultures.

## 2. Results and Discussion

### 2.1. Chemistry

For the synthesis of polyfluoroalkyl-containing 4-aminopyrazol-5-ols, we used the reduction of 4-hydroxyiminopyrazol-5-ones **1a**–**j**, for which we have recently proposed convenient synthesis methods [52].

**Scheme 1:**
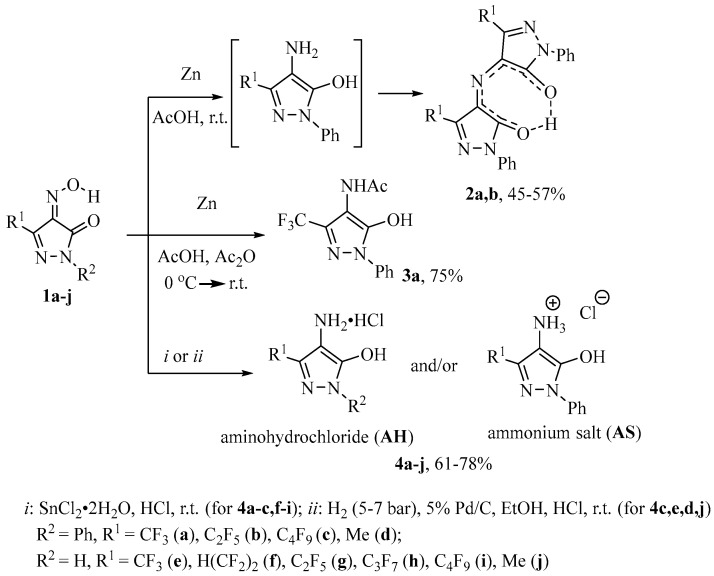
Reduction of 4-hydroxyiminopyrazolones **1a**–**j**.

First, we tried to carry out reduction of 4-hydroxyiminopyrazolones **1a**,**b** with zinc in acetic acid, according to our successful method for the synthesis of 4-amino-3-trifluoromethyl-5-alkyl[(het)aryl]pyrazoles from 4-nitrosopyrazoles [53]. However, this method did not allow us to obtain the expected 4-aminopyrazol-5-ols, because bis-[5-hydroxy-1-phenyl-3-(polyfluoroalkyl)-1*H*-pyrazol-4-yl]imines **2a**,**b** were isolated from these reactions as a result of crosslinking of two pyrazolone molecules by the amino group (Scheme 1). Similar transformations have been described for non-fluorinated analogs [35,54]. Although the mechanism of formation of these compounds is unclear, it can be assumed that the free amines are unstable and convert to bis-pyrazoles **2a**,**b** due to oxidation under the action of oxygen in the air [55].

**Figure 1:**
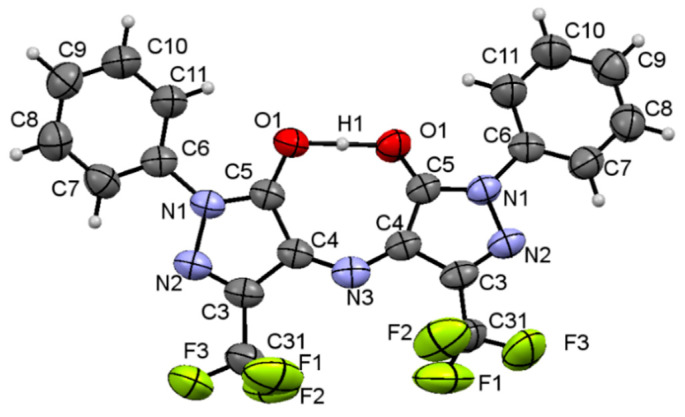
The structure of compound **2a** according to XRD.

The bis-pyrazole structure of compound **2a** was confirmed by XRD (Figure 1). In its crystal structure, the symmetry axis passing through the exocyclic nitrogen atom N3 and the proton H1 of the hydroxyl group can be marked. The two pyrazole molecules are mirror images of each other and generate a “crab”-like form. The eight-membered pseudocycle O1C5C4N3C4C5O1H1 is practically flat, with the O1 oxygen atom deviating from the plane by 0.353 Å. The compound is characterized by delocalization of double bonds (the lengths of C4N3 and N4C3 are equal to 1.312 Å), and the proton H1 of the hydroxyl group is delocalized between the two O1 oxygen atoms, with the distance O1H1 equal to H1O1 (1.208 Å).

When adding acetic anhydride to the reducing mixture Zn/AcOH, reduction of the hydroxyimine group of pyrazolone **1a** was accompanied by acylation of the resulting amino function to yield 4-acylaminopyrazol-5-ol **3a** (Scheme 1).

4-Aminopyrazol-5-ols **4a**–**c**,**f**–**i**, as hydrochlorides, were synthesized by reducing 4-hydroxyiminopyrazolones **1a**–**c**,**f**–**i** under the action of tin(II) chloride in concentrated HCl (Scheme 1). However, the use of this synthetic protocol was not effective for the preparation of NH-unsubstituted 4-amino-3-CF_3_-pyrazol-5-ol **4e**, and catalytic hydrogenation in EtOH-HCl in the presence of Pd/C was used for its synthesis. The application of such a system facilitates the isolation of amine salts, as shown in the preparation of compound **4c**.

The same method was used to prepare the 4-amino derivative of EDA **4d** and its NH-unsubstituted analog **4j** from the corresponding 4-hydroxyiminopyrazol-5-ones **1d**,**j** by catalytic reduction (Scheme 1). Compounds **4d**,**j** were characterized by IR along with ^1^H and ^13^C NMR spectroscopy, because spectral data for these compounds were lacking [35].

Attempts to carry out catalytic hydrogenation of compounds **1a**,**b**,**d** under acid-free conditions led to bis-pyrazoles **2a**,**b** or to the resinification of the reaction mass. It can be concluded that 4-aminopyrazol-5-ol as a free base is unstable.

The structure of 4-aminopyrazoles **4a**–**j** was confirmed by IR and NMR spectroscopy. The NMR spectra of these compounds recorded in DMSO-*d*_6_ are characterized by revealing one hydroxyl form that was confirmed by the presence in the ^13^C NMR spectra of compounds **4** of carbon atom signals of =C-OH at δ 147–154 ppm. According to NMR spectroscopy data, aminopyrazole hydrochlorides **4a**,**b** (form **AH**) standing in DMSO solutions were able to convert to ammonium salts (form **AS**) (Scheme 1). Thus, in the ^1^H NMR spectrum of hydrochlorides **4a**,**b**, the proton signals of the amino group were broad low-field signals or absent due to deuterium exchange with solvent water, while the spectra after standing of a sample for 6–12 h contain triplet signals of protons of NH_3_^+^ groups of ammonium salts. In addition, the content of the **AS** form increases with elongation of the polyfluoroalkyl substituent. The **AH**:**AS** ratio was 77:23 (for **4a**) and 40:60 (for **4b**). The ^13^C NMR spectra of compounds **4a**,**b** also contained two sets of signals characterized by the multiplet broadening of the signals of carbon atoms for the ammonium salt and by singlet signals of the same carbons for the hydrochloride. However, compound **4c** bearing a nonafluorobutyl residue existed in DMSO solution only as **AS**, while the methyl-containing analog **4d** occurred as **AH**. All N-unsubstituted aminopyrazoles **4e**–**j** were revealed to adopt only the **AH** form.

### 2.2. Evaluation of Antioxidant Potential of Pyrazoles ***1*** and ***4***

For studying the primary antioxidant activity of compounds **1** and **4**, three different methods were selected: (1) the FRAP test, which exclusively evaluates the single electron transfer (SET) mechanism; (2) the ORAC-FL test, evaluating exclusively the hydrogen atom transfer (HAT) mechanism; and (3) the ABTS test, which reflects either HAT or SET mechanisms as well as their combination. For all methods, Trolox was used as the reference antioxidant: antioxidant activity of the compounds was presented relative to the activity of Trolox. Quercetin was used as a positive control. The data are presented in Table 1.

The ABTS assay is based on the direct quenching of the ABTS cation radical (ABTS^•+^) by antioxidants. The assay is carried out by spectrophotometric determination of a decrease in absorbance of a stable dark green ABTS^•+^ solution after its interaction with an antioxidant compound [56]. The measurements were performed as previously described in detail [57]. The results are expressed as TEAC values (Trolox equivalent antioxidant capacity) and IC_50_ values (compound concentration required for 50% reduction of the ABTS radical).

The FRAP (ferric reducing antioxidant power) assay measures the ability of antioxidants to reduce the ferric 2,4,6-tripyridyl-s-triazine complex [Fe(TPTZ)_2_]^3+^ to the intensely blue ferrous complex [Fe(TPTZ)_2_]^2+^ in an acidic medium [58]. The measurements were performed as previously described in detail [59]. The results are expressed as TE (Trolox equivalents).

In the oxygen radical absorbance capacity (ORAC-FL) assay, 2,2′-azobis-(2-methylpropionamidine) dihydrochloride (AAPH) is used as a free peroxyl radical generator, and fluorescein (FL) is used as a fluorescent probe [60]. The method is based on measuring the decrease in the intensity of fluorescence with time, which characterizes the degree of decay of the fluorescent probe under the influence of peroxyl radicals. In the presence of antioxidants, the degree of decay of the fluorescent probe decreases and, accordingly, the fluorescence intensity increases. The results are expressed as TE (Trolox equivalents).

#### 2.2.1. ABTS Assay

As shown in Table 1, fluorine-containing NH-unsubstituted 4-hydroxyiminopyrazol-5-ones **1e**–**i** demonstrated moderate radical-binding activity in the ABTS test, which was practically independent of the R^F^ substituent structure (TEAC = 0.13–0.23). The introduction of a phenyl substituent into position 1 of the pyrazole ring resulted in a sharp decrease in the antiradical activity of compounds **1a**–**c** regardless of the R^F^ structure: **1e** vs. **1a**, (R^F^ = CF_3_); **1g** vs. **1b**, **1i** vs. **1c** (R^F^ = C_4_F_9_).

The replacement of the fluorine-containing radical with the methyl group at position 3 promoted an increase in the radical-binding activity of the phenyl-containing derivative (**1d** vs. **1a**) and did not affect the activity of the NH-unsubstituted derivative (**1j** vs. **1e**). The latter compounds exhibited an equally moderate ABTS^•+^-binding activity.

In contrast to 4-hydroxyiminopyrazol-5-ones **1**, aminopyrazoles **4** showed high radical-binding activity in the ABTS test at the level of Trolox and EDA. The ABTS-binding activity of 4-unsubstituted 3-trifluoromethyl-1-phenylpyrazol-5-ol (CF_3_-EDA) was found to be equal to that of EDA.

In addition, the introduction of a Ph substituent to position 1 of the pyrazole ring in 4-amino-3-R^F^-pyrazolols **4** retained their high antioxidant activity.

The elongation of the polyfluoroalkyl radical in NH-unsubstituted 4-aminopyrazoles **4** resulted in a gradual decrease in the ABTS-binding activity in the following order: **4e** > **4g**~**4h** > **4i**. The activity was halved in the transition from CF_3_-pyrazole **4e** to the C_4_F_9_ analog **4i**.

Similarly to 4-hydroxyiminopyrazolones **1e**,**j**, the replacement of the CF_3_ group by a Me residue at position 3 fostered an increase in the ABTS^•+^-binding activity of aminopyrazolol **4d** in the presence of a Ph substituent at position 1 (**4d** vs. **4a**) and retention of high activity in the case of NH-unsubstituted derivatives (**4j** vs. **4e**).

Thus, aminopyrazoles **4c**,**d**,**e**,**j** exhibited the greatest activity in the ABTS test and had TEAC values at the level of Trolox, EDA, and CF_3_-EDA. It is also noteworthy that our ABTS test data on the antioxidant activity of EDA matched the values reported in the literature [61,62].

#### 2.2.2. FRAP Assay

Compounds **1** and **4** demonstrated structure–activity relationships in the FRAP assay close to the results from the ABTS test, although in some cases they were less pronounced.

Thus, 4-hydroxyiminopyrazolones **1** were either inactive or exhibited very low activity compared to the standard antioxidant Trolox. The introduction of a phenyl substituent into position N1 reduced ferric reducing activity and, correspondingly, antioxidant activity: **1a** vs. **1e** (R = CF_3_), **1b** vs. **1g** (R = C_2_F_5_), **1d** vs. **1j** (R = Me).

As in the ABTS test, 4-aminopyrazolols **4** were significantly more active as antioxidants compared to 4-hydroxyiminopyrazolones **1**. As shown in Table 1, most of the compounds of this group showed high activity at the level of the standard antioxidant Trolox.

The introduction of a Ph-substituent at position 1 of the pyrazole ring of compounds **4** retained their high antioxidant activity compared to unsubstituted ones. Activity increased for compounds with R^F^ = C_4_F_9_ (**4c** was more active than **4i**).

Similar to the results of the ABTS test, the replacement of the CF_3_ group with CH_3_ at position 3 promoted an increase in ferric reducing activity in the presence of a Ph substituent at position 1 (**4a** vs. **4d**) and maintained high activity in the case of the NH-unsubstituted derivatives (**4e** vs. **4j**).

Interestingly, according to literature data [25], CF_3_-EDA was 3 times less active compared to EDA in the OH radical binding test (hydroxyl radical scavenging activity). Meanwhile, in the ABTS test, the activity of CF_3_-EDA was at the level of EDA and Trolox, and in the FRAP test, its ferric reducing activity was only 20% lower than that of EDA (Table 1).

Thus, Ph-substituted (**4a**,**c**,**d**) and NH-unsubstituted (**4e**,**g**,**h**,**j**) 4-aminopyrazoles showed high activity in the FRAP test. In addition, compounds **4c**,**d**,**e**,**j** showed significant activity in the ABTS test. Moreover, the ferric reducing activity of compounds **4a**,**c**,**d**,**e**,**g**,**h**,**j** was at the level of Trolox and exceeded the activity of EDA or CF_3_-EDA.

#### 2.2.3. ORAC Assay

The ORAC assay utilizes a biologically relevant radical source, 2,2′-azobis(2-amidinopropane) dihydrochloride (AAPH)-derived peroxyl radicals. Thus, it is a more physiologically relevant assay than ABTS and FRAP.

As shown in Table 1, overall, the results of the ABTS and FRAP tests agree with the ORAC-FL data; i.e., all compounds that were active in the first two tests showed a pronounced antioxidant effect in the ORAC test. However, there were also differences. In particular, the methyl analog **1d** showed activity at the Trolox level (TE = 1.07) in the ORAC test. In addition, NH-unsubstituted 4-hydroxyiminopyrazol-5-ones **1e**,**h**,**i**,**j**, which were inactive or low-active in the FRAP test or exhibited moderate activity in the ABTS test, showed a pronounced radical binding effect in the ORAC test. This effect was higher for compounds with a longer polyfluoroalkyl substituent (**1e** < **1h** = **1i**).

Structure–activity analysis for results in the ORAC test showed that all compounds in the series of 4-amino derivatives **4** exhibited high antioxidant activity, in contrast to 4-hydroxyimino derivatives **1**. The introduction of an N-phenyl substituent into position 1 significantly enhanced the effect (**4a** vs. **4e**, **4b** vs. **4g**, **4c** vs. **4i**, **4d** vs. **4j**).

In each of the subgroups **1a**–**d**, **1e**–**j**, and **4a**–**d**, **4e**–**j**, compounds **1d**,**j** and **4d** containing a methyl group instead of a polyfluoroalkyl group exhibited higher activity. The most active among the aminopyrazolols **4** were compounds **4a** (R^1^ = CF_3_, R^2^ = Ph, 3.49 TE) and **4d** (R^1^ = Me, R^2^ = Ph, 4.39 TE).

In particular, the lead compound **4d** was more active than EDA (3.71 TE) and CF_3_-EDA (3.89 TE).

IC_50_ values were determined for compounds **4a**,**d**,**e**, EDA, CF_3_-EDA, and Trolox (Table 1). The obtained IC_50_ values were 3–5 times lower than the IC_50_ of Trolox, again confirming the high antioxidant activity of the tested pyrazole derivatives.

Thus, the results of the ABTS and FRAP tests show that 4-hydroxyiminopyrazolones **1** had moderate or very weak activity (HN-derivatives **1e**–**j** were more active than Ph-N-analogs **1a**–**d**), while 4-aminopyrazolols **4** demonstrated a pronounced antioxidant effect comparable to Trolox (additionally, HN- and PhN-derivatives **4a**–**d** and **4e**–**j** had close activity). The most active compounds in both of these tests were the 4-aminopyrazolols **4d**,**e**,**j**. In contrast, HN-unsubstituted 4-hydroxyiminopyrazolones **1e**–**j** and all 4-aminopyrazolols **4a**–**j** were active in the ORAC test. The increase in activity of 4-aminopyrazolol **4** was promoted by the introduction of a phenyl substituent and the replacement of a fluoroalkyl substituent with a methyl group. The lead compound **4d** was more active in all tests than EDA and CF_3_-EDA.

### 2.3. Assessment of Esterase Profile of Pyrazoles ***1a**–**j*** and ***4a**–**j***

Given the available literature data on the ability of pyrazole derivatives to inhibit acetylcholinesterase (AChE) [46,47,48,49,50] and human carboxylesterase-1 (CES) [51], we performed an investigation of the esterase profile for new compounds **1a**–**j** and **4a**–**j**, including an evaluation of their inhibitory effect on the standard enzymes set: human erythrocyte AChE, equine serum butyrylcholinesterase (BChE), and porcine liver CES (Table 2). The applicability of this set of enzymes was shown by us earlier [63,64,65,66].

It was found that 4-hydroxyminopyrazolones **1a**–**j** did not substantially inhibit any of the esterases, while aminopyrazolols **4a**,**c**,**d**,**h**,**i** showed moderate anticarboxylesterase activity with IC_50_ values in the range 10–98 μM, with a maximum activity of 10 and 22 μM for compounds **4c** and **4i**, respectively, which had a nonafluorobutyl substituent. These data indicate the absence of anticholinesterase side effects, with the potential therapeutic application of these compounds and some probability of undesirable drug interactions from the use of aminopyrazolols **4** at high doses, owing to the inhibition of CES, which participates in the primary metabolism of numerous drugs with ester groups.

### 2.4. Quantum-Chemical Calculations of Antioxidant Activity

Herein, we used three analysis methods based on different reaction mechanisms, namely ORAC (HAT), FRAP (SET), and ABTS (SET or/and HAT), for an experimental assessment of antioxidant activity (AOA) of analogs of EDA **1a**–**j**, **4a**–**j** [67,68]. We noted that the values obtained with the ABTS and FRAP assays were significantly correlated (ρ = 0.702, *p* = 0.003), which was not the case for ABTS and ORAC (ρ = 0.391, *p* = 0.134) or FRAP and ORAC (ρ = 0.006, *p* = 0.985) (Spearman non-parametric correlation). These results may indicate the same SET mechanism of the antioxidant action of compounds **1a-j**, **4a**–**j** in FRAP and ABTS tests, whereas another mechanism (HAT) is realized in ORAC assay.

Computational strategies are widely used to investigate AOA [69,70,71,72,73]. To identify parameters responsible for the antioxidant effect of the studied compounds **1a**–**j** and **4a**–**j**, we performed theoretical calculations of their electronic characteristics using the GAUSSIAN 09 program [74]. The most popular hybrid functional (B3LYP) [75] combined with the basis sets cc-pVDZ [76] was used throughout the work for geometry optimization and frequency calculations. Moreover, geometrical parameters of **4a**–**j** were optimized using the Polarizable Continuum Model (PCM) [77].

The scavenging activity of the aromatic compounds was shown to be directly proportional to the negative of the gaps computed as the energy difference between the HOMO and LUMO [78]; thus, we calculated such values for the synthesized analogs EDA **1a**–**j** and **4a**–**j** (Table 3).

As is known, the ionization potential *IP* is directly related to SET reactions; such an approach is valuable and provides primary physicochemical insight into the mechanism of action in tests where the SET mechanism is operational. Therefore, we analyzed the relationships of the AOA of compounds **1a**–**j**, **4a**–**j** defined in the ABTS and FRAP tests with their structure, using indices calculated from the vertical values of *IP* and *EA*. The use of only vertical values is due to the fact that the loss or capture of an electron by a molecule leads to an essential change in the structure and, consequently, the composition and energies of molecular orbitals. The vertical *IP* and *EA* quantities of all studied compounds **1a**–**j**, **4a**–**j** were collected in Table 3.

According to the experimental results, compounds **1** and **4** differed significantly in activity (Table 1), and the *IP* and gap calculation data confirmed this. For correct interpretation of the calculated quantum-chemical results, it is reasonable to consider the properties of compounds **1** and **4** within the following subgroups: polyfluoroalkyl (**1a**–**c** and **4a**–**c**) and methyl (**1d** and **4d**) derivatives with a phenyl substituent and polyfluoroalkyl (**1e**–**f** and **4e**–**i**) and methyl (**1j** and **4j**) derivatives with an NH fragment.

According to the data in Table 3, 4-aminopyrazolols **4a**–**j** have lower *IP*, gap, and η values compared to 4-hydroxyiminopyrazolones **1a**–**j**. This suggests that compounds **4a**–**j** should be more active than pyrazolones **1a**–**j**, and this was observed experimentally.

Compounds **1a**–**d** containing the N-Ph moiety have lower *IP*, gap, and η values compared to NH analogs **1e**–**j**. However, according to experimental data (Table 1), 4-hydroxyiminopyrazolones **1a**–**d** having a N-Ph group exhibited less antioxidant activity compared to HN-unsubstituted derivatives **1e**–**j**. Polyfluoroalkyl-containing compounds **1a**–**c** and **1e**–**i** have slightly higher *IP* values than methyl derivatives **1d** and **1j**. In this case, their gap and η values were nearly equal. According to the experiment, the fluorine-containing derivatives were somewhat less active than their methyl analogs.

4-Aminopyrazolol hydrochlorides **4** exhibited a pronounced antioxidant activity compared to 4-hydroxyiminopyrazolones **1** (Table 1), and this is consistent with lower *IP*, gap, and η values of compounds **4a**–**j** compared to 4-hydroxyiminopyrazolones **1a**–**j**. In the series of compounds **4**, the property-changing tendency was similar to compounds **1** as a whole: compounds **4a**–**d** containing the N-phenyl moiety had lower *IP*, gap, and η values comparing to compounds **4e**–**j**. However, the activities of both sets **4a**–**d** and **4e**–**j** in the antioxidant experiment were approximately the same. Methyl derivatives **4d** and **4j** had a more pronounced antioxidant activity, and this was consistent with their lower *IP*, gap, and η values than those of fluorinated analogs **4a** and **4e**.

Next, we analyzed the dependencies of AOA (TEAC) on the calculated parameters of *IP*, gap, and η (eV) for compounds **1** and **4** according to the work of Horton et al. [78] for aromatic compounds having hydroxyl and amino groups.

Considering the possible dependencies of the experimentally found AOA values in the ABTS test (as more complete) on the calculated *IP*, gap, and η values for 4-hydroxyiminopyrazolones **1a**–**j**, we found that there was no direct dependence between the calculated *IP* and gap parameters with experimental AOA values.

**Figure 2:**
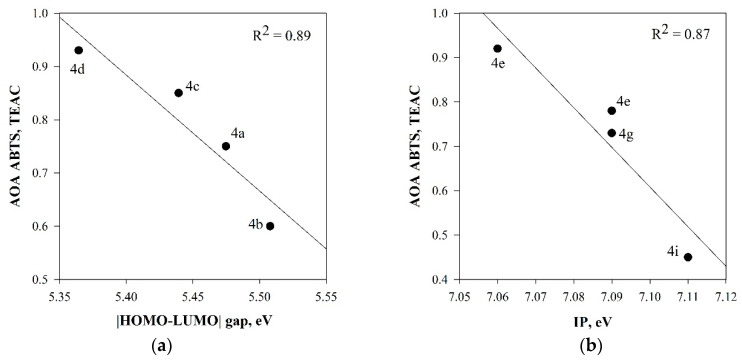
Antioxidant activity (AOA, ABTS) vs. gap functions for 4-aminopyrazolols **4a–d** (**a**) and (AOA, ABTS) vs. *IP* functions for 4-aminopyrazolols **4e**, **4h–i** (**b**).

At the same time, in the series of 4-aminopyrazolols **4** for all PhN-substituted compounds **4a**–**d**, there were good negative dependences between their AOA values and gap functions (Figure 2a). It was not possible to identify acceptable relationships for HN-unsubstituted analogs **4e**–**j**, because derivatives **4j** and **4f** containing Me- and H(CF_2_)_2_-substituents were expected to fall out of this series. For aminopyrazoles **4e**,**g**–**i** containing perfluoroalkyl substituent F(CF_2_)_n_, there was a dependence of their AOA on the *IP* function, with a favorable coefficient of determination (Figure 2b).

According to the lower gap values obtained for compounds **1a**–**d** and partially for aminopyrazoles **4a**–**d** with phenyl substituents, a higher AOA could be expected compared to HN-heterocycles **1e**–**j** and **4e**–**j**. However, this effect was observed for only one pair of 4-aminopyrazolols, **4c** and **4i**. Differences in theoretical and experimental data can be explained by the structure of these polyfunctional compounds and various transformations of molecules with a PhN substituent and HN group during antioxidant action. In addition, it is possible that the ABTS-binding effect of compounds **1** and **4** is realized not only by the SET mechanism but also partially by the HAT mechanism. This is supported by an incomplete correlation of the values from the ABTS test with the antioxidant action values from the FRAP test, where only the SET mechanism is implemented (Table 1).

In order to interpret the radical scavenging activity of antioxidants in reactions by the HAT mechanism, a common characteristic is known to be the bond-dissociation energy (BDE) [79,80]. Thus, using quantum-chemical calculations (B3LYP/cc-pVDZ method) in the approximation of the gas phase, we estimated the BDE (kJ/mol) values in the formation of possible radicals from compounds **1** and **4**.

**Scheme 2:**
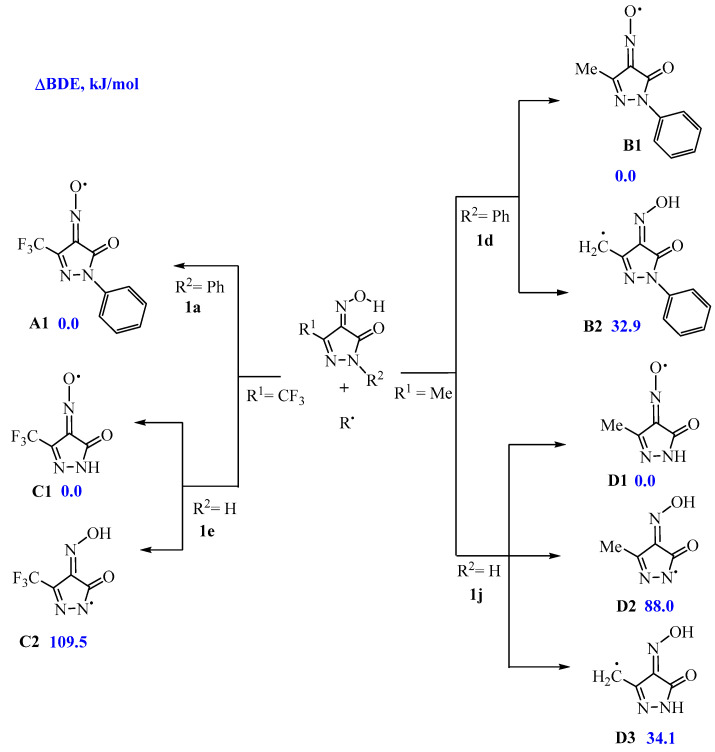
The formation of possible radicals in the reactions of 2-hydroxyiminopyrazolones **1a**,**d**,**e**,**j** with values of the difference in bond dissociation energies (ΔBDE, kJ/mol), which were evaluated in the approximation of the gas phase (B3LYP/cc-pVDZ method).

For 2-hydroxyiminopyrazolones **1a**–**j**, various radicals (examples for compounds **1a**,**d**,**e**,**j** are shown in Scheme 2) are likely to be formed. It is possible to form only one radical, **A1**, from 2-hydroxyiminopyrazolone **1a**, while for each of compounds **1d**,**e**, two different radicals, **B1**, **B2** and **C1**, **C2**, respectively, can be formed. In the case of NH-unsubstituted methylpyrazolone **1j**, four **D1**–**D4** radicals can be generated. According to the calculated data for all compounds **1a**,**d**,**e**,**j**, the formation of O-radicals **A1**, **B1**, **C1**, **D1** through breaking of the -O-H bond of the hydroxyimine substituent is the most likely. The difference in the BDE values in the formation of O-radical and the other variants is presented in Scheme 2.

**Scheme 3:**
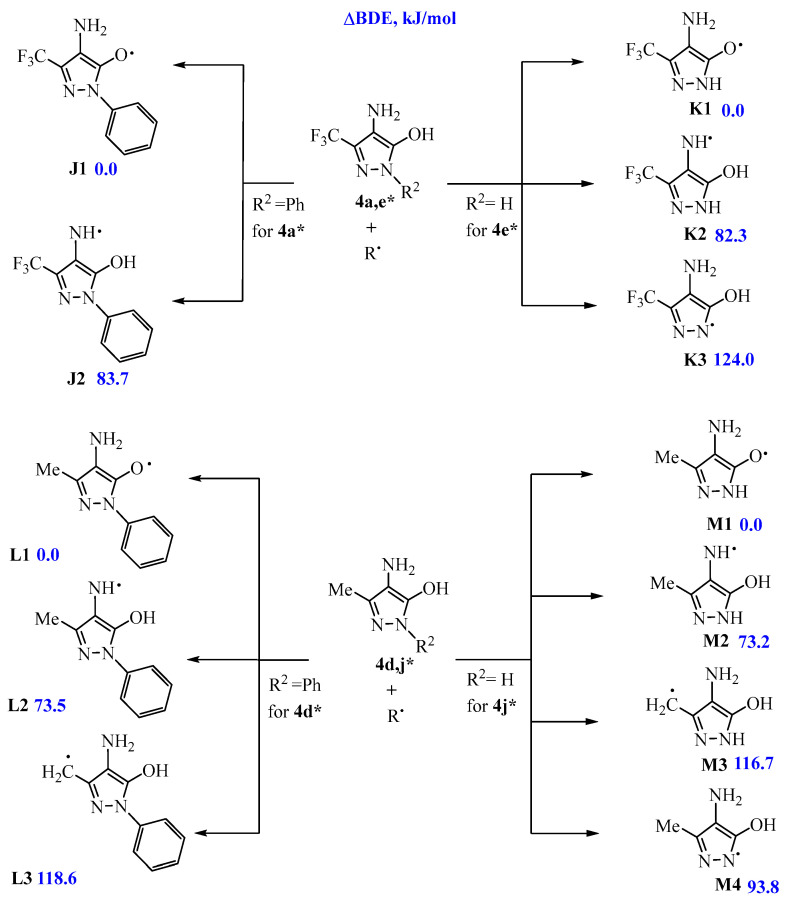
The formation of possible radicals in the reactions of 4-aminopyrazolols **4a**,**d**,**e**,**j** * (* conversions are given for 4-aminopyrazolols), with values of the difference in bond dissociation energies (ΔBDE, kJ/mol), which were evaluated in the approximation of the gas phase (B3LYP/cc-pVDZ method).

For 4-aminopyrazolol hydrochlorides **4a**–**j**, similar transformations can also be assumed, but taking into account the reactions of the hydroxyl and the amino groups, this leads to more complex and varied conversions. Thus, CF_3_-containing 4-aminopyrazolols **4a**,**e** can theoretically form two **J1**, **J2** (for **4a**) or three **K1**–**K3** (for **4e**) radicals (Scheme 3). Non-fluorinated analogs **4d**,**j** can undergo reactions involving a Me group, thereby forming three **L1**–**L3** (for **4d**) or four **M1**–**M4** (for **4j**) radicals. Evaluating the difference between the calculated BDE values, the most likely process for compounds **4a**,**d**,**e**,**j** was found to generate radicals **J1**, **K1**, **L1**, and **M1**, forming via -O-H bond breaking (the ΔBDE values are indicated in blue in Scheme 3) despite the position of this substituent in these pyrazoles changes. This process is similar to the transformations of structures **1a**,**d**,**e**,**j**.

**Figure 3:**
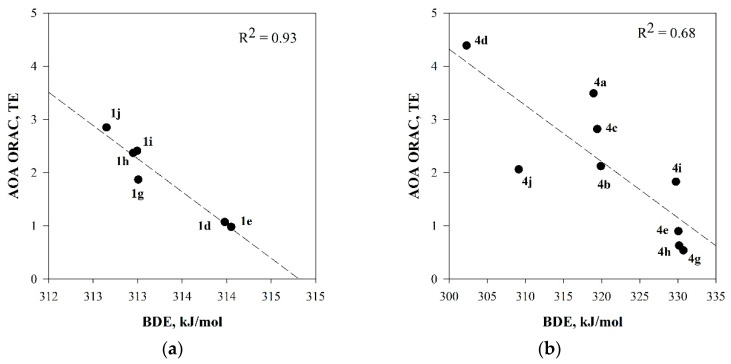
Antioxidant activity (AOA, ORAC) vs. BDE functions for 4-hydroxyimino-1-phenyl-pyrazolones **1e**–**j** (**a**) and antioxidant activity (AOA, ORAC) vs. BDE functions for polyfluoroalkyl 4-aminopyrazolols **4a**–**c**, **4e**–**i** (**b**).

Given that by using quantum-chemical calculations for pyrazoles **1d**,**j**, **4d**,**j**, it was shown that the formation of O-radicals is energetically most advantageous in all cases (Scheme 2 and Scheme 3), we calculated the BDE values for -OH bond breaking in compounds **1a**–**j** and **4a**–**j** (Table 3). This revealed a linear relationship with a highly favorable coefficient of determination between the AOA from the ORAC test and the BDE (OH) values for a series of active NH-unsubstituted 4-hydroxyiminopyrazolones **1e**–**j** (Figure 3a).

All tested aminopyrazolols **4a**–**e**,**g**–**j** were active in the ORAC test, and an acceptable linear relationship between AOA and BDE for polyfluoroalkyl derivatives of **4a**–**c**,**e**,**g**–**i**, was found (Figure 3b), regardless of the presence of a Ph substituent at the nitrogen atom in the molecule. It was impossible to find such a pattern for non-fluorinated analogs owing to the limited samples including only **4d**, **4j**.

According to the calculated BDE values (Table 3), HN-unsubstituted 4-hydroxyiminopyrazolones **1e**–**j** are more active than 4-aminopyrazolols **4e**–**j**, and this is consistent with the AOA values from the ORAC test. According to the BDE values, the PhN-containing 4-aminopyrazolols **4a**–**d** should be more active than the HN analogs **4e**–**j**, which also coincides with the experiment.

### 2.5. Cytotoxicity Studies

To investigate the effect of the lead compound **4d** as well as its NH analog **4j** in comparison with EDA on cell viability, cytotoxicity studies for these compounds were performed on normal human dermal fibroblast (NHDF) cultures using the MTT/formazan assay [81].

EDA appeared to have a dose-dependent effect on NHDF culture viability, the inhibitory capability of which increased with rising concentration (Table 4).

Meanwhile, at a concentration of 100 µM, neither of the compounds **4d**,**j** showed cytotoxicity against NHDF cell culture.

## 3. Materials and Methods

### 3.1. Chemistry

Acetic acid, chloroform, dimethylsulfoxide, ethanol, *n*-hexane, hydrochloric acid, and zinc (dust) were obtained from AO “VEKTON” (St. Petersburg, Russia). Pd/C (5 wt.%) was purchased from Alfa Aesar by Thermo Fisher Scientific (Kandel, Germany). The deuterosolvent DMSO-*d*_6_ was acquired from “SOLVEX” Limited Liability Company (Skolkovo Innovation Center, Moscow, Russia). Melting points were measured in open capillaries on a Stuart SMP30 melting point apparatus (Bibby Scientific Limited, Staffordshire, UK) and were uncorrected. The IR spectra were recorded on a Perkin Elmer Spectrum Two spectrophotometer (PerkinElmer, Waltham, MA, USA) using a frustrated total internal reflection accessory with a diamond crystal. The ^1^H and ^19^F NMR spectra were registered on a Bruker DRX-400 (400 or 376 MHz, respectively) or a Bruker Avance^III^ 500 (500 or 470 MHz, respectively) (Bruker, Karlsruhe, Germany). The ^13^C NMR spectra were recorded on a Bruker Avance^III^ 500 (125 MHz). The internal standard was SiMe_4_ (for ^1^H and ^13^C NMR spectra) and C_6_F_6_ (for ^19^F NMR spectra). The microanalyses (C, H, N) were carried out on a PerkinElmer PE 2400 series II elemental analyzer (PerkinElmer, Waltham, MA, USA). Chlorine was measured by mercurimetric titration. The column chromatography was performed on silica gel 60 (0.062–0.2 mm) (Macherey-Nagel GmbH & Co KG, Duren, Germany).

The initial 4-hydroxyiminopyrazol-5-ones **1a**–**h** were synthesized by the previously published method [52].

#### 3.1.1. Synthesis of Rubazonic Acids **2a**,**b** (General Procedure)

Pyrazolone **1a**,**b** (5 mmol) was dissolved in glacial acetic acid (5 mL), and zinc dust (0.98 g, 15 mmol) was added. The reaction mixture was stirred for 4 h at room temperature (r.t.) and left standing overnight. Then, water (20 mL) was added, and the precipitate was filtered off and dried.

*Bis[5-hydroxy-1-phenyl-3-(trifluoromethyl)-1H-pyrazol-4-yl]imine (***2a***)*. Yield 1.33 g (57%), red crystals, m.p. 188–189 °C. ^1^H NMR (500 MHz, CDCl_3_): δ 7.40–7.43, 7.50–7.53, 7.85–7.87 (all m, 10H, 2Ph); 17.16 (s, 1H, OH). ^13^C NMR (500 MHz, CDCl_3_): δ 119.23 (q, CF_3_, *J* 272.2 Hz); 121.29; 123.24; 128.33; 129.29; 136.42; 144.39 (q, C–CF_3_, *J* 37.4 Hz); 151.96. ^19^F NMR (500 MHz, CDCl_3_): δ 96.93 (s, CF_3_). Anal. calcd. for C_20_H_11_F_6_N_5_O_2_: C 51.40; H 2.37; N 14.99. C_20_H_11_F_6_N_5_O_2_. Found: C 51.54; H 2.32; N 14.94.

*Crystallographic data for compound***2a**. The X-ray studies were performed on an “Xcalibur 3 CCD” diffractometer with a graphite monochromator, ω scanning with 1° step, λ(MoKα) 0.71073 Å radiation, T 295(2) K. Empirical absorption correction was applied. Using Olex2 [82], the structure was solved with the ShelXS [83] structure solution program using Direct Methods and refined with the ShelXL [83] refinement package using Least Squares minimization. All non-hydrogen atoms were refined in the anisotropic approximation; H-atoms at the C-H bonds were refined in the “rider” model with dependent displacement parameters. Empirical absorption correction was carried out through spherical harmonics, implemented in SCALE3 ABSPACK scaling algorithm by the program “CrysAlisPro 1.171.36.32” (Rigaku Oxford Diffraction, 2013).

Suitable red single crystals of compound **2a** were obtained by slow crystallization from CHCl_3_. Main crystallographic data for **2a**: C_20_H_11_F_6_N_5_O_2_, *M* = 467.34, space group C2/c, monoclinic, *a* 19.559(2), *b* 20.5241(19), *c* 9.6419(16) Å; *β* 93.650(12)°; *V* = 3862.8(8) Å^3^; *Z* 8; *μ* 0.147 mm^−1^; 303 refinement parameters; 11207 reflections measured; 3921 unique (*R*_int_ 0.0664), which were used in all calculations. The final *wR*_2_ was 0.1299 (all data), and *R*_1_ was 0.0588 [I >= 2σ (I)]. CCDC 2183294 contains the supplementary crystallographic data for this compound.

*Bis[5-hydroxy-3-(pentafluoroethyl)-1-phenyl-1H-pyrazol-4-yl]imine (***2b***)*. Yield 1.28 g (45%), red crystals, m.p. 134–135 °C. ^1^H NMR (500 MHz, CDCl_3_): δ 7.40–7.43, 7.50–7.54, 7.86–7.88 (all m, 10H, 2Ph); 16.96 (s, 1H, OH). ^19^F NMR (CDCl_3_): δ 46.75 (br. s, 2F, CF_2_), 78.71 (br. s, 3F, CF_3_). Anal. calcd. for C_22_H_11_F_10_N_5_O_2_: C, 46.58; H, 1.95; N, 12.34. Found: C, 46.46; H, 2.04; N, 12.21.

*N-(5-Hydroxy-3-(trifluoromethyl)-1-phenyl-1H-pyrazol-4-yl)acetamide (***3a***).* Pyrazolone **1a** (0.51 g, 2 mmol) was dissolved in glacial acetic acid (5 mL), and zinc dust (0.25 g, 4 mmol) was added. The reaction mixture was stirred for 30 min at r.t. Then, acetic anhydride (1 mL) was slowly added at 0 °C, and the reaction was left standing overnight at r.t. Then, water (20 mL) was added, and the precipitate was filtered off. The residue was purified by column chromatography (eluent–CHCl_3_/EtOH-10:1). Yield 0.43 g (75%), orange powder, m.p. 97–98 °C. IR: *ν* 3629, 3373, 3272, 3066 (OH, NH); 1649 (C=O); 1596, 1497, 1480 (C=N, C=C); 1228–1129 (C-F) cm^−1^. ^1^H NMR (400 MHz, CDCl_3_) δ 2.26 (s, 3H, Me), 7.27 (br. s, 1H, NH), 7.32–7.33, 7.44–7.48, 7.75–7.77 (all m, 5H, Ph); 11.45 (s, 1H, OH). ^19^F NMR (400 MHz, CDCl_3_) δ 100.45 (d, *J* 0.9 Hz, CF_3_). Anal. calcd. for C_12_H_10_F_3_N_3_O_2_: C, 50.53; H, 3.53; N, 14.73. Found: C, 50.44; H, 3.40; N, 14.58.

#### 3.1.2. Synthesis of 4-Aminopyrazoles **4a**–**j** (General Procedure)

*Method A.* Tin (II) chloride dihydrate (8 mmol, 0.18 g) was dissolved in a minimum quantity of concentrated hydrochloric acid, and 4-hydroxyiminopyrazoles **1a**–**c**,**f**–**i** (2 mmol) were added. The mixture was stirred for 2–3 h at r.t. or until a change in color of the initial compounds **1** was observed. The resulting solid was filtered off and washed by *n*-hexane to yield compounds **4a**–**c**,**f**–**i**.

*Method B.* A solution of 4-hydroxyiminopyrazoles **1c**–**e**,**j** (1 mmol) in EtOH (10 mL) and 50 μL of HCl was hydrogenated in the presence of 5 wt.% Pd/C catalyst (10% mmol) in a steel autoclave under a hydrogen pressure of 5–7 bar and r.t. for 4–5 h. The solid impurities were filtered off, and the solvent was removed in vacuo. The resulting compound **4c**–**e**,**j** was washed with CHCl_3_.

*4-Amino-3-(trifluoromethyl)-1-phenyl-1H-pyrazol-5-ol hydrochloride (***4a***).* Yield 0.42 g (78%, *method A*), orange powder, m.p. 126–127 °C. IR: *ν* 3574, 3504, 3080, 2953 (OH, NH_2_); 1620, 1561, 1539, 1515 (C=N, C=C); 1153–1087 (CF) cm^−1^. ^1^H NMR (400 MHz, DMSO-*d*_6_): *δ* 7.42–7.46, 7.52–7.56, 7.69–7.71 (all m, 5H, Ph); NH_2_ and OH are overlapped by Ph protons. ^1^H NMR (The ^1^H NMR spectrum was registered along with ^13^C NMR after standing overnight [M1]) (500 MHz, DMSO-*d*_6_): a mixture of ***AH***:***AS*** (77:23): *δ* 7.21–7.24 (m, 1H, Ph ***AS***); 7.23 (t, *J* 51.0 Hz, NH_3_^+^ ***AS***); 7.43–7.47 (m, 1H+2H, Ph ***AH***+***AS***); 7.53–7.57, 7.69–7.70 (both m, 4H, Ph ***AH***); 7.93–7.94 (m, 2H, Ph ***AS***); 9.42 (br.s, 3H, OH+NH_2_ ***AH***). ^13^C NMR [M1] (500 MHz, DMSO-*d*_6_): a mixture of ***AH***:***AS****: δ* 118.53; 120.57 (q, *J* 270.4 Hz, CF_3_); 122.78 (br. m); 124.27; 124.73; 128.13 (br. m); 128.79; 129.25; 137.14 (q, *J* 35.1 Hz, C–CF_3_); 138.79; 147.06 (br. m); 152.52. ^19^F NMR (400 MHz, DMSO-*d*_6_): *δ* 102.35 (s, CF_3_). Anal. calcd. for C_10_H_9_ClF_3_N_3_O: C, 42.95; H, 3.24; N, 15.03; Cl, 12.68. Found: C, 42.94; H, 3.25; N, 15.02; Cl, 12.72.

*4-Amino-3-(pentafluoroethyl)-1-phenyl-1H-pyrazol-5-ol hydrate hydrochloride (***4b***).* Yield 0.52 g (75%, *method A*), light yellow powder, m.p. 176–178 °C. IR: *ν* 3057, 2884 (OH, NH_2_); 1616, 1529, 1506, 1486 (C=N, C=C); 1138–1106 (CF) cm^−1^. ^1^H NMR (500 MHz, DMSO-*d*_6_) *δ* 7.43–7.46, 7.54–7.57, 7.69–7.71 (all m, 5H, Ph); NH_2_ and OH are not observed due to deuterium exchange. ^1^H NMR [M1] (500 MHz, DMSO-*d*_6_): a mixture of ***AH***:***AS*** (40:60): *δ* 5.60 (br.s, 3H, OH+NH_2_ ***AH***); 7.22–7.24 (m, 1H, Ph ***AS***); 7.24 (t, *J* 51.0 Hz, NH_3_^+^ ***AS***); 7.46–7.49 (m, 2H, Ph ***AS***); 7.49–7.43 (m, 1H, Ph ***AH***); 7.55–7.58, 7.68–7.69 (both m, 4H, Ph ***AH***); 7.93–7.95 (m, 2H, Ph ***AS***). ^13^C NMR [M1] (500 MHz, DMSO-*d*_6_): a mixture of ***AH***:***AS****: δ* 118.33–113.00; 116.80–119.82 (both m, C_2_F_5_); 118.47; 121.79; 122.50 (br. m); 124.80; 125.02; 127.02; 127.97 (br. m); 128.81; 129.02; 129.25; 131.59–132.18 (m); 135.93 (t, *J* 26.1 Hz, C–C_2_F_5_); 137.10 (br. m); 138.11; 138.73; 147.09 (br. m); 152.34. ^19^F NMR (500 MHz, DMSO-*d*_6_): *δ* 51.96 (m, 2F, CF_2_); 80.07 (t, *J* 2.6 Hz, 3F, CF_3_). Anal. calcd. for C_11_H_11_ClF_5_N_3_O_2_: C, 38.00; H, 3.19; N, 12.09; Cl, 10.20. Found: C, 37.79; H, 2.98; N, 11.98; Cl, 10.41.

*5-Hydroxy-1-phenyl-3-(nonafluorobuthyl)-1H-pyrazol-4-ammonium hydrochloride (***4c***).* Yield 0.56 g (65%, *method A*), 0.39 g (45%, *method B*), orange powder, m.p. 174–175 °C. IR: *ν* 3235, 3143, 3049, 2823 (OH, NH_2_); 1586, 1515, 1493, 1460 (C=N, C=C); 1133–1228 (CF) cm^−1^. ^1^H NMR (500MHz, DMSO-d_6_): *δ* 7.15 (t, 3H, NH_3_^+^, *J* 51.0 Hz); 7.21–7.25, 7.45–7.48, 7.93–7.94 (all m, 5H, Ph); OH is not observed due to deuterium exchange. ^19^F NMR (500MHz, DMSO-*d*_6_) *δ* 36.79–36.84, 40.34–40.40, 51.36–51.40 (all m, 6F, 3 CF_2_); 81.95 (t, *J* 8.5 Hz, 3F, CF_3_). Anal. calcd. for C_13_H_9_ClF_9_N_3_O: C, 36.34; H, 2.11; N, 9.78; Cl, 8.25. Found: C, 36.13; H, 1.99; N, 9.53; Cl, 8.46.

*4-Amino-3-methyl-1-phenyl-1H-pyrazol-5-ol hydrochloride hydrate (***4d***).* Yield 0.37 g (77%, *method B*), orange powder, m.p. 171 °C dec. IR: *ν* 3441, 3381, 3068, 2839 (OH, NH_2_); 1638, 1592, 1540, 1487 (C=N, C=C). ^1^H NMR (500 MHz, DMSO-d_6_): *δ* 2.26 (s, 3H, Me); 7.27–7.30, 7.46–7.50, 7.70–7.71 (all m, 5H, Ph); 10.15 (br.s, 3H, NH_2_ and OH). ^13^C NMR (500 MHz, DMSO-*d*_6_): *δ* 11.24; 96.81; 120.20; 125.82; 129.07 (2C); 137.29; 142.87. Anal. calcd. for C_10_H_14_ClN_3_O_2_: C, 49.29; H, 5.79; N, 17.24; Cl, 14.55. Found: C, 49.03; H, 5.74; N, 17.26, Cl, 14.68.

*4-Amino-3-(trifluoromethyl)-1H-pyrazol-5-ol hydrochloride (***4e***).* Yield 0.31 g (78%, *method B*), orange powder, **m.p.** 200–201 °C (lit. [35] m.p. 194–195 °C). IR: *ν* 3124, 3055, 2872 (OH, NH_2_); 1586, 1567, 1544, 1505 (C=N, C=C); 1147–1085 (CF) cm^−1^. ^1^H NMR (400 MHz, DMSO-*d*_6_): *δ* 10.43, 13.39 (both br.s, 4H, OH and NH_2_). ^13^C NMR (500 MHz, DMSO-*d*_6_): *δ* 92.76; 120.55 (q, *J* 268.9 Hz, CF_3_); 133.01 (m, C—CF_3_); 148.84. ^19^F NMR (400 MHz, DMSO-*d*_6_): *δ* 102.66 (s, CF_3_). Anal. calcd. for C_4_H_5_ClF_3_N_3_O: C, 23.60; H, 2.48; N, 20.64; Cl 17.42. Found: C, 23.37; H, 2.44; N, 20.19; Cl 17.56.

*4-Amino-3-(1,1,2,2-tetrafluoroethyl)-1H-pyrazol-5-ol hydrate hydrochloride (***4f***).* Yield 0.38 g (75%, *method A*), white powder, m.p. 144 °C dec. IR: *ν* 3198, 2963, 2802, 2680 (OH, NH_2_); 1592, 1571, 1523, 1511 (C=N, C=C); 1152–1089 (CF) cm^−1^. ^1^H NMR (400 MHz, DMSO-*d*_6_): *δ* 6.84 (t, *J* 51.4 Hz, 1H, HCF_2_); 10.12 (br.s, 3H, NH_2_ and OH), 13.34 (br.s, 1H, NH). ^19^F NMR (400 MHz, DMSO-*d*_6_): *δ* 24.90 (m, 2F, CF_2_); 49.78 (m, 2F, CF_2_). Anal. calcd. for C_5_H_8_ClF_4_N_3_O_2_: C, 23.68; H, 3.18; N, 16.57; Cl, 13.98. Found: C, 23.55; H, 2.98; N, 16.31; Cl, 14.05.

*4-Amino-3-(pentafluoroethyl)-1H-pyrazol-5-ol hydrate hydrochloride (***4g***).* Yield 0.36 g (66%, *method A*), white powder, m.p. 160 °C dec. IR: *ν* 3133, 2956, 2798, 2697 (OH, NH_2_); 1589, 1570, 1517, 1502 (C=N, C=C); 1147–1103 (CF) cm^−1^. ^1^H NMR (500 MHz, DMSO-*d*_6_): *δ* 10.56 (br.s, 3H, NH_2_ and OH); 13.56 (br.s, 1H, NH). ^19^F NMR (500 MHz, DMSO-*d*_6_): *δ* 52.30 (m, 2F, CF_2_); 79.90 (m, 3F, CF_3_). Anal. calcd. for C_5_H_7_ClF_5_N_3_O_2_: C, 22.11; H, 2.60; N, 15.47; Cl, 13.05. Found: C, 21.78; H, 2.25; N, 15.11; Cl, 13.42.

*4-Amino-3-(heptafluoropropyl)-1H-pyrazol-5-ol hydrate hydrochloride (***4h***).* Yield 0.46 g (72%, *method A*), white powder, m.p. 146 °C subl. IR: 3103, 3007, 2942, 2806 (OH, NH_2_); 1618, 1590, 1571 (C=N, C=C); 1149–1087 (C-F). ^1^H NMR (400 MHz, DMSO-*d*_6_): *δ* 9.72 (br. s, 3H, NH_2_ and OH); 13.54 (br.s, 1H, NH). ^19^F NMR (400 MHz, DMSO-*d*_6_): *δ* 36.89 (m, 2F, β-CF_2_), 53.80 (m, 2F, α-CF_2_), 83.06 (t, 3F, CF_3,_ *J* 9.0 Hz). Anal. calcd. for C_6_H_7_ClF_7_N_3_O_2_: C, 22.41; H, 2.19; N, 13.07; Cl, 11.02. Found: C, 22.22; H, 2.07; N, 12.96; Cl, 11.25.

*4-Amino-3-(nonafluorobutyl)-1H-pyrazol-5-ol hydrate hydrochloride (***4i***).* Yield 0.50 g (68%, *method A*), white powder, m.p. 188–190 °C subl. IR: *ν* 3512, 3104, 2873, 2615 (OH, NH_2_); 1589, 1500 (C=N, C=C); 1230–1101 (CF) cm^−1^. ^1^H NMR (DMSO-d_6_): *δ* 10.11 (br.s, 1H, NH). ^19^F NMR (DMSO-d_6_): *δ* 37.48 (m, 2F, γ-CF_2_); 40.71 (d.d, *J* 17.1, 8.2 Hz, 2F, β-CF_2_); 54.61 (m, 2F, α-CF_2_); 82.11 (t, *J* 9.4 Hz, 3F, CF_3_). Anal. calcd. for C_7_H_7_ClF_9_N_3_O_2_: C, 22.63; H, 1.90; N, 11.31; Cl, 9.54. Found: C, 22.68; H, 1.85; N, 11.11; Cl, 9.39.

*4-Amino-3-methyl-1H-pyrazol-5-ol hydrochloride (***4j***).* Yield 0.20 g (67%, *method B*), orange powder, m.p. 200 °C dec (lit. [84] m.p. 200–203 °C). IR: 3222, 2877, 2694, 2664 (OH, NH_2_), 1674, 1639, 1590, 1579, 1556, 1532 (C=N, C=C). ^1^H NMR (500 MHz, DMSO-*d*_6_): *δ* 2.18 (s, 3H, Me); 9.84, 11.06 (both br.s, 3H, NH_2_ and OH). ^13^C NMR (500 MHz, DMSO-*d*_6_): *δ* 9.55; 95.47; 134.06; 154.34. Anal. calcd. for C_4_H_8_ClN_3_O: C, 32.12; H, 5.39; N, 28.09; Cl, 23.70. Found: C, 31.94; H, 5.38; N, 28.29; Cl, 23.90.

Copies of NMR spectra of compounds are available in Supplementary Material.

### 3.2. Biochemical Methods

#### 3.2.1. ABTS Radical Cation Scavenging Activity Assay

Radical scavenging activity of the compounds was assessed using the ABTS radical cation (ABTS^•+^) decolorization assay [56] with some modifications [57]. ABTS (2,2′-azinobis-(3-ethylbenzothiazoline-6-sulfonic acid)) was purchased from Tokyo Chemical Industry Co., Ltd. (Tokyo, Japan), and potassium persulfate (dipotassium peroxodisulfate), Trolox (6-hydroxy-2,5,7,8-tetramethylchroman-2-carboxylic acid), and HPLC-grade ethanol and DMSO were obtained from Sigma-Aldrich (Saint Louis, MO, USA). Aqueous solutions were prepared using deionized water. All test compounds were dissolved in DMSO.

The solution of cation radical ABTS^•+^ was produced by incubation of an aqueous solution of 7 mM ABTS and 2.45 mM potassium persulfate solution in equal quantities for 12–16 h at room temperature in the dark. Radical scavenging capacity of the compounds was analyzed by mixing 10 μL of compound with 240 μL of ABTS^•+^ working solution in ethanol (100 μM final concentration). Data were given for 1 h of incubation of compounds with ABTS^•+^. The reduction in absorbance was measured spectrophotometrically at 734 nm using an xMark microplate UV/VIS microplate spectrophotometer (Bio-Rad, Hercules, CA, USA).

Ethanol blanks were run in each assay. Values were obtained from three replicates of each sample and three independent experiments.

Standard antioxidant Trolox was used as a reference compound. Antioxidant capacity as Trolox equivalent (TEAC) values was determined as the ratio between the slopes obtained from the linear correlation of the ABTS radical absorbance with the concentrations of tested compounds and Trolox. Quercetin was used as a positive control. For the test compounds that reduced ABTS^•+^ absorbance by more than 60% at 100 µM, we also determined the IC_50_ values (the compound concentration required for a 50% reduction of the ABTS radical). The compounds were tested in the concentration range of 0.5–400 µM.

#### 3.2.2. The FRAP (Ferric Reducing Antioxidant Power) Assay

The FRAP (ferric reducing antioxidant power) assay measures the ability of antioxidants to reduce the ferric 2,4,6-tripyridyl-s-triazine complex [Fe (TPTZ)_2_]^3+^ to the intensely blue ferrous complex [Fe (TPTZ)_2_]^2+^ (λ = 593 nm) in acidic medium [58,59]. 2,4,6-Tris(pyridin-2-yl)-1,3,5-triazine (TPTZ), FeCl_3_·6H_2_O, Trolox, quercetin and DMSO were obtained from Sigma-Aldrich Chemical Co. (St. Louis, MO, USA). The FRAP reagent contained 2.5 mL of 10 mM TPTZ solution in 40 mM HCl, 2.5 mL of 20 mM FeCl_3_·6H_2_O water solution, and 25 mL of 0.3 M acetate buffer (pH 3.6). Aliquots of 10 µL of the test compound solution in DMSO (0.5 mM) were mixed with 240 µL of FRAP reagent, and the absorbance of the mixture was measured spectrophotometrically (FLUOStar OPTIMA microplate spectrophotometer (BMG Labtech, Ortenberg, Germany)) at 600 nm after 1 h incubation at 37 °C against a blank. Trolox was used as a reference compound. Quercetin was used as a positive control. A calibration curve with linear formula y = 0.03x (R^2^ = 0.998, *p* < 0.0001) was prepared for Trolox solutions in DMSO at a range of 2.5–100 µM, and the results are expressed as Trolox equivalents (TE), the values calculated as the ratio of the concentrations of Trolox and the test compound, resulting in the same effect.

#### 3.2.3. ORAC Assay

AAPH (2,2’-azobis(2-amidinopropane) dihydrochloride), Trolox, and fluorescein sodium salt were purchased from Sigma-Aldrich, USA. PBS (phosphate-buffered saline) was from VWR Life Science. DMSO (dimethylsulfoxide) was obtained from Vekton, Russia.

The oxygen radical absorbance capacity (ORAC) was determined by the ability of compounds to protect fluorescein (FL) from peroxyl radicals that are generated by AAPH according to the known method [60,85] with minor variations. The reaction was carried out at 37 °C in black 96-well plates (SPL life sciences), and the final volume of the reaction mixture was 200 µL per well. The fluorescence was recorded by Tecan M1000 PRO microplate reader (Switzerland) at 485 nm excitation and 520 nm emission.

The 0.7 mM fluorescein sodium salt stock solution in PBS pH 7.4 (137 mM sodium chloride, 2.7 mM potassium chloride, and 10 mM phosphate buffer) was prepared and stored at 4–5 °C no longer than one month. An aliquot of the fluorescein stock solution was further diluted with PBS immediately before each assay to yield a 70 nM working solution. The 133 mM AAPH solution in PBS was prepared immediately before use. The test compounds and Trolox standard were dissolved in DMSO to 400 μM. The final concentration was 10 μM for all of the samples. For IC_50_ determination, a set of compound concentrations in the range of 0.5–50 μM was used.

The blank was composed of 15 μL PBS pH 7.4, 5 μL DMSO (final concentration: 2.5% (*v*/*v*)), 120 μL FL, and 60 μL AAPH, and it was included in each assay. PBS pH 7.4 (15 μL), antioxidant (the test compound or Trolox, 5 μL), and FL (120 μL, final concentration: 42 nM) solutions were placed in a black 96-well microplate and were pre-incubated for 15 min at 37 °C. AAPH solution (60 μL, final concentration 40 mM) was then added rapidly using a multichannel pipette. The fluorescence was recorded every minute for 2 h. A Trolox standard curve was also obtained in each assay. All reactions were carried out in quadruplicate, and two to four independent assays were performed for each sample.

Antioxidant curves (fluorescence vs. time) were first normalized to the curve of the blank (without antioxidant) corresponding to the same assay, and the net AUC corresponding to a sample was calculated by subtracting the AUC corresponding to the blank. The ORAC value was obtained by dividing the latter curve by the Trolox curve obtained in the same assay at 10 μM concentrations of compounds and Trolox. Final ORAC values, Trolox equivalents (TE), were expressed as μmol test compounds per μmol Trolox, where the value of TE for Trolox was taken as 1. Data were expressed as means ± SEM.

#### 3.2.4. Inhibition In Vitro of Human Erythrocyte AChE, Equine Serum BChE, and Porcine Liver CES

Human erythrocyte acetylcholinesterase (AChE, EC 3.1.1.7), equine serum butyrylcholinesterase (BChE, EC 3.1.1.8), porcine liver carboxylesterase (CES, EC 3.1.1.1), acetylthiocholine iodide (ATCh), butyrylthiocholine iodide (BTCh), 4-nitrophenol acetate (4-NPA), and 5,5′-dithio-bis-(2-nitrobenzoic acid) (DTNB) were purchased from Sigma-Aldrich (Saint Louis, MO, USA).

All the kinetic experiments were performed under standard conditions, according to the protocol of IPAC RAS for a reversible inhibitors study.

Ellman’s colorimetric assay was used to measure AChE and BChE activity in 0.1 M K/Na phosphate buffer at pH 7.5, 25 °C [86]. Final concentrations of reactants were 0.33 mM DTNB, 0.02 unit/mL of AChE or BChE, and 1 mM of substrate (ATCh or BTCh, respectively). Reagent blanks consisted of reaction mixtures without enzyme to assess non-enzymatic hydrolysis of substrates. Porcine liver CES activity was assessed colorimetrically in 0.1 M K/Na phosphate buffer with pH 8.0 at 25 °C, by measuring the absorbance of 4-nitrophenol at 405 nm [87]. Final enzyme and substrate (4-NPA) concentrations were 0.02 unit/mL and 1 mM, respectively. Reagent blanks included all constituents except enzyme.

Test compounds were dissolved in DMSO. Reaction mixtures contained a final DMSO concentration of 2% (*v*/*v*). Enzyme inhibition was first assessed at a single concentration of 20 µM for each compound after a 10 min incubation at 25 °C in three separate experiments. Compounds inhibiting the enzyme by more than 30% were then selected for determination of the IC_50_ (inhibitor concentration resulting in 50% inhibition of control enzyme activity). Compounds (eight concentrations ranging between 10^−11^ and 10^−4^ M) were selected to achieve 20 to 80% inhibition and were incubated with each enzyme for 10 min at 25 °C. Substrate was then added, and residual enzyme activity relative to an inhibitor-free control was measured using a FLUOStar Optima microplate reader (LabTech, Ortenberg, Germany).

#### 3.2.5. Cytotoxicity Studies

Cytotoxicity of compounds was evaluated using a culture of human dermal fibroblasts, which were isolated in the Laboratory of Cell Cultures of the Institute of Medical Cell Technologies, Ekaterinburg, Russia. Cells were seeded in 96-well plates in the inoculum dose of 2 × 10^5^ cells/mL and cultured for 24 h in Dulbecco’s Modified Eagle Medium (DMEM), with 1% (w/v) glutamine in the presence of 10% (*v*/*v*) fetal bovine serum and gentamicin (50 mg/L) at 37 °C, with a humidified atmosphere of 5% (*v*/*v*) CO_2_. Then, compounds, which were solved in DMSO, were added to the wells at the final concentrations of 0.1, 1, 10, and 100 µM. The cells were incubated with compounds for 72 h, after which cell viability was assessed using the standard MTT assay [81]. The assay was carried out in four replicates with negative (culture medium) and positive (solution of the cytotoxic drug camptothecin at a concentration of 3 mM) controls, and the solvent control (DMSO). The results of the MTT assay were evaluated on a Tecan Infinite M200 PRO (Tecan Austria GmbH, Grödig, Austria) plate spectrophotometer by comparing the optical density of a formazan solution at 570 nm in the assay and control wells. The MTT staining of control cells was taken as 100%.

### 3.3. Quantum-Chemical Calculations

Adiabatic and vertical indices (electron affinity *EA*, ionization potential *IP*, and derived values) for 4-hydroxyiminopyrazolones **1a**–**j** were evaluated in the gas phase approximation (T 298 K and a pressure of 1 atm). The electronic parameters of the salts of 4-aminopyrazololes **4a**–**j** were calculated in the presence of the solvent (aqueous ethanol), because the effect of the solvent on cations is more pronounced than on neutral molecules. To obtain vertical values of electronic parameters, single-point calculations of anion and cation radicals were performed in the equilibrium geometry of the most stable conformer of a neutral species. In the case of salts **4a**–**j**, the cation structures were fully optimized, whereas single-point calculations were made for neutral or doubly charged species to estimate the *EA* or *IP* quantities, respectively. These indices for the studied compounds were calculated according to [88] using the following equations:

(1) $$\eta =\frac{1}{2}\left(IP-EA\right)$$

where *η* = the chemical hardness.

The ionization potential was estimated as the difference between the energies for the closed-shell N-electron and the open-shell (N − 1)-electron species [89]:

(2) $$IP=E\left(A^{•+}\right)-E\left(A\right)$$

The electron affinity of a molecule was determined in a similar manner using the open-shell (N + 1)-electron species:

(3) $$EA=E\left(A\right)-E\left(A^{•-}\right)$$

where *E* is the total energy *E_tot_* in the calculations of vertical values.

### 3.4. Statistical Analyses

Experimental data were expressed as means ± SEM (*n* = 3 independent experiments) using GraphPad Prism version 6.05 for Windows, GraphPad Software (San Diego, CA, USA). Linear regressions with coefficients of determination (*R*^2^) were determined using Origin 6.1 for Windows (OriginLab, Northampton, MA, USA). Data were tested for normality (Gaussian distribution) using the D’Agostino–Pearson, Anderson–Darling, Shapiro–Wilks, and Kolmogorov–Smirnov tests as implemented in GraphPad Prism 9.4.1 for Windows GraphPad software (San Diego, CA, USA). The data from the ORAC antioxidant test passed all four normality tests, while the data from the FRAP antioxidant tests failed all four normality tests, and the data from the ABTS antioxidant test failed the Anderson–Darling normality test. Therefore, determinations of correlation coefficients between the three pairs of antioxidant data were carried out using the non-parametric Spearman ρ rather than the parametric Pearson *r*, again using Prism 9.4.1 for Windows. Statistical significance was set at *p* < 0.05.

## 4. Conclusions

We have proposed effective approaches for the reduction of 4-hydroxyiminopyrazol-5-ones, which allowed us to obtain a series of new 4-aminopyrazol-5-ols as analogs of Edaravone. It was found that 4-aminopyrazol-5-ols can exist only as salts, while in free form they either decompose or transform into imino-bis-pyrazolols (rubazonic acids). The structure of the resulting aminopyrazolol salts was confirmed by IR and NMR spectroscopy along with elemental analysis.

The antioxidant action of 4-aminopyrazol-5-ol hydrochlorides and their precursors—4-hydroxyiminopyrazol-5-ones—was investigated in three test systems (ABTS, FRAP, and ORAC) in comparison with EDA, CF_3_-EDA, and Trolox. These studies revealed a pronounced antioxidant activity of aminopyrazolols in all tests. The lead compound was 4-amino-3-methyl-1-phenylpyrazol-5-ol hydrochloride (**4d**). Its activity in ABTS and FRAP tests was comparable to that of EDA, CF_3_-EDA, and Trolox, whereas in the ORAC test, it showed higher efficacy than the reference compounds. The similar character of the change in antioxidant activity values with the variation in the structure of the compounds in the ABTS and FRAP experiments allowed us to conclude that the transformations of the test compounds in these tests occurred by the SET mechanism, while in the ORAC test, the transformations took place via the HAT mechanism.

Using quantum-chemical calculations, in general, the salts of 4-aminopyrazolols **4** were found to be characterized by lower values of the calculated electronic characteristics [gap, ionization potential (*IP*), and chemical hardness (η)] [89] compared to 4-hydroxyiminopyrazol-5-ones **1**, and these results are consistent with their higher antioxidant activities determined experimentally in the ABTS and FRAP tests. Calculations showed that the most energetically favorable pathway was the formation of radicals through HO bond breaking in both 4-hydroxyiminopyrazolones **1** and 4-aminopyrazolols **4**. The transformations of 4-aminopyrazolols **4** bearing more groups that are prone to the formation of radicals were not as unambiguous and more dependent on structural fragments. Thus, the antioxidant activity of PhN-4-aminopyrazolols **4a**–**d** in the ABTS test was determined by gap functions, whereas, for HN-unsubstituted analogs, correlation with *IP* functions was found only for perfluoroalkyl derivatives **4e**,**h**–**i**. The antioxidant activity in the ORAC test directly depended on the OH bond dissociation energy for active 4-hydroxyiminopyrazolones **1e**–**j** and for all group of 4-aminopyrazolols **4a**–**j**.

The esterase profile results demonstrated the absence of anticholinesterase activity for all the compounds and a moderate inhibition of CES by some 4-aminopyrazolols **4**, especially for compounds **4c** and **4i** with a nonafluorobutyl substituent. The CES result should be taken into consideration in the potential therapeutic use of these compounds in high doses. In addition, the lead compound **4d** and its NH-analog **4j** did not show cytotoxicity against normal human fibroblasts, in contrast to EDA.

Thus, the investigation showed that 4-aminopyrazol-5-ols are promising antioxidant agents with activity at the Edaravone level and above. In particular, the lead compound **4d** displayed good antioxidant properties, indicating its promise for potential future use as a novel therapeutic drug candidate in the treatment of diseases associated with oxidative stress. Moreover, the new synthesized aminopyrazolols are of interest for further modification as antioxidant pharmacophores, e.g., for conjugation to an anticholinesterase fragment and for the creation of multifunctional drugs for the treatment of neurodegenerative diseases.

## Figures and Tables

**Table 1:** Antioxidant activity of pyrazoles **1** and **4**.

No.	Compound		Antioxidant Activity (Mean ± SEM)		
	R^1^	R^2^	ABTS *(n = 3; 1 h)*	FRAP *(n = 3; 1 h)*	ORAC-FL *(n = 3; 2 h)*
			TEAC * (IC_50_, μM) **	TE *	TE * (IC_50_, μM) **
	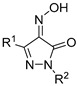				
**1a**	CF_3_	Ph	0.035 ± 0.002 (n.d.)	n.a.	n.a.
**1b**	C_2_F_5_	Ph	0.03 ± 0.002 (n.d.)	0.06 ± 0.01	n.a.
**1c**	C_4_F_9_	Ph	0.04 ± 0.004 (n.d.)	0.06 ± 0.01	n.a.
**1d**	Me	Ph	0.16 ± 0.02 (n.d.)	n.a.	1.07 ± 0.17
**1e**	CF_3_	H	0.23 ± 0.01 (n.d.)	0.07 ± 0.01	0.98 ± 0.03
**1f**	H(CF_2_)_2_	H	0.15 ± 0.007 (n.d.)	n.a.	n.d.
**1g**	C_2_F_5_	H	0.13 ± 0.04 (n.d.)	0.13 ± 0.01	1.87 ± 0.04
**1h**	C_3_F_7_	H	0.19 ± 0.008 (n.d.)	n.a.	2.37 ± 0.09
**1i**	C_4_F_9_	H	0.2 ± 0.01 (n.d.)	n.a.	2.41 ± 0.08
**1j**	Me	H	0.2 ± 0.03 (n.d.)	0.09 ± 0.01	2.85 ± 0.28
	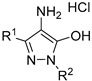				
**4a**	CF_3_	Ph	0.75 ± 0.04 (27.2 ± 2.0)	0.89 ± 0.05	3.49 ± 0.16 (8.8 ± 0.1)
**4b**	C_2_F_5_	Ph	0.6 ± 0.03 (34.1 ± 2.0)	0.75 ± 0.07	2.12 ± 0.17
**4c**	C_4_F_9_	Ph	0.85 ± 0.04 (23.4 ± 2.1)	0.98 ±0.05	2.82 ± 0.2
**4d**	Me	Ph	0.93 ± 0.03 (23.3 ± 1.9)	0.98 ± 0.08	4.39 ± 0.04 (6.85 ± 0.7)
**4e**	CF_3_	H	0.92 ± 0.04 (21.2 ± 1.8)	1.02 ± 0.02	0.90 ± 0.19
**4f**	H(CF_2_)_2_	H	0.50 ± 0.02 (33.2 ± 2.3)	0.62 ± 0.01	n.d.
**4g**	C_2_F_5_	H	0.73 ± 0.03 (25.7 ± 2.1)	0.88 ± 0.04	0.54 ± 0.03
**4h**	C_3_F_7_	H	0.78 ± 0.04 (24.8 ± 1.6)	1.03 ± 0.04	0.63 ± 0.04
**4i**	C_4_F_9_	H	0.45 ± 0.02 (40.8 ± 1.1)	0.75 ± 0.05	1.83 ± 0.02
**4j**	Me	H	0.95 ± 0.04 (20.1 ± 1.8)	1.04 ± 0.04	2.06 ± 0.16
CF_3_-EDA	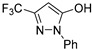		0.95 ± 0.04 (21.4 ± 0.8)	0.63 ± 0.03	3.89 ± 0.04 (6.3 ± 0.2)
EDA	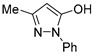		0.96 ± 0.04 (21.4 ± 1.1)	0.80 ± 0.01	3.71 ± 0.06 (5.6 ± 0.1)
Trolox			1.0 (20.1 ± 1.2)	1.0	1.0 (23.6 ± 4.4)
Quercetin			1.20 ± 0.11 (13.8 ± 0.7)	4.99 ± 0.02	5.41 ± 0.08

n.d.—not determined; n.a.—not active; * TEAC (Trolox equivalent antioxidant capacity, ABTS) and TE (Trolox equivalent, FRAP, ORAC)—activity of the compounds relative to Trolox (for calculation, see Experimental). ** compound concentration required for 50% reduction of the ABTS radical-cation or peroxyl radical.

**Table 2:** Esterase profile of pyrazoles **1** and **4**.

No.	Compound		Inhibitory Activity against AChE, BChE, and CES % Inhibition at 20 μM ^1^ or IC_50_, μM ^2^		
	R^1^	R^2^	AChE	BChE	CES
	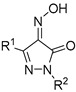				
**1a**	CF_3_	Ph	n.a.^3^	n.a.	n.a.
**1b**	C_2_F_5_	Ph	11.9 ± 0.9%	16.1 ± 1.4%	8.2 ± 1.3%
**1c**	C_4_F_9_	Ph	11.5 ± 1.8%	5.3 ± 1.0%	7.8 ± 1.4%
**1d**	Me	Ph	4.9 ± 1.3%	6.6 ± 1.3%	n.a.
**1e**	CF_3_	H	n.a.	n.a.	5.9 ± 1.1%
**1f**	H(CF_2_)_2_	H	6.8 ± 1.2%	4.7 ± 1.0%	3.5 ± 0.9%
**1g**	C_2_F_5_	H	n.a.	n.a.	7.8 ± 1.4%
**1h**	C_3_F_7_	H	5.3 ± 1.3%	n.a.	6.6 ± 1.4%
**1i**	C_4_F_9_	H	n.a.	n.a.	n.a.
**1j**	Me	H	n.a.	3.8 ± 1.3%	n.a.
	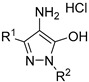				
**4a**	CF_3_	Ph	7.9 ± 1.5%	6.9 ± 1.1%	**44.9 ± 4.0**
**4b**	C_2_F_5_	Ph	4.4 ± 1.1%	12.8 ± 1.5%	16.6 ± 1.4%
**4c**	C_4_F_9_	Ph	19.2 ± 1.7%	10.3 ± 1.2%	**10.2 ± 0.8**
**4d**	Me	Ph	6.5 ± 1.2%	5.1 ± 1.3%	**98.7 ± 8.8**
**4e**	CF_3_	H	9.7 ± 1.5%	5.6 ± 1.5%	n.a.
**4f**	H(CF_2_)_2_	H	17.3 ± 1.5%	n.a.	17.5 ± 1.4%
**4g**	C_2_F_5_	H	17.3 ± 1.7%	8.9 ± 1.4%	6.7 ± 1.2%
**4h**	C_3_F_7_	H	n.a.	3.4 ± 0.9%	**81.8 ± 7.3**
**4i**	C_4_F_9_	H	5.1 ± 0.9%	n.a.	**21.8 ± 1.7**
**4j**	Me	H	n.a.	13.9 ± 1.6%	4.3 ± 0.9%
CF_3_-EDA	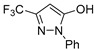		n.a.	n.a.	7.6 ± 1.2%
EDA	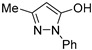		n.a.	4.6 ± 0.2%	8.4 ± 0.7%

^1^ Percentages correspond to percent inhibition ± SEM at 20 μM, *n* = 3. ^2^ Values without units of measurement (numbers printed in bold) correspond to IC_50_ ± SEM in μM, *n* = 3. ^3^ n.a.—not active.

**Table 3:** Vertical values (IP, EA, η) and BDE(OH) of compounds **1a**–**j**, **4a**–**j**.

R^1^	CF_3_	C_2_F_5_	C_4_F_9_	Me	CF_3_	(CF_2_)_2_H	C_2_F_5_	C_3_F_7_	C_4_F_9_	Me
R^2^	Ph	Ph	Ph	Ph	H	H	H	H	H	H
Compounds	4-Hydroxyiminopyrazolones									
	**1a**	**1b**	**1c**	**1d**	**1e**	**1f**	**1g**	**1h**	**1i**	**1j**
*IP*, eV	8.45	8.44	8.44	7.98	9.84	9.61	9.78	9.76	9.77	9.10
*EA*, eV	1.49	1.55	1.60	0.97	1.21	1.11	1.28	1.31	1.34	0.58
|HOMO-LUMO| (gap), eV	6.96	6.89	6.84	7.01	8.63	8.5	8.5	8.45	8.43	8.52
η, eV	3.48	3.45	3.42	3.50	4.32	4.25	4.25	4.22	4.21	4.26
BDE (OH), kJ/mol	315.4	314.3	314.3	314.0	314.0	320.7	313.0	312.9	313.0	312.7
Compounds	4-Aminopyrazolols									
	**4a**	**4b**	**4c**	**4d**	**4e**	**4f**	**4g**	**4h**	**4i**	**4j**
*IP*, eV	6.81	6.85	6.84	6.41	7.06	7.02	7.09	7.09	7.11	6.56
*EA*, eV	1.33	1.34	1.40	1.05	0.48	0.46	0.59	0.43	0.53	0.12
|HOMO-LUMO| (gap), eV	−5.47	−5.51	−5.44	−5.36	−6.58	−6.56	−6.50	−6.67	−6.58	−6.43
η, eV	2.74	2.75	2.72	2.68	3.29	3.28	3.25	3.33	3.29	3.22
BDE (OH), kJ/mol	318.9	319.9	319.4	302.3	330.1	328.0	330.7	330.2	329.8	309.2

*EA*—electron affinity, *IP*—ionization potential, HOMO—the highest occupied molecular orbital, LUMO—the lowest unoccupied molecular orbital; η—chemical hardness, BDE—bond dissociation energy.

**Table 4:** Cytotoxicity of EDA on NHDF.

EDA Inhibitory Capability (IC, %) at Increasing Concentrations		
1 µM	10 µM	100 µM
3.74 ± 0.05	19.9 ± 0.8	32.8 ± 0.6

## Data Availability

Not applicable.

## Supplementary Materials

The following information is available online at https://www.mdpi.com/article/10.3390/molecules27227722/s1: Figure S1: ^1^H NMR spectrum of bis[5-hydroxy-1-phenyl-3-(trifluoromethyl)-1H-pyrazol-4-yl]imine (**2a**); Figure S2: ^13^C NMR spectrum of bis[5-hydroxy-1-phenyl-3-(trifluoromethyl)-1H-pyrazol-4-yl]imine (**2a**); Figure S3: ^19^F NMR spectrum of bis[5-hydroxy-1-phenyl-3-(trifluoromethyl)-1H-pyrazol-4-yl]imine (**2a**); Figure S4: ^1^H NMR spectrum of bis[5-hydroxy-3-(pentafluoroethyl)-1-phenyl-1H-pyrazol-4-yl]imine (**2b**); Figure S5: ^19^F NMR spectrum of bis[5-hydroxy-3-(pentafluoroethyl)-1-phenyl-1H-pyrazol-4-yl]imine (**2b**); Figure S6: ^1^H NMR spectrum of N-(5-hydroxy-3-(trifluoromethyl)-1-phenyl-1H-pyrazol-4-yl)acetamide (**3a**); Figure S7: ^19^F NMR spectrum of N-(5-hydroxy-3-(trifluoromethyl)-1-phenyl-1H-pyrazol-4-yl)acetamide (**3a**); Figure S8: ^1^H NMR spectrum of 4-amino-3-trifluoromethyl-1-phenyl-1H-pyrazol-5-ol hydrochloride (**4a**); Figure S9: ^1^H NMR spectrum of 4-amino-3-trifluoromethyl-1-phenyl-1H-pyrazol-5-ol hydrochloride (**4a**) after standing overnight; Figure S10: ^13^C NMR spectrum of 4-amino-3-trifluoromethyl-1-phenyl-1H-pyrazol-5-ol hydrochloride (**4a**); Figure S11: ^19^F NMR spectrum of 4-amino-3-trifluoromethyl-1-phenyl-1H-pyrazol-5-ol hydrochloride (**4a**); Figure S12: ^1^H NMR spectrum of 4-amino-3-pentafluoroethyl-1-phenyl-1H-pyrazol-5-ol hydrate hydrochloride (**4b**); Figure S13: ^1^H NMR spectrum of 4-amino-3-pentafluoroethyl-1-phenyl-1H-pyrazol-5-ol hydrate hydrochloride (**4b**) after standing overnight; Figure S14: ^13^C NMR spectrum of 4-amino-3-pentafluoroethyl-1-phenyl-1H-pyrazol-5-ol hydrate hydrochloride (**4b**); Figure S15: ^19^F NMR spectrum of 4-amino-3-pentafluoroethyl-1-phenyl-1H-pyrazol-5-ol hydrate hydrochloride (**4b**); Figure S16: ^1^H NMR spectrum of 5-hydroxy-1-phenyl-3-nonafluorobuthyl-1H-pyrazol-4-ammonium hydrochloride (**4c**); Figure S17: ^19^F NMR spectrum of 5-hydroxy-1-phenyl-3-nonafluorobuthyl-1H-pyrazol-4-ammonium hydrochloride (**4c**); Figure S18: ^1^H NMR spectrum of 4-amino-3-methyl-1-phenyl-1H-pyrazol-5-ol hydrochloride hydrate (**4d**); Figure S19: ^13^C NMR spectrum of 4-amino-3-methyl-1-phenyl-1H-pyrazol-5-ol hydrochloride hydrate (**4d**); Figure S20: ^1^H NMR spectrum of 4-amino-3-trifluoromethyl-1H-pyrazol-5-ol hydrochloride (**4e**); Figure S21: ^13^C NMR spectrum of 4-amino-3-trifluoromethyl-1H-pyrazol-5-ol hydrochloride (**4e**); Figure S22: ^19^F NMR spectrum of 4-amino-3-trifluoromethyl-1H-pyrazol-5-ol hydrochloride (**4e**); Figure S23: ^1^H NMR spectrum of 4-amino-3-(1,1,2,2-tetrafluoroethyl)-1H-pyrazol-5-ol hydrate hydrochloride (**4f**); Figure S24: ^19^F NMR spectrum of 4-amino-3-(1,1,2,2-tetrafluoroethyl)-1H-pyrazol-5-ol hydrate hydrochloride (**4f**); Figure S25: ^1^H NMR spectrum of 4-amino-3-(pentafluoroethyl)-1H-pyrazol-5-ol hydrate hydrochloride (**4g**); Figure S26: ^19^F NMR spectrum of 4-amino-3-(pentafluoroethyl)-1H-pyrazol-5-ol hydrate hydrochloride (**4g**); Figure S27: ^1^H NMR spectrum of 4-amino-3-(heptafluoropropyl)-1H-pyrazol-5-ol hydrate hydrochloride (**4h**); Figure S28: ^19^F NMR spectrum of 4-amino-3-(heptafluoropropyl)-1H-pyrazol-5-ol hydrate hydrochloride (**4h**); Figure S29: ^1^H NMR spectrum of 4-amino-3-(nonafluorobutyl)-1H-pyrazol-5-ol hydrate hydrochloride (**4i**); Figure S30: ^19^F NMR spectrum of 4-amino-3-(nonafluorobutyl)-1H-pyrazol-5-ol hydrate hydrochloride (**4i**); Figure S31: ^1^H NMR spectrum of 4-amino-3-methyl-1H-pyrazol-5-ol hydrochloride (**4j**); Figure S32:^13^C NMR spectrum of 4-amino-3-methyl-1H-pyrazol-5-ol hydrochloride (**4j**).

## Sample Availability

Samples of the compounds **1a–j**, **2a**,**b**, **3a**, **4a–j** are available from the authors.
