# Supplementary material for: Synthesis of 4-Aminopyrazol-5-ols as Edaravone Analogs and Their Antioxidant Activity
Source: Molecules. 2022 Nov 9;27(22):7722. doi: 10.3390/molecules27227722 (PMC9699072; doi:10.3390/molecules27227722)

Synthesis of 4-Aminopyrazol-5-ols as Edaravone Analogs and Their Antioxidant Activity

Table of contents

Figure S1. ^{1}H NMR spectrum of bis[5-hydroxy-1-phenyl-3-(trifluoromethyl)-1H-pyrazol-4-yl]imine (**2a**)... 2

Figure S2. ^{13}C NMR spectrum of bis[5-hydroxy-1-phenyl-3-(trifluoromethyl)-1H-pyrazol-4-yl]imine (**2a**)... 3

Figure S3. ^{19}F NMR spectrum of bis[5-hydroxy-1-phenyl-3-(trifluoromethyl)-1H-pyrazol-4-yl]imine (**2a**). 4

Figure S4. ^{1}H NMR spectrum of bis[5-hydroxy-3-(pentafluoroethyl)-1-phenyl-1H-pyrazol-4-yl]imine (**2b**).. 5

Figure S5. ^{19}F NMR spectrum of bis[5-hydroxy-3-(pentafluoroethyl)-1-phenyl-1H-pyrazol-4-yl]imine (**2b**)....................................... 6

Figure S6. ^{1}H NMR spectrum of N-(5-hydroxy-3-(trifluoromethyl)-1-phenyl-1H-pyrazol-4-yl)acetamide (**3a**). 7

Figure S7. ^{19}F NMR spectrum of N-(5-hydroxy-3-(trifluoromethyl)-1-phenyl-1H-pyrazol-4-yl)acetamide (**3a**). 8

Figure S8. ^{1}H NMR spectrum of 4-amino-3-trifluoromethyl-1-phenyl-1H-pyrazol-5-ol hydrochloride (**4a**)... 9

Figure S9. ^{1}H NMR spectrum of 4-amino-3-trifluoromethyl-1-phenyl-1H-pyrazol-5-ol hydrochloride (**4a**) after standing overnight 10

Figure S10. ^{13}C NMR spectrum of 4-amino-3-trifluoromethyl-1-phenyl-1H-pyrazol-5-ol hydrochloride (**4a**) 11

Figure S11. ^{19}F NMR spectrum of 4-amino-3-trifluoromethyl-1-phenyl-1H-pyrazol-5-ol hydrochloride (**4a**)..................................... 12

Figure S12. ^{1}H NMR spectrum of 4-amino-3-pentafluoroethyl-1-phenyl-1H-pyrazol-5-ol hydrate hydrochloride (**4b**).................... 13

Figure S13. ^{1}H NMR spectrum of 4-amino-3-pentafluoroethyl-1-phenyl-1H-pyrazol-5-ol hydrate hydrochloride (**4b**) after standing overnight 14

Figure S14. ^{13}C NMR spectrum of 4-amino-3-pentafluoroethyl-1-phenyl-1H-pyrazol-5-ol hydrate hydrochloride (**4b**).................... 15

Figure S15. ^{19}F NMR spectrum of 4-amino-3-pentafluoroethyl-1-phenyl-1H-pyrazol-5-ol hydrate hydrochloride (**4b**) 16

Figure S16. ^{1}H NMR spectrum of 5-hydroxy-1-phenyl-3-nonafluorobuthyl-1H-pyrazol-4-ammonium hydrochloride (**4c**). 17

Figure S17. ^{19}F NMR spectrum of 5-hydroxy-1-phenyl-3-nonafluorobuthyl-1H-pyrazol-4-ammonium hydrochloride (**4c**). 18

Figure S18. ^{1}H NMR spectrum of 4-amino-3-methyl-1-phenyl-1H-pyrazol-5-ol hydrochloride hydrate (**4d**). 19

Figure S19. ^{13}C NMR spectrum of 4-amino-3-methyl-1-phenyl-1H-pyrazol-5-ol hydrochloride hydrate (**4d**).................................... 20

Figure S20. ^{1}H NMR spectrum of 4-amino-3-trifluoromethyl-1H-pyrazol-5-ol hydrochloride (**4e**). 21

Figure S21. ^{13}C NMR spectrum of 4-amino-3-trifluoromethyl-1H-pyrazol-5-ol hydrochloride (**4e**)... 22

Figure S22. ^{19}F NMR spectrum of 4-amino-3-trifluoromethyl-1H-pyrazol-5-ol hydrochloride (**4e**). 23

Figure S23. ^{1}H NMR spectrum of 4-amino-3-(1,1,2,2-tetrafluoroethyl)-1H-pyrazol-5-ol hydrate hydrochloride (**4f**). 24

Figure S24. ^{19}F NMR spectrum of 4-amino-3-(1,1,2,2-tetrafluoroethyl)-1H-pyrazol-5-ol hydrate hydrochloride (**4f**). 25

Figure S25. ^{1}H NMR spectrum of 4-amino-3-(pentafluoroethyl)-1H-pyrazol-5-ol hydrate hydrochloride (**4g**).................................. 26

Figure S26. ^{19}F NMR spectrum of 4-amino-3-(pentafluoroethyl)-1H-pyrazol-5-ol hydrate hydrochloride (**4g**).................................. 27

Figure S27. ^{1}H NMR spectrum of 4-amino-3-(heptafluoropropyl)-1H-pyrazol-5-ol hydrate hydrochloride (**4h**)............................... 28

Figure S28. ^{19}F NMR spectrum of 4-amino-3-(heptafluoropropyl)-1H-pyrazol-5-ol hydrate hydrochloride (**4h**). 29

Figure S29. ^{1}H NMR spectrum of 4-amino-3-(nonafluorobutyl)-1H-pyrazol-5-ol hydrate hydrochloride (**4i**). 30

Figure S30. ^{19}F NMR spectrum of 4-amino-3-(nonafluorobutyl)-1H-pyrazol-5-ol hydrate hydrochloride (**4i**). 31

Figure S31. ^{1}H NMR spectrum of 4-amino-3-methyl-1H-pyrazol-5-ol hydrochloride (**4j**)... 32

Figure S32. ^{13}C NMR spectrum of 4-amino-3-methyl-1H-pyrazol-5-ol hydrochloride (**4j**). 33

Figure S1. ^{1}H NMR spectrum of bis[5-hydroxy-1-phenyl-3-(trifluoromethyl)-1H-pyrazol-4-yl]imine (**2a**).

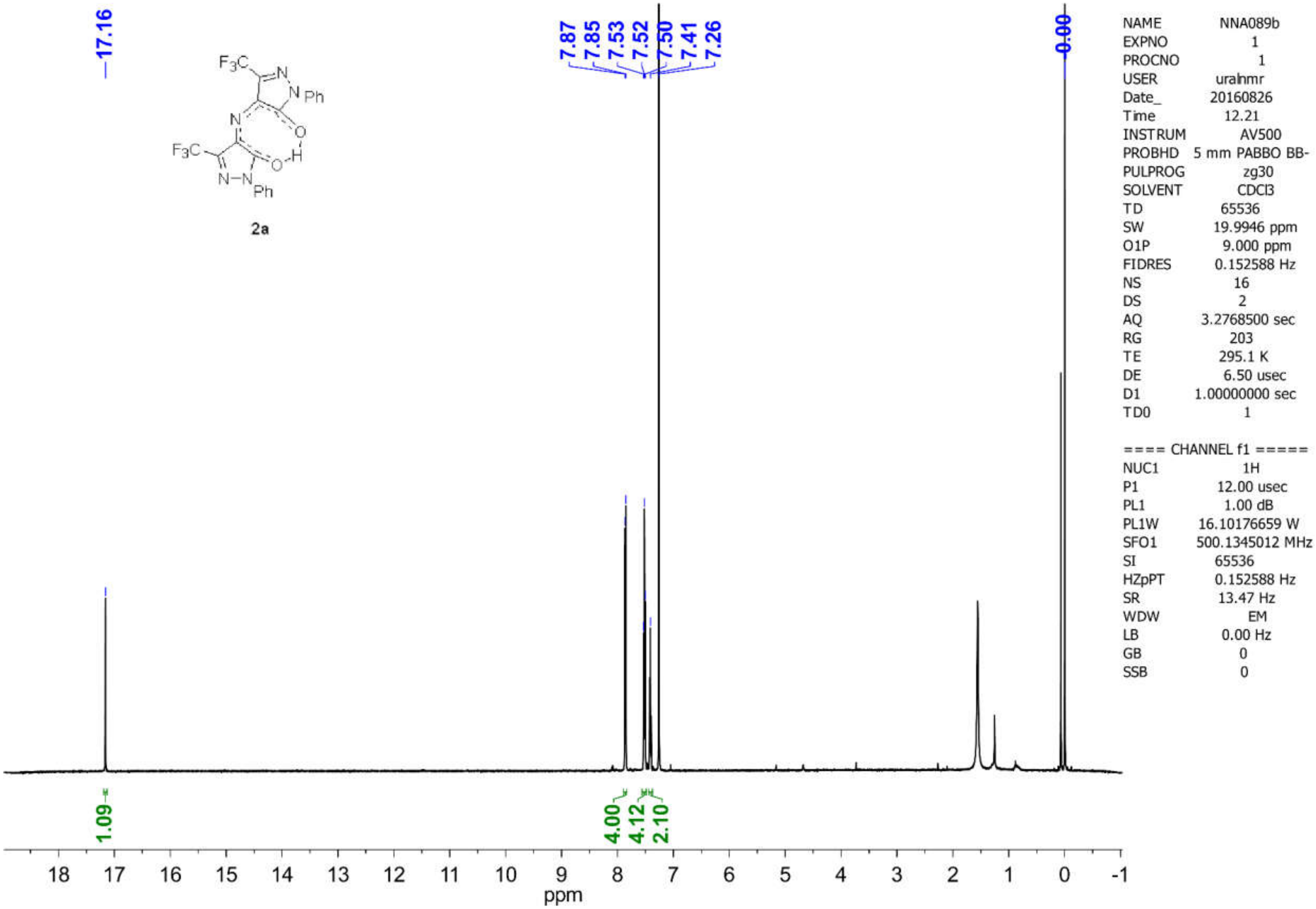

Figure S2. ^{13}C NMR spectrum of bis[5-hydroxy-1-phenyl-3-(trifluoromethyl)-1H-pyrazol-4-yl]imine (**2a**).

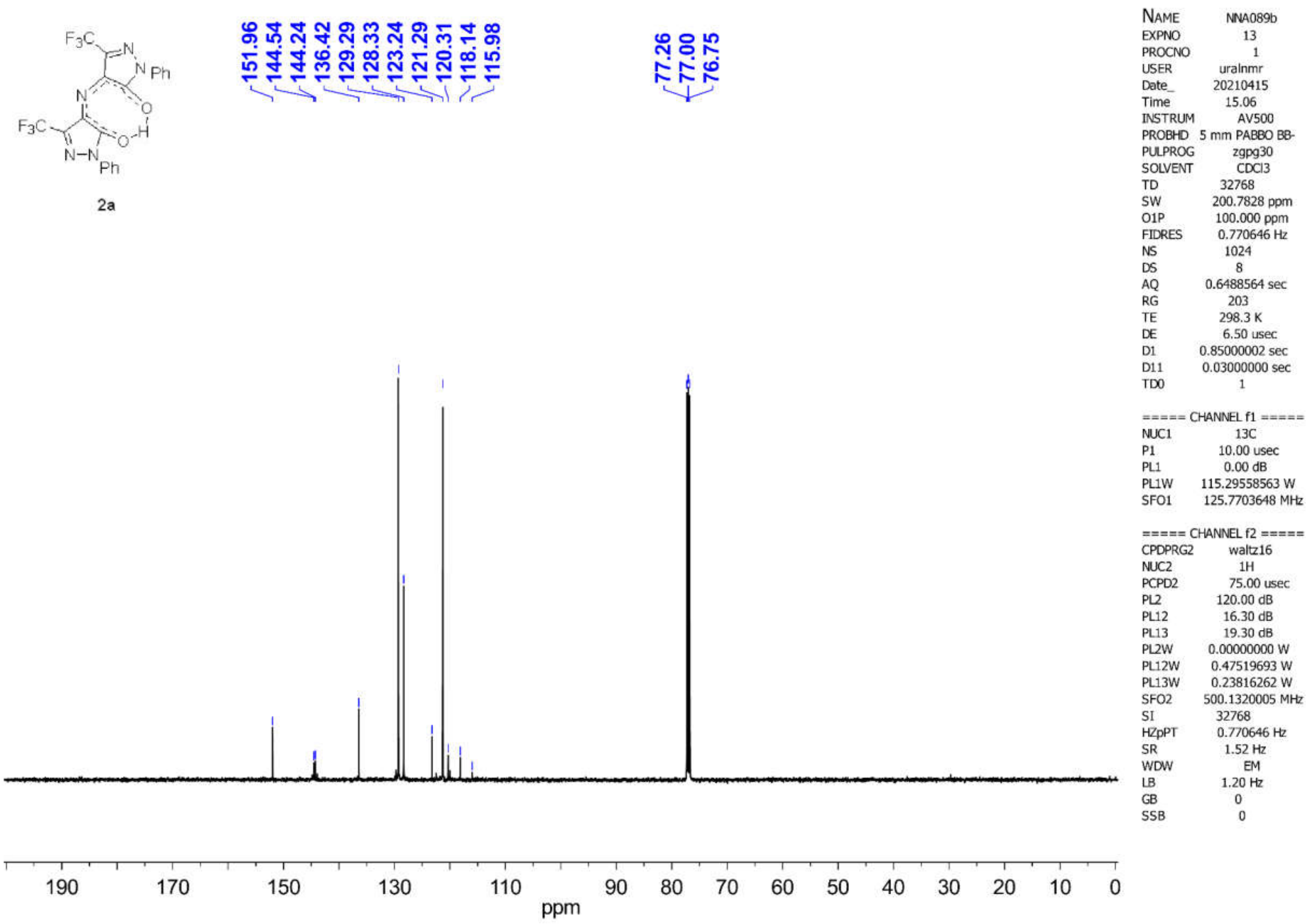

Figure S3. ^{19}F NMR spectrum of bis[5-hydroxy-1-phenyl-3-(trifluoromethyl)-1H-pyrazol-4-yl]imine (**2a**).

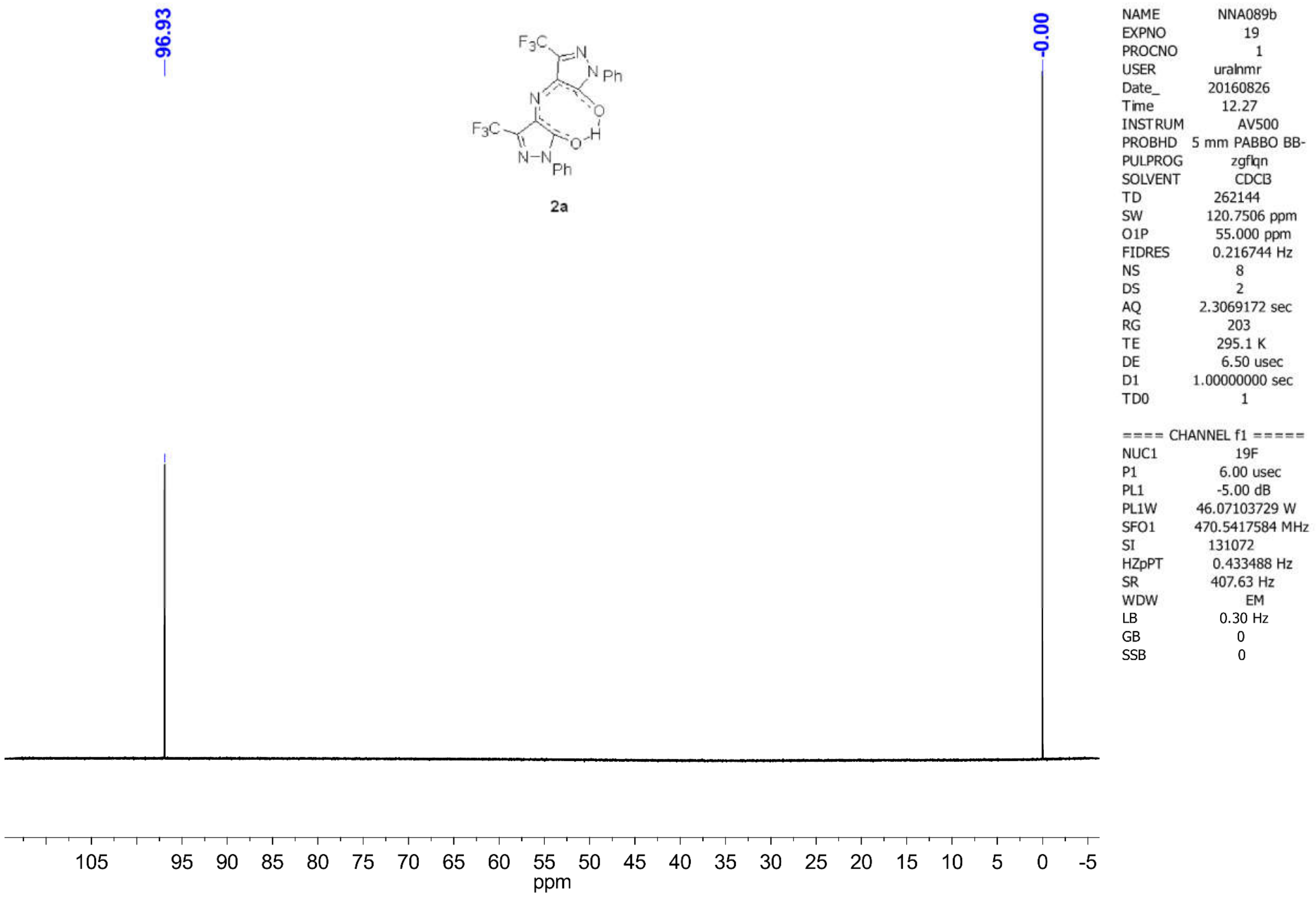

Figure S4. ^{1}H NMR spectrum of bis[5-hydroxy-3-(pentafluoroethyl)-1-phenyl-1H-pyrazol-4-yl]imine (**2b**).

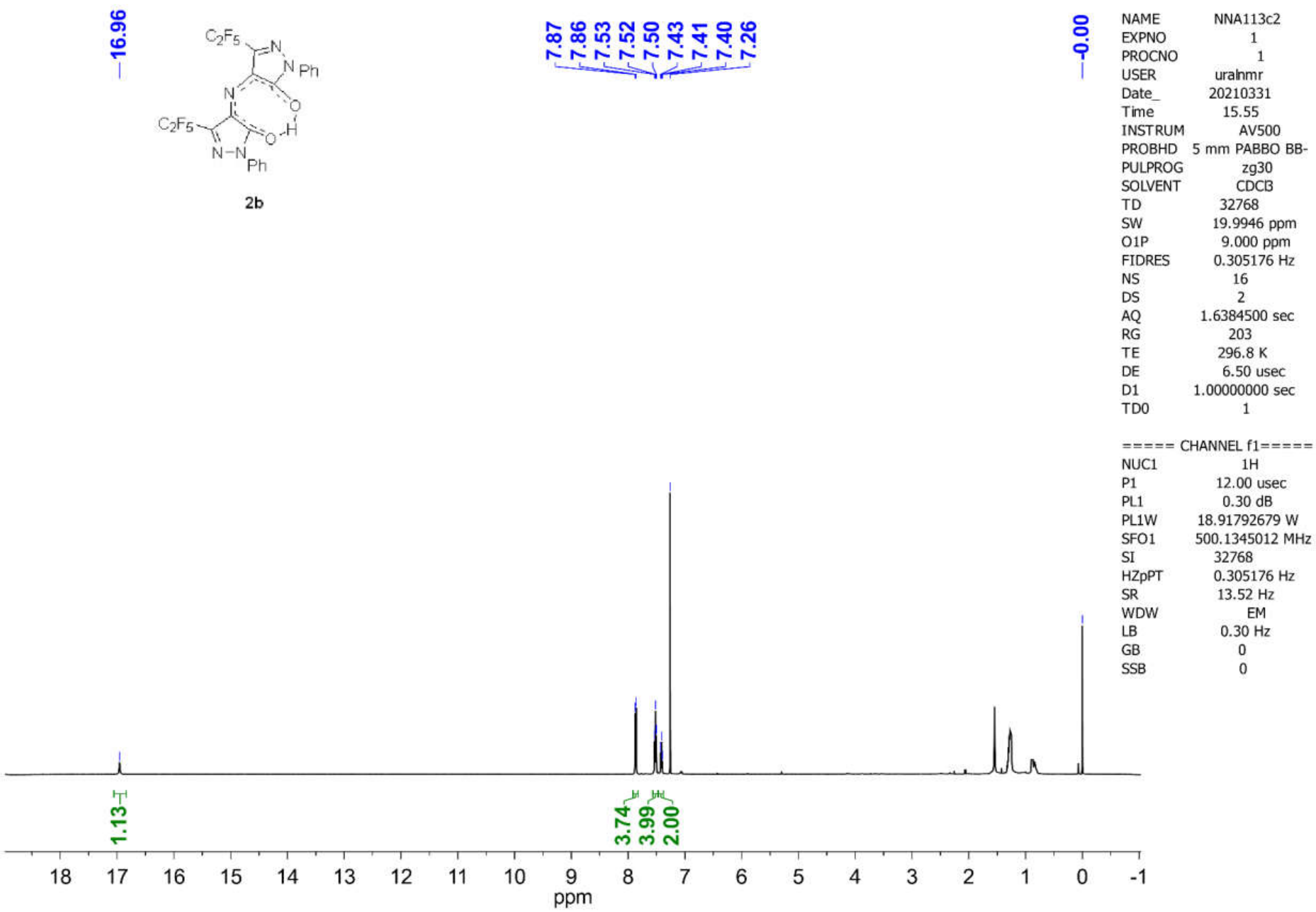

Figure S5. ^{19}F NMR spectrum of bis[5-hydroxy-3-(pentafluoroethyl)-1-phenyl-1H-pyrazol-4-yl]imine (**2b**).

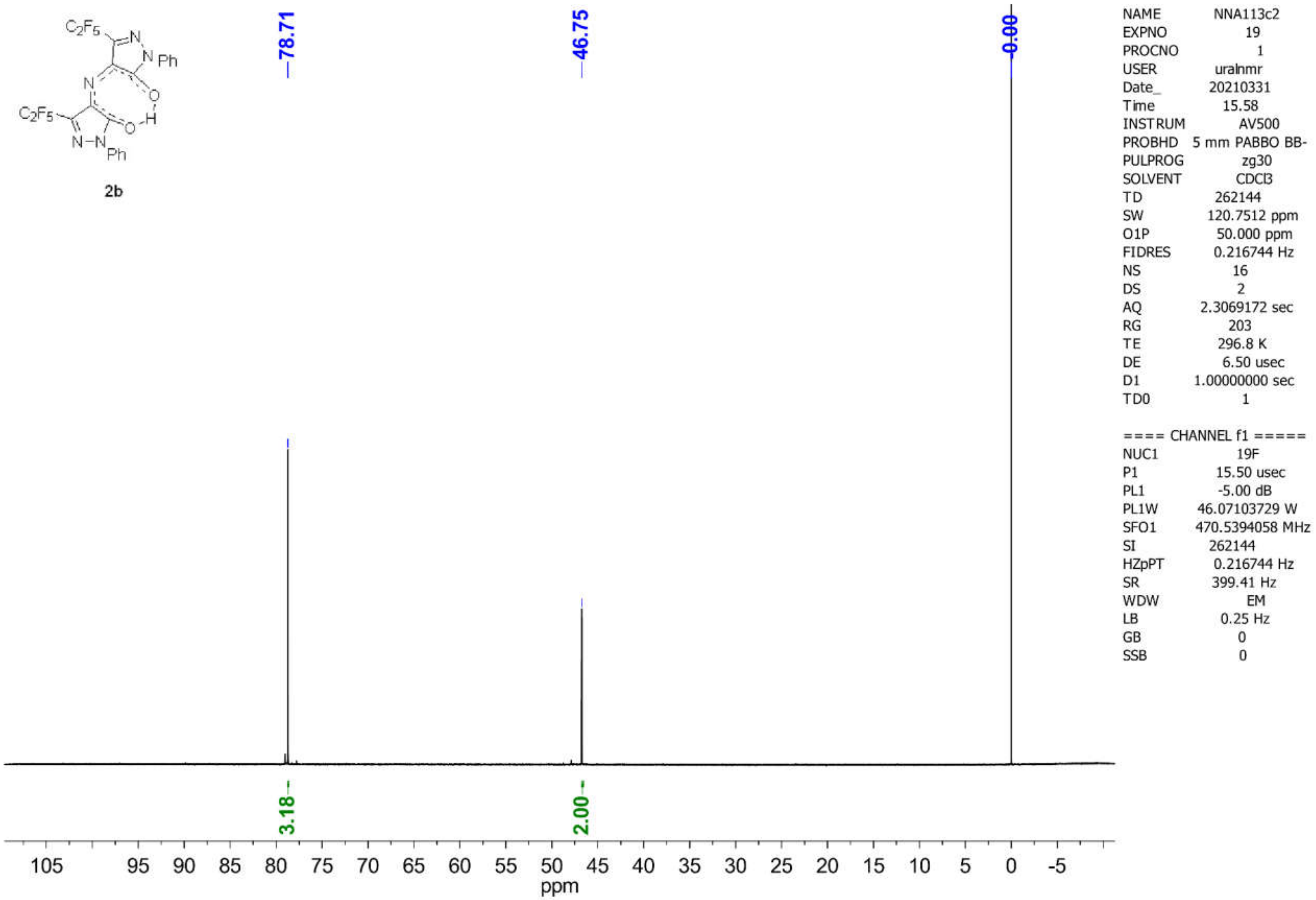

Figure S6. ^{1}H NMR spectrum of N-(5-hydroxy-3-(trifluoromethyl)-1-phenyl-1H-pyrazol-4-yl)acetamide (**3a**).

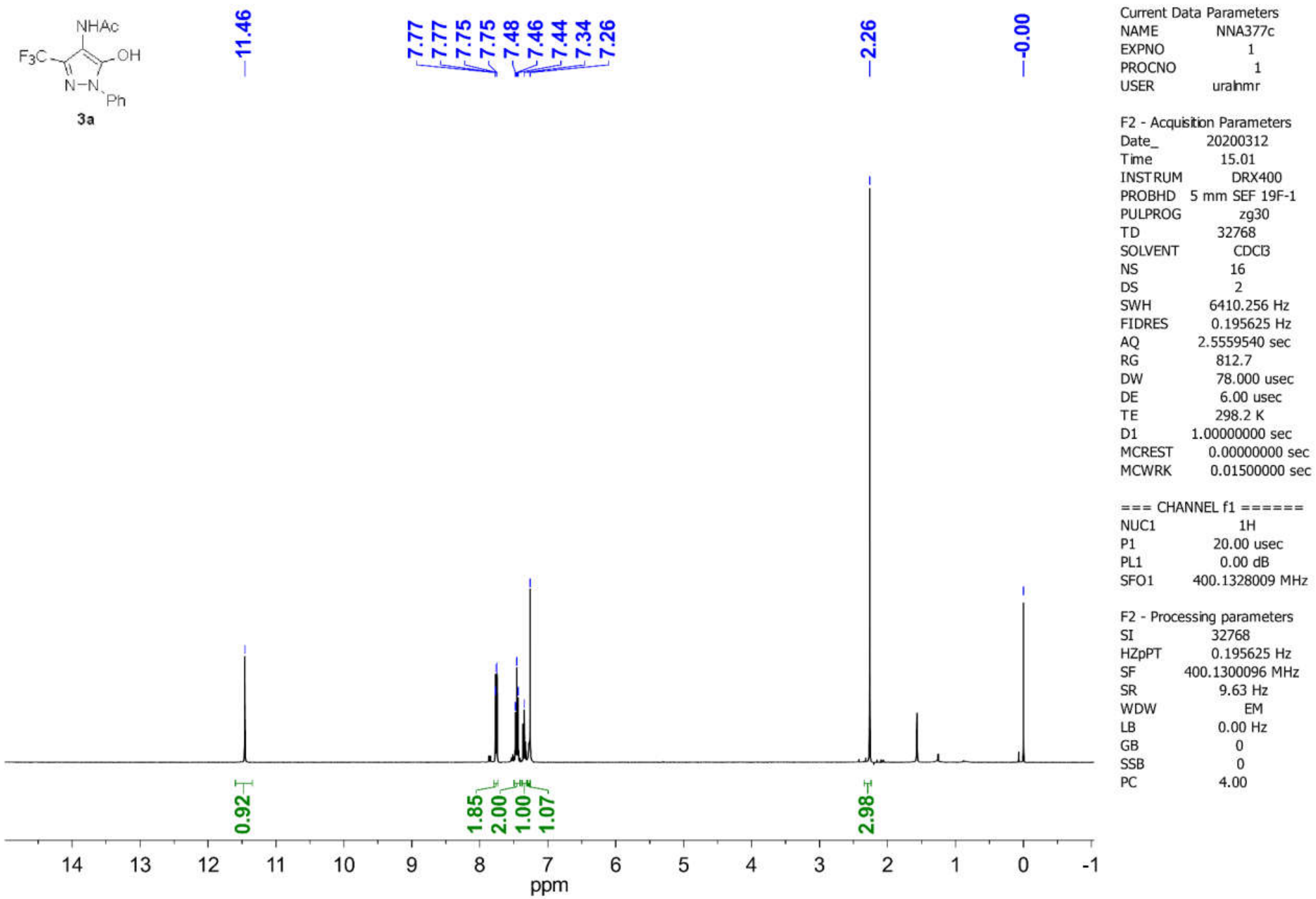

Figure S7. ^{19}F NMR spectrum of N-(5-hydroxy-3-(trifluoromethyl)-1-phenyl-1H-pyrazol-4-yl)acetamide (**3a**).

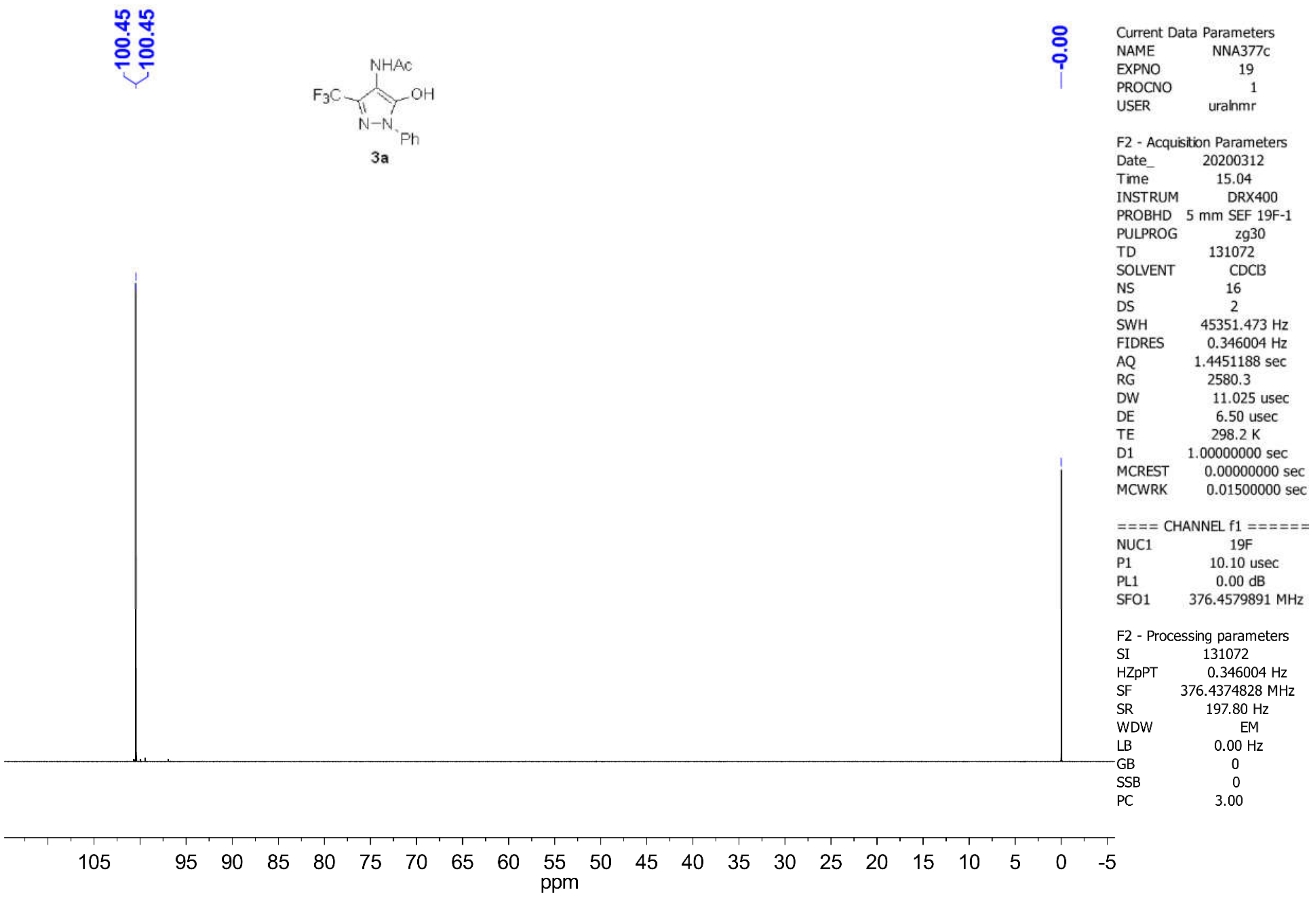

Figure S8. ^{1}H NMR spectrum of 4-amino-3-trifluoromethyl-1-phenyl-1H-pyrazol-5-ol hydrochloride (**4a**).

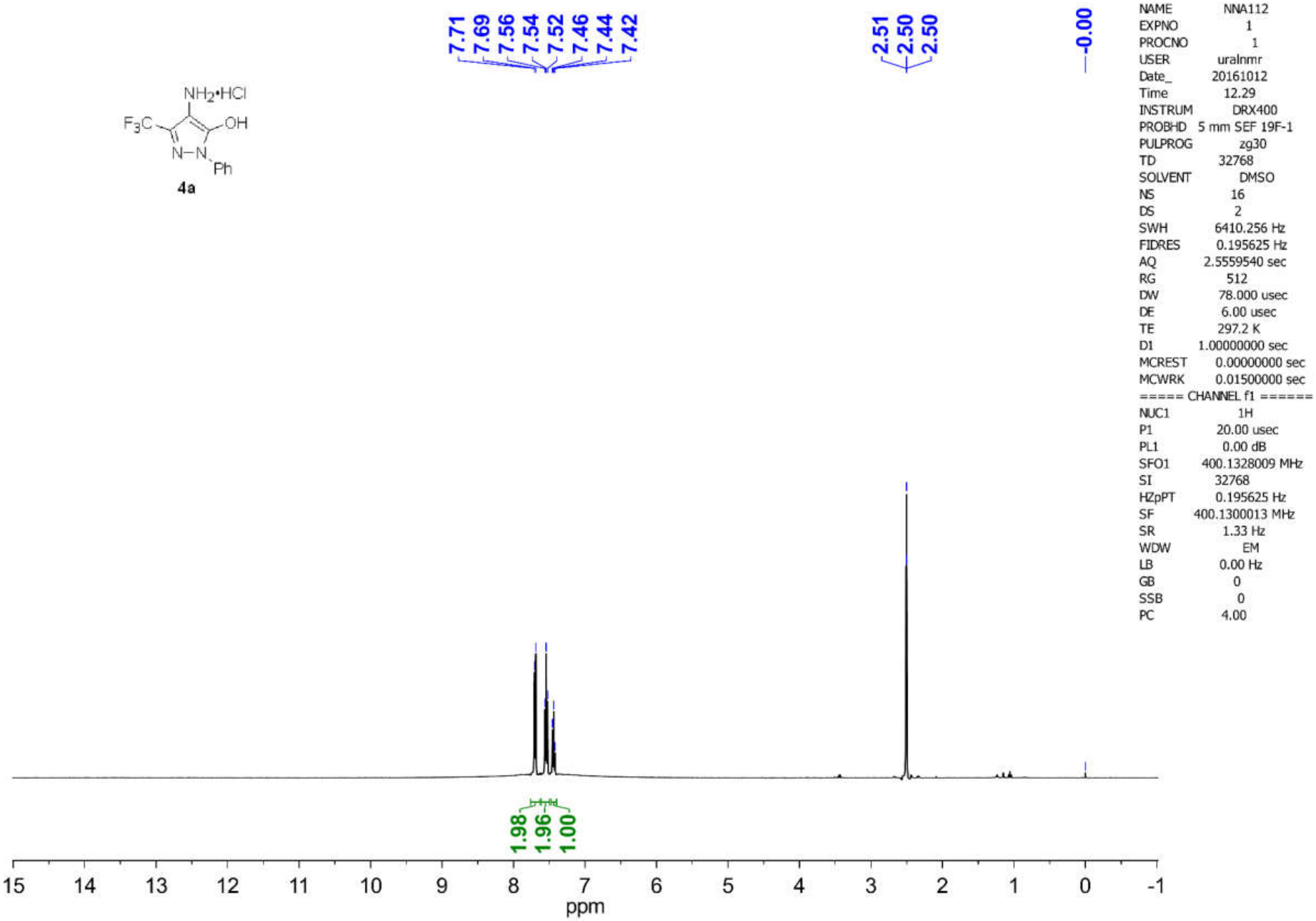

Figure S9. ^{1}H NMR spectrum of 4-amino-3-trifluoromethyl-1-phenyl-1H-pyrazol-5-ol hydrochloride (**4a**) after standing overnight.

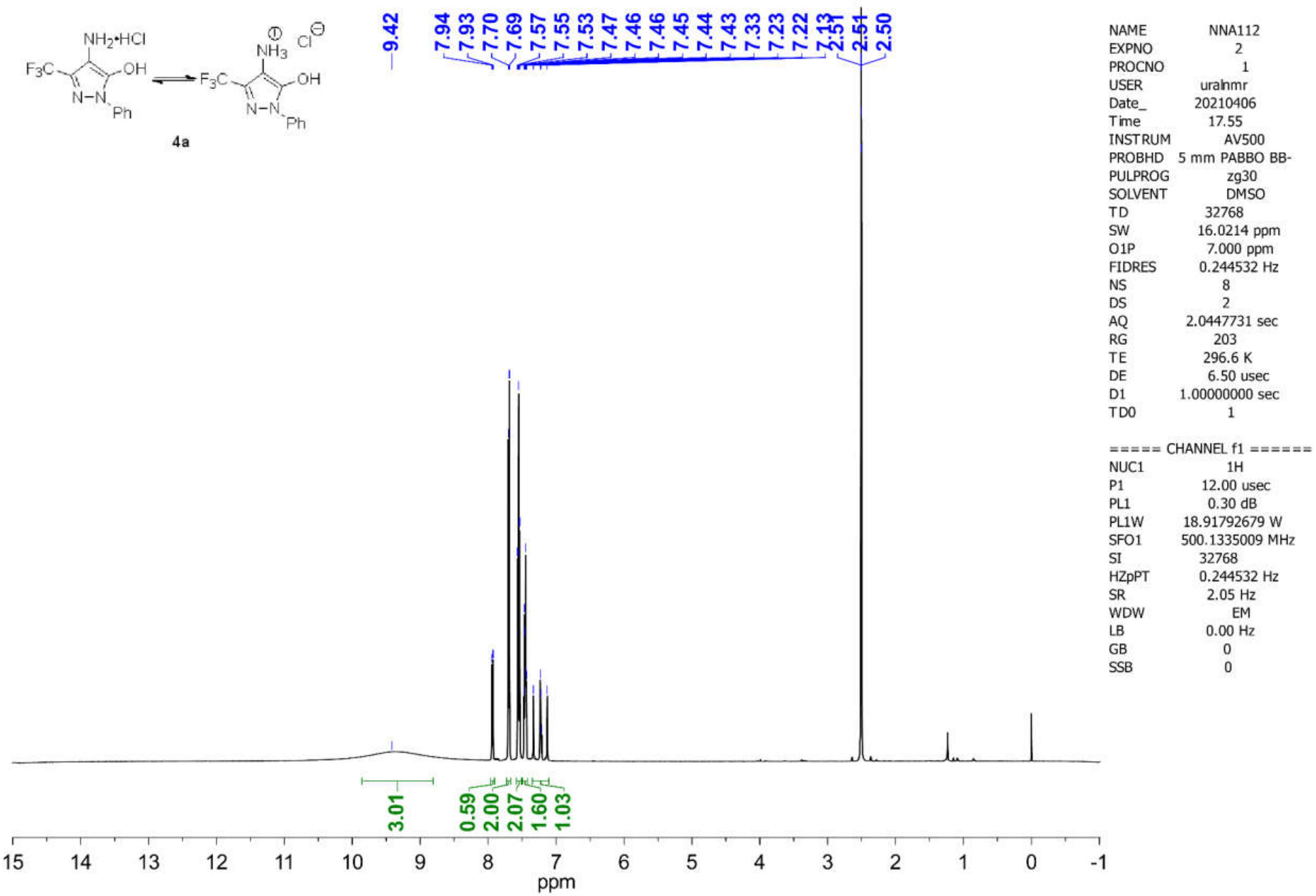

Figure S10. ^{13}C NMR spectrum of 4-amino-3-trifluoromethyl-1-phenyl-1H-pyrazol-5-ol hydrochloride (**4a**).

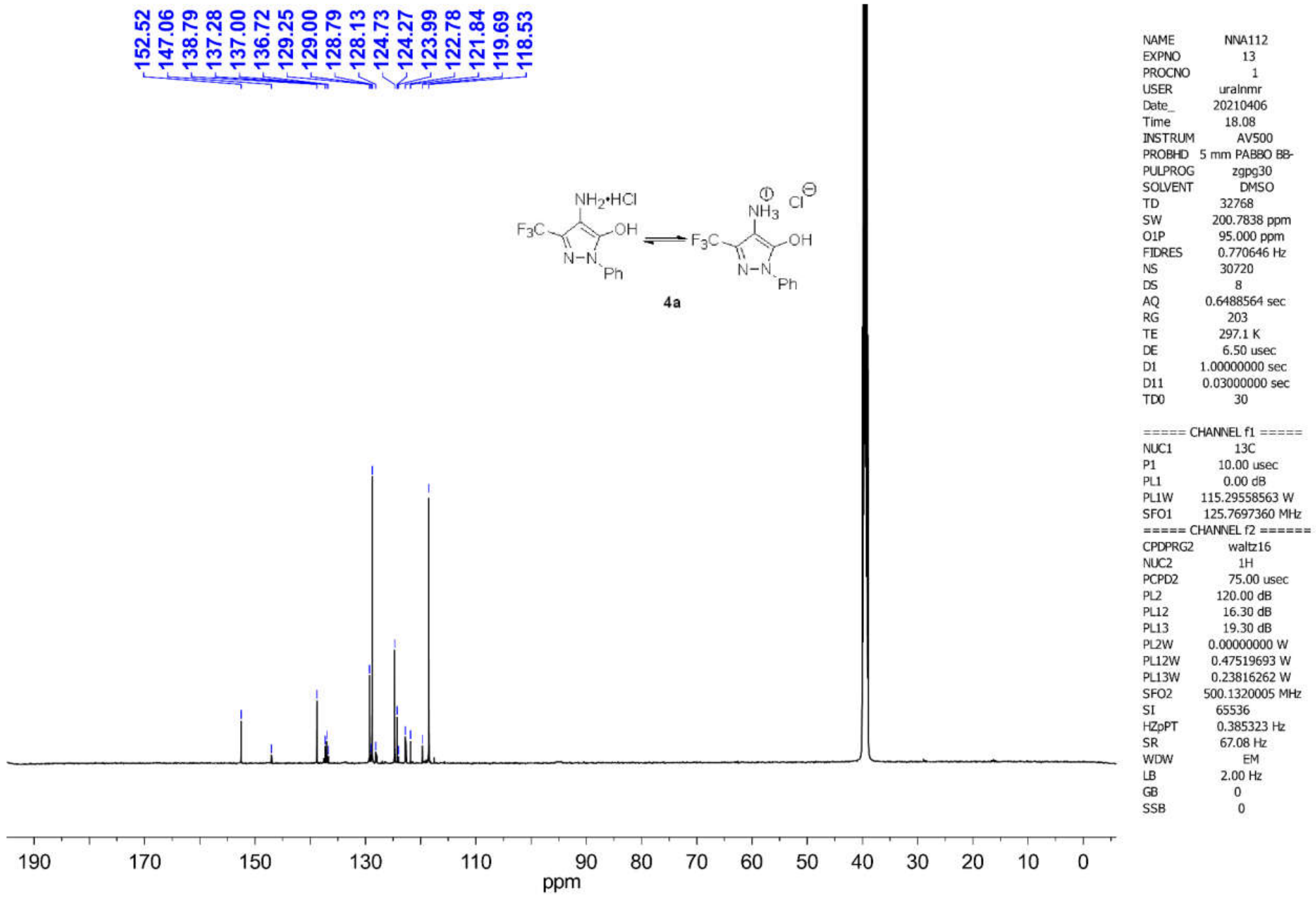

Figure S11. ^{19}F NMR spectrum of 4-amino-3-trifluoromethyl-1-phenyl-1H-pyrazol-5-ol hydrochloride (**4a**).

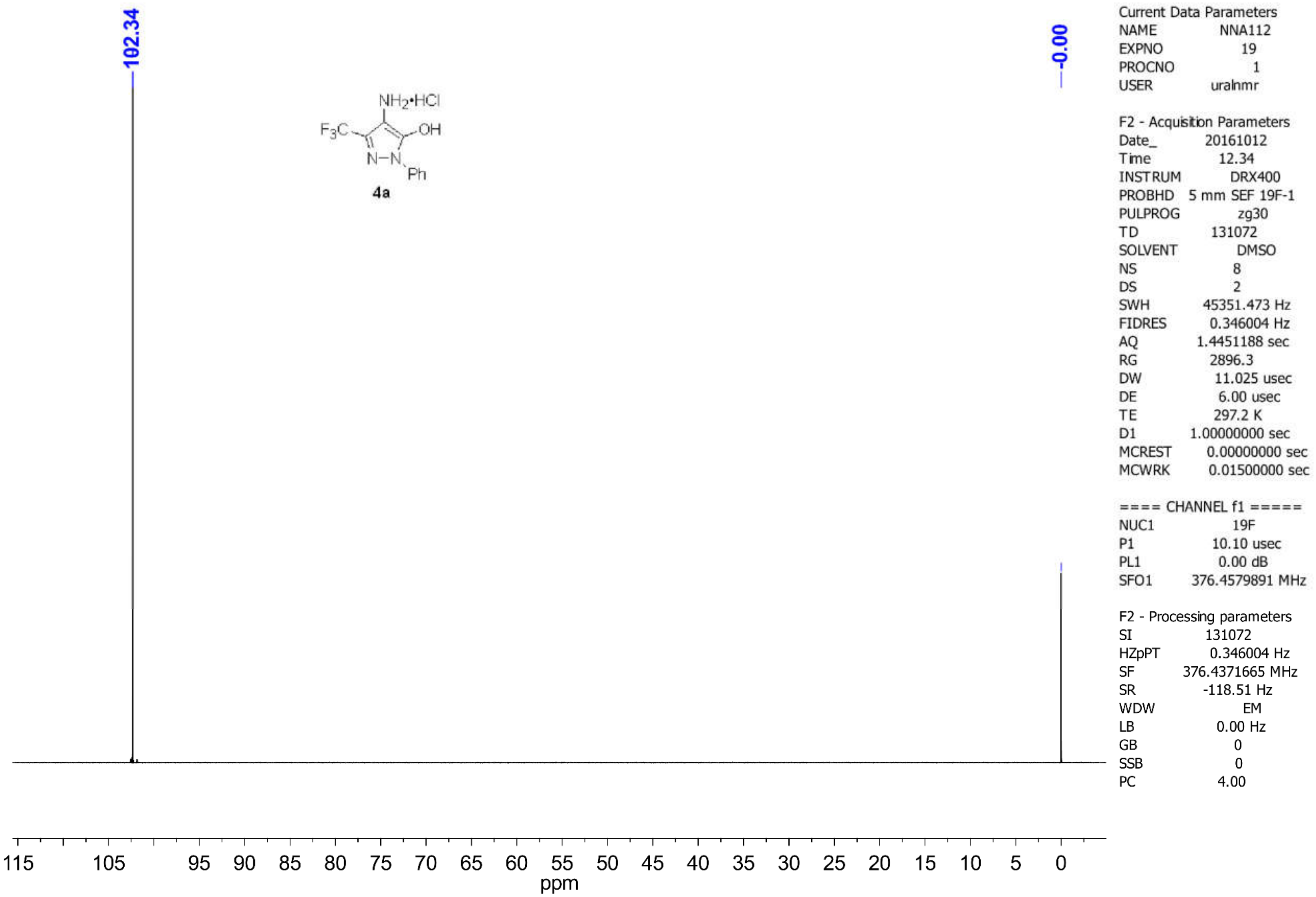

Figure S12. ^{1}H NMR spectrum of 4-amino-3-pentafluoroethyl-1-phenyl-1H-pyrazol-5-ol hydrate hydrochloride (**4b**).

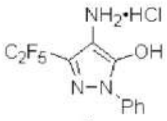

4b

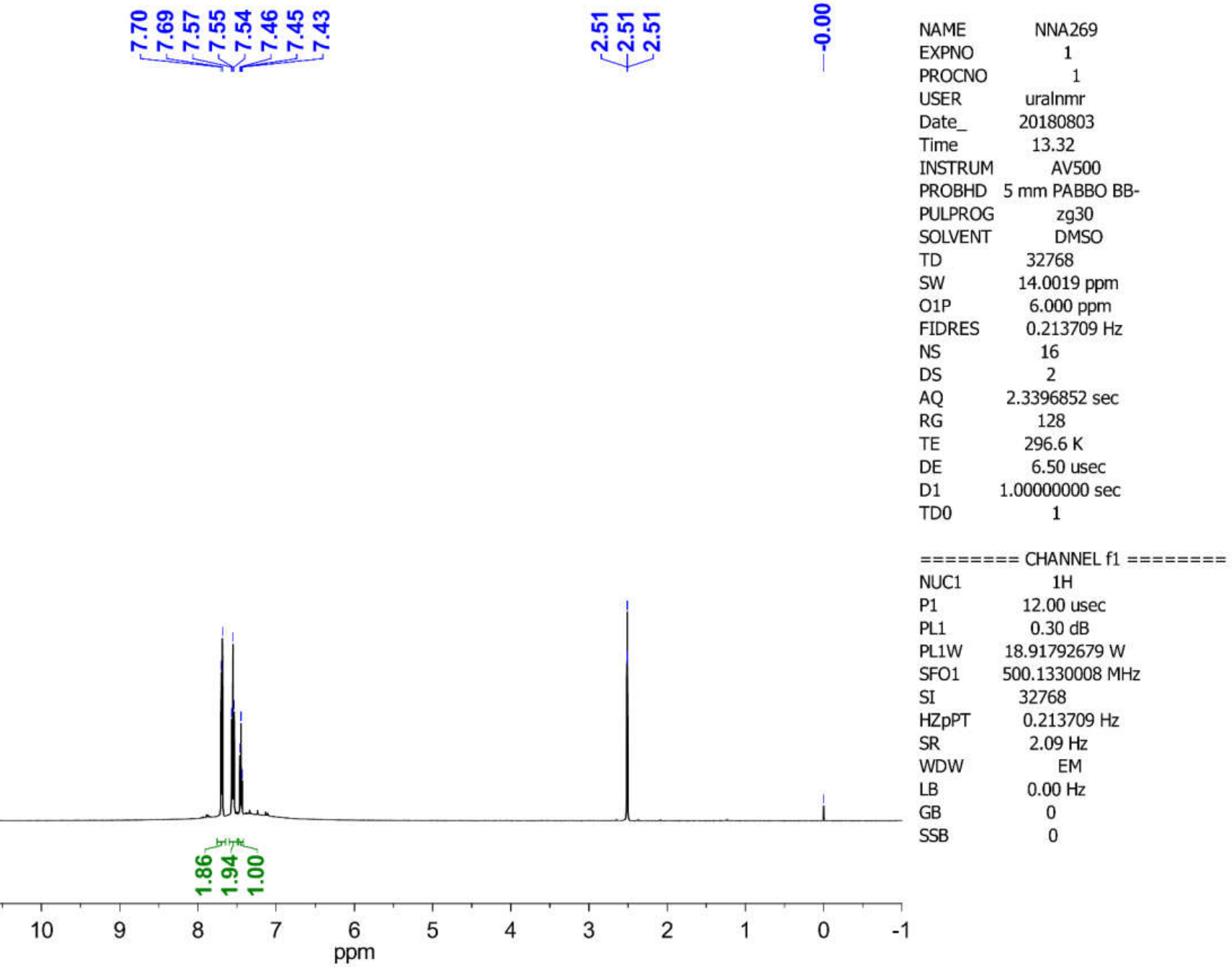

Figure S13. ^{1}H NMR spectrum of 4-amino-3-pentafluoroethyl-1-phenyl-1H-pyrazol-5-ol hydrate hydrochloride (**4b**) after standing overnight.

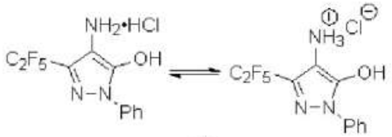

4b

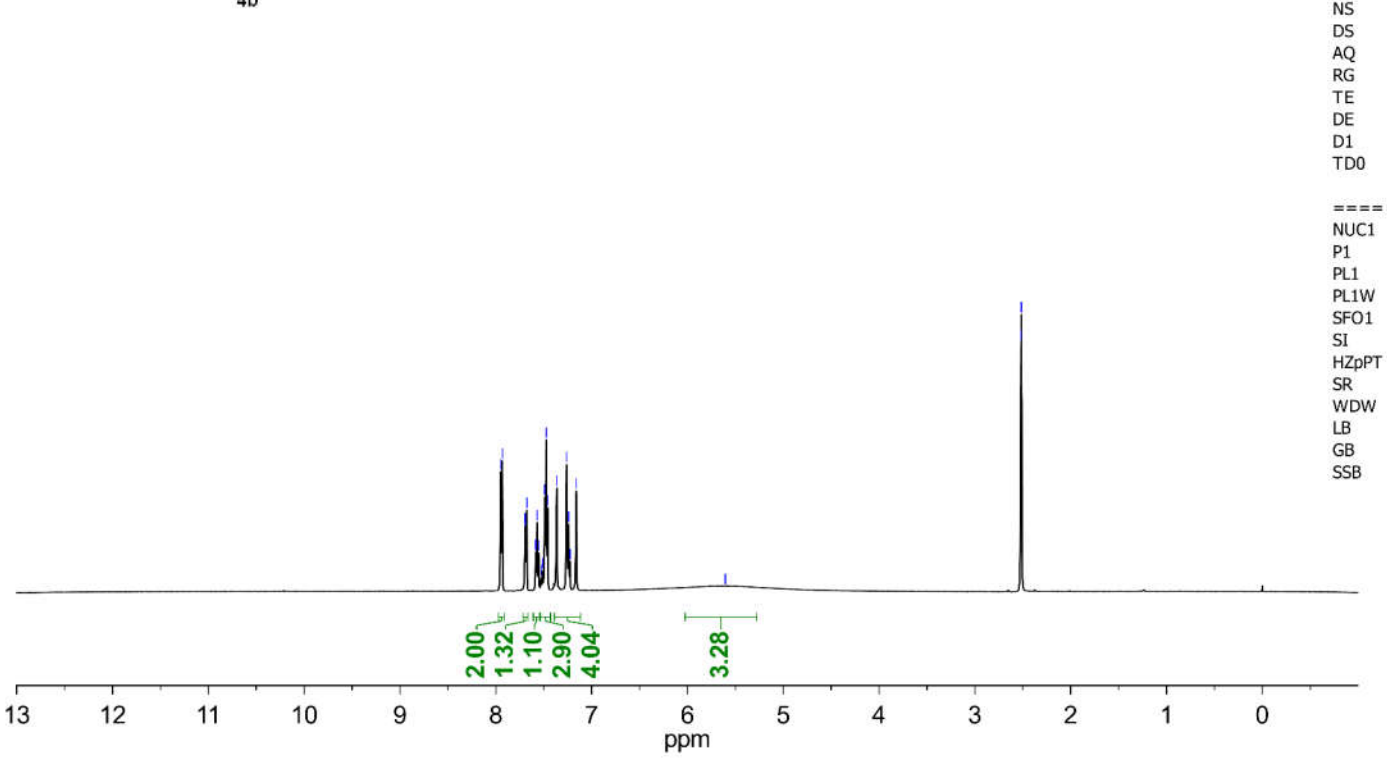

NAME	NNA269
EXPNO	3
PROCNO	1
USER	uralnmr
Date_	20180807
Time	12.39
INSTRUM	AV500
PROBHD	5 mm PABBO BB-
PULPROG	zg30
SOLVENT	DMSO
TD	32768
SW	14.0019 ppm
O1P	6.000 ppm
FIDRES	0.213709 Hz
NS	4
DS	2
AQ	2.3396852 sec
RG	114
TE	296.8 K
DE	6.50 usec
D1	1.00000000 sec
TD0	1

==== CHANNEL f1 =====

NUC1	1H
P1	12.00 usec
PL1	0.30 dB
PL1W	18.91792679 W
SFO1	500.1330008 MHz
SI	32768
HZpPT	0.213709 Hz
SR	-2.03 Hz
WDW	EM
LB	0.00 Hz
GB	0
SSB	0

Figure S14. ^{13}C NMR spectrum of 4-amino-3-pentafluoroethyl-1-phenyl-1H-pyrazol-5-ol hydrate hydrochloride (**4b**).

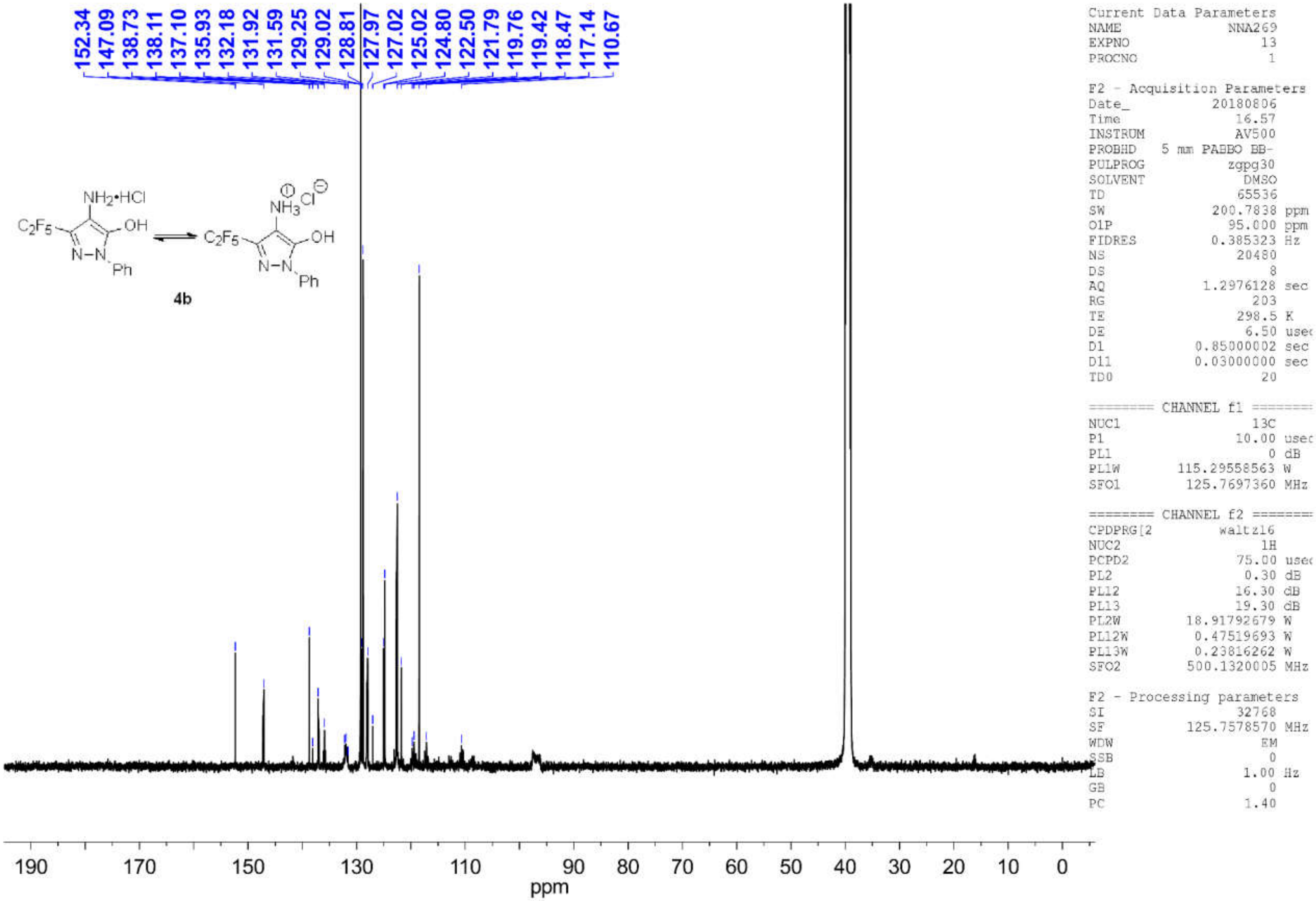

Figure S15. ^{19}F NMR spectrum of 4-amino-3-pentafluoroethyl-1-phenyl-1H-pyrazol-5-ol hydrate hydrochloride (**4b**).

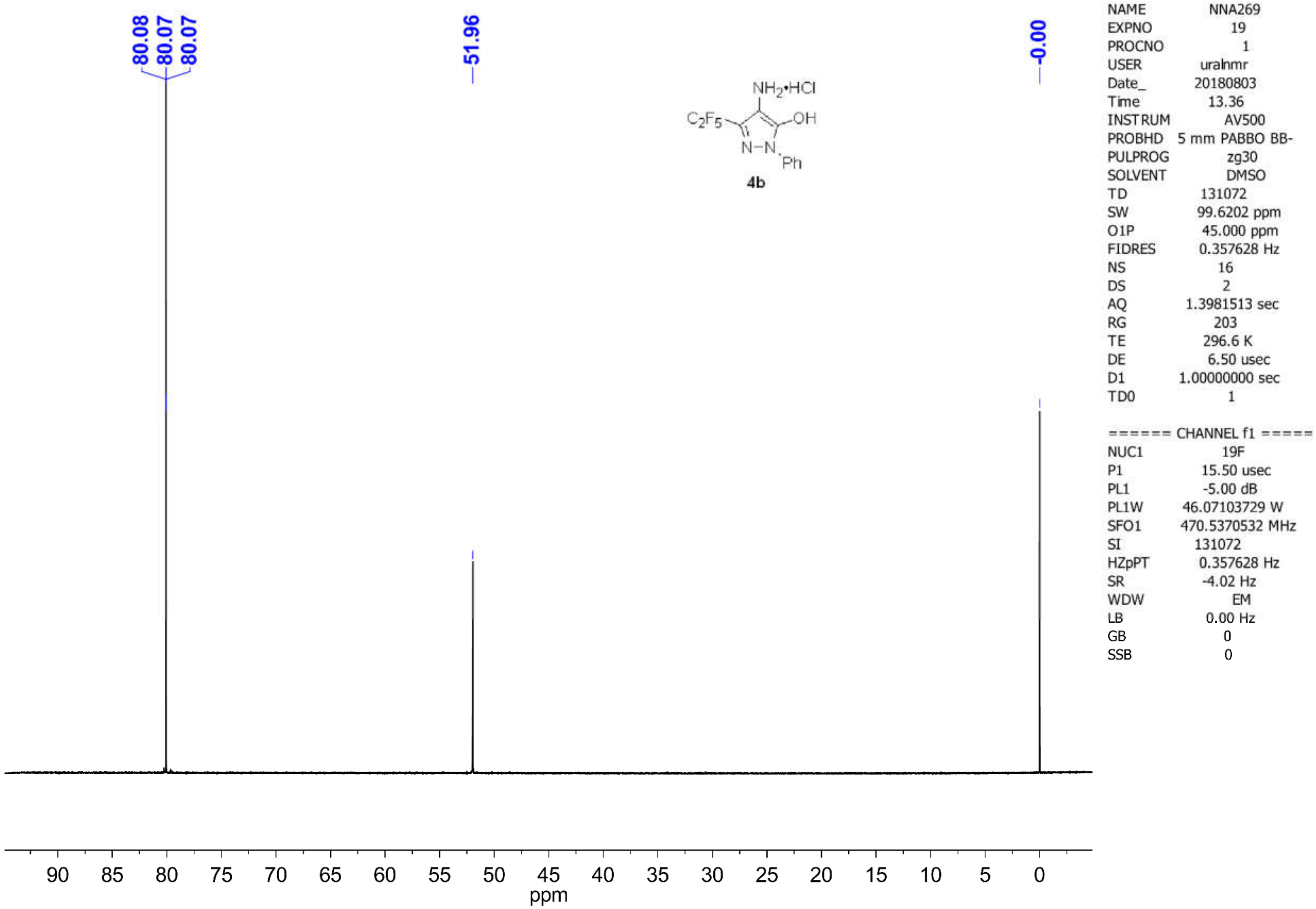

Figure S16. ^{1}H NMR spectrum of 5-hydroxy-1-phenyl-3-nonafluorobuthyl-1H-pyrazol-4-ammonium hydrochloride (**4c**).

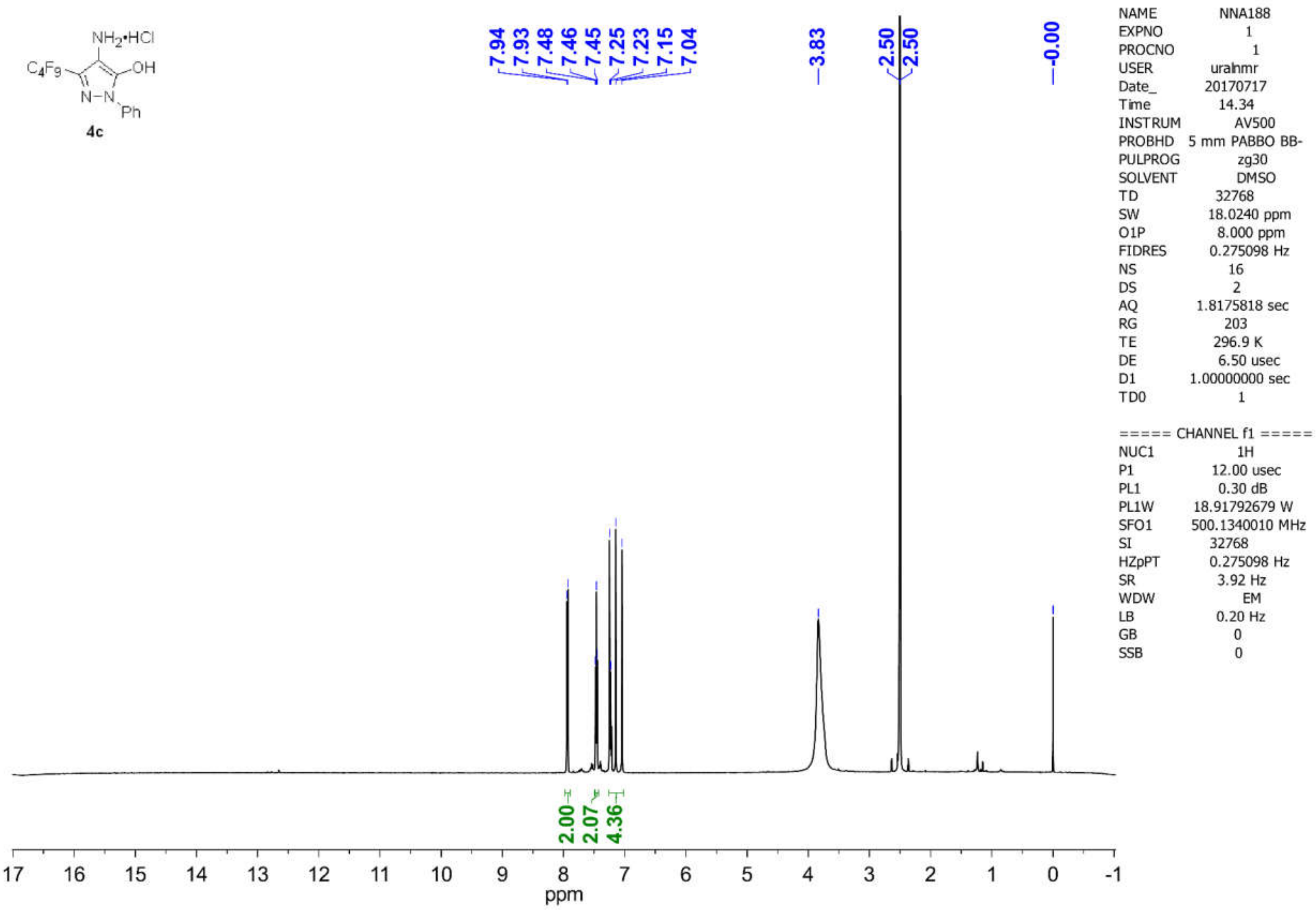

Figure S17. ^{19}F NMR spectrum of 5-hydroxy-1-phenyl-3-nonafluorobuthyl-1H-pyrazol-4-ammonium hydrochloride (**4c**).

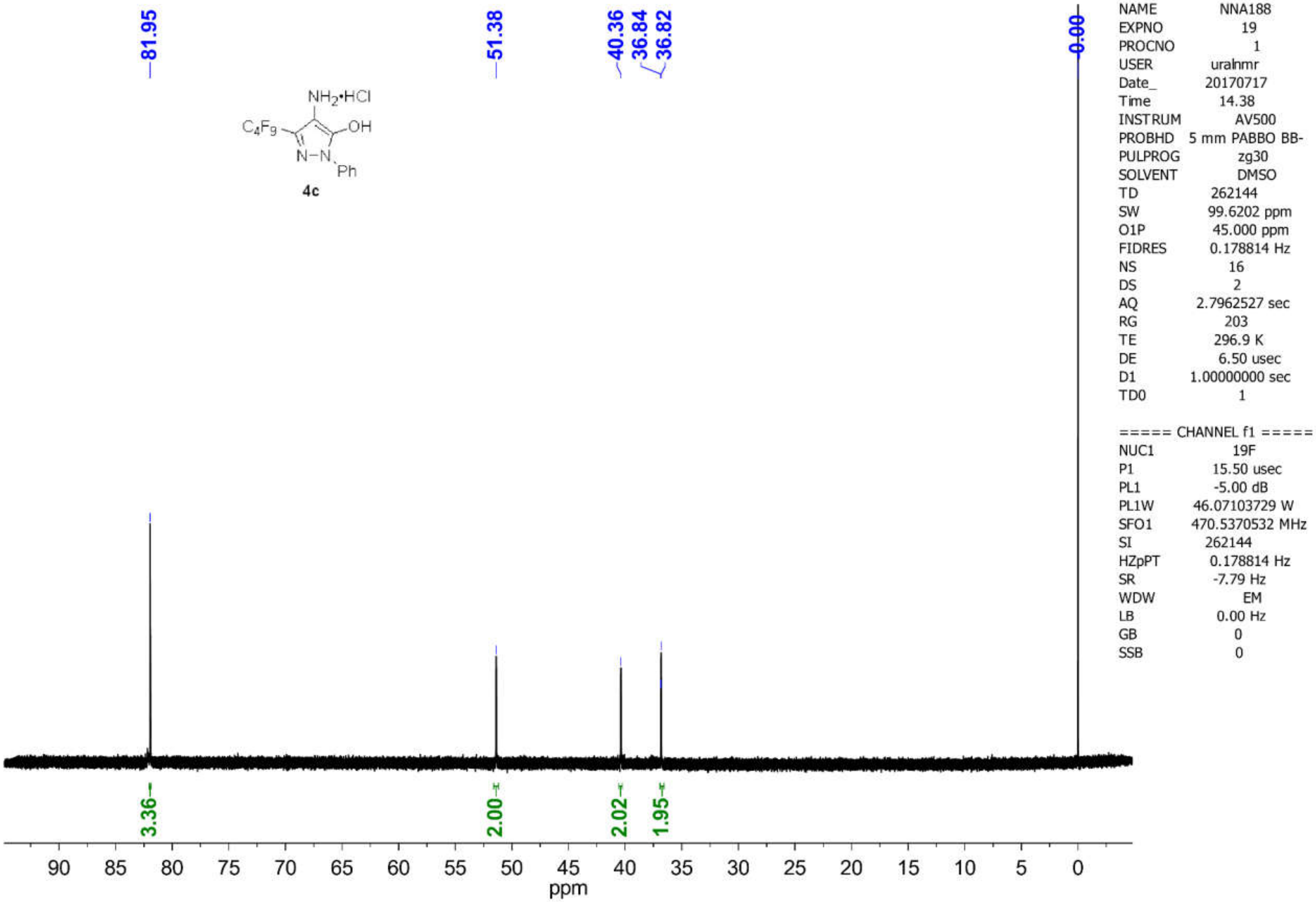

Figure S18. ^{1}H NMR spectrum of 4-amino-3-methyl-1-phenyl-1H-pyrazol-5-ol hydrochloride hydrate (**4d**).

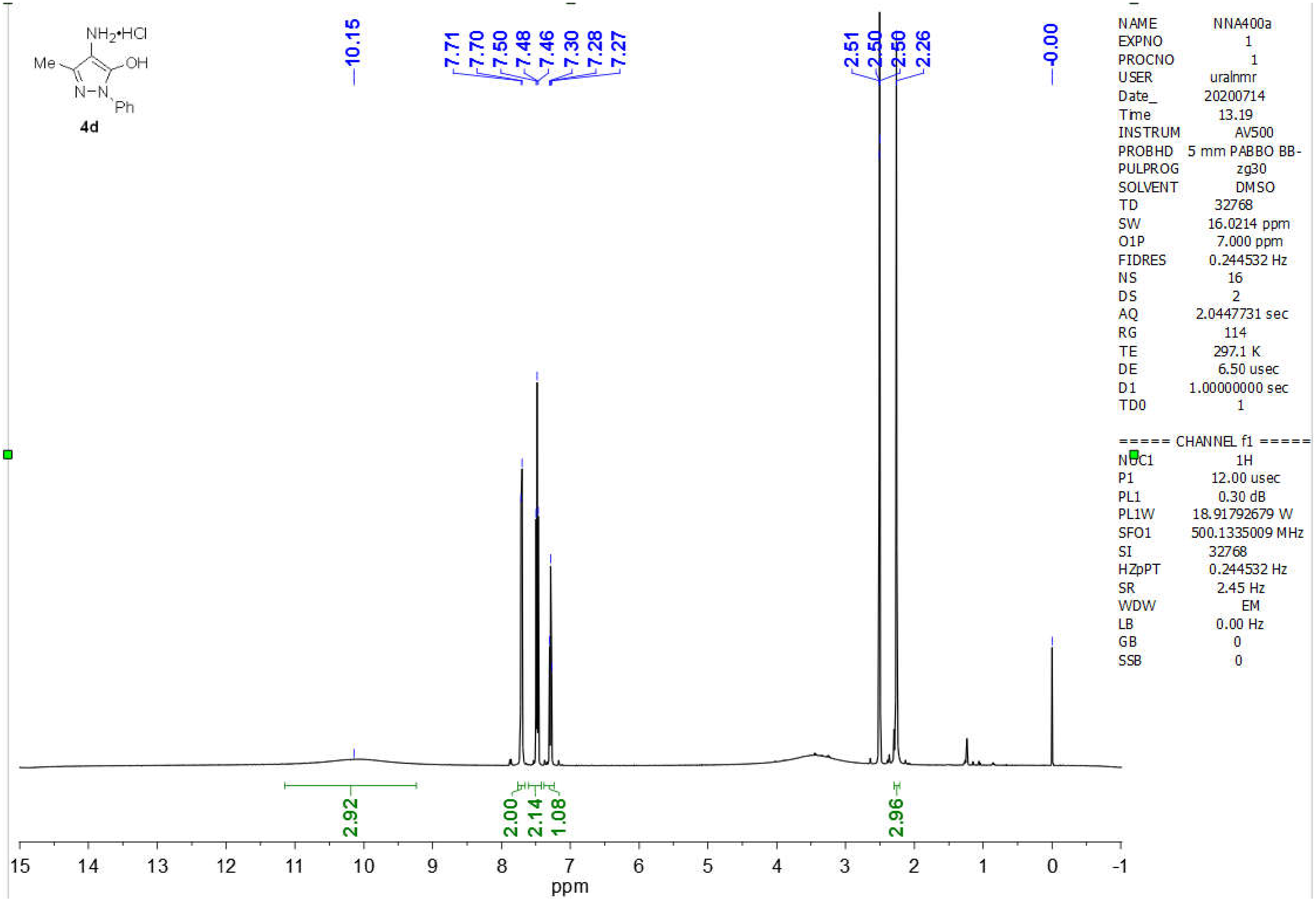

Figure S19. ^{13}C NMR spectrum of 4-amino-3-methyl-1-phenyl-1H-pyrazol-5-ol hydrochloride hydrate (**4d**).

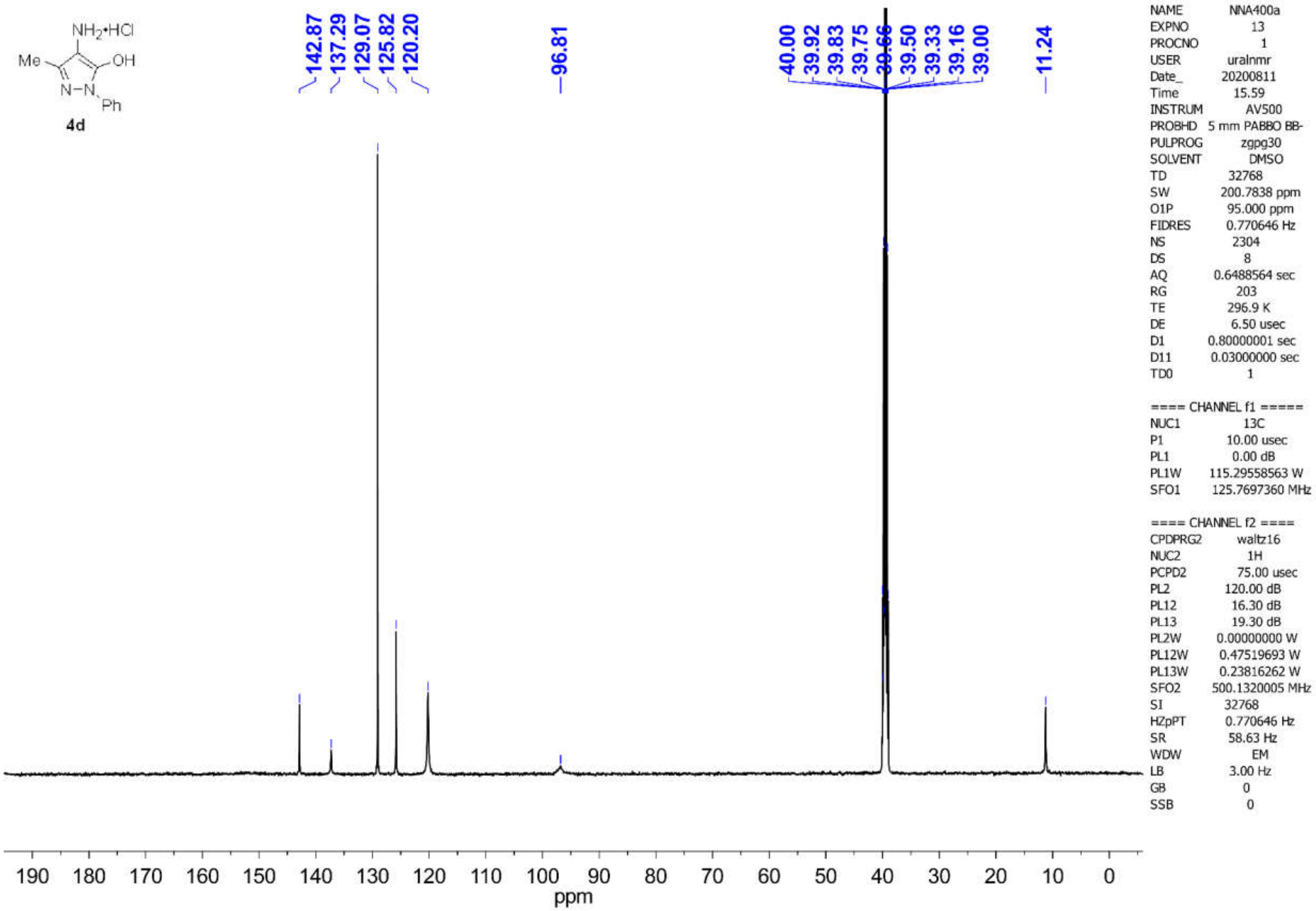

Figure S20. ^{1}H NMR spectrum of 4-amino-3-trifluoromethyl-1H-pyrazol-5-ol hydrochloride (**4e**).

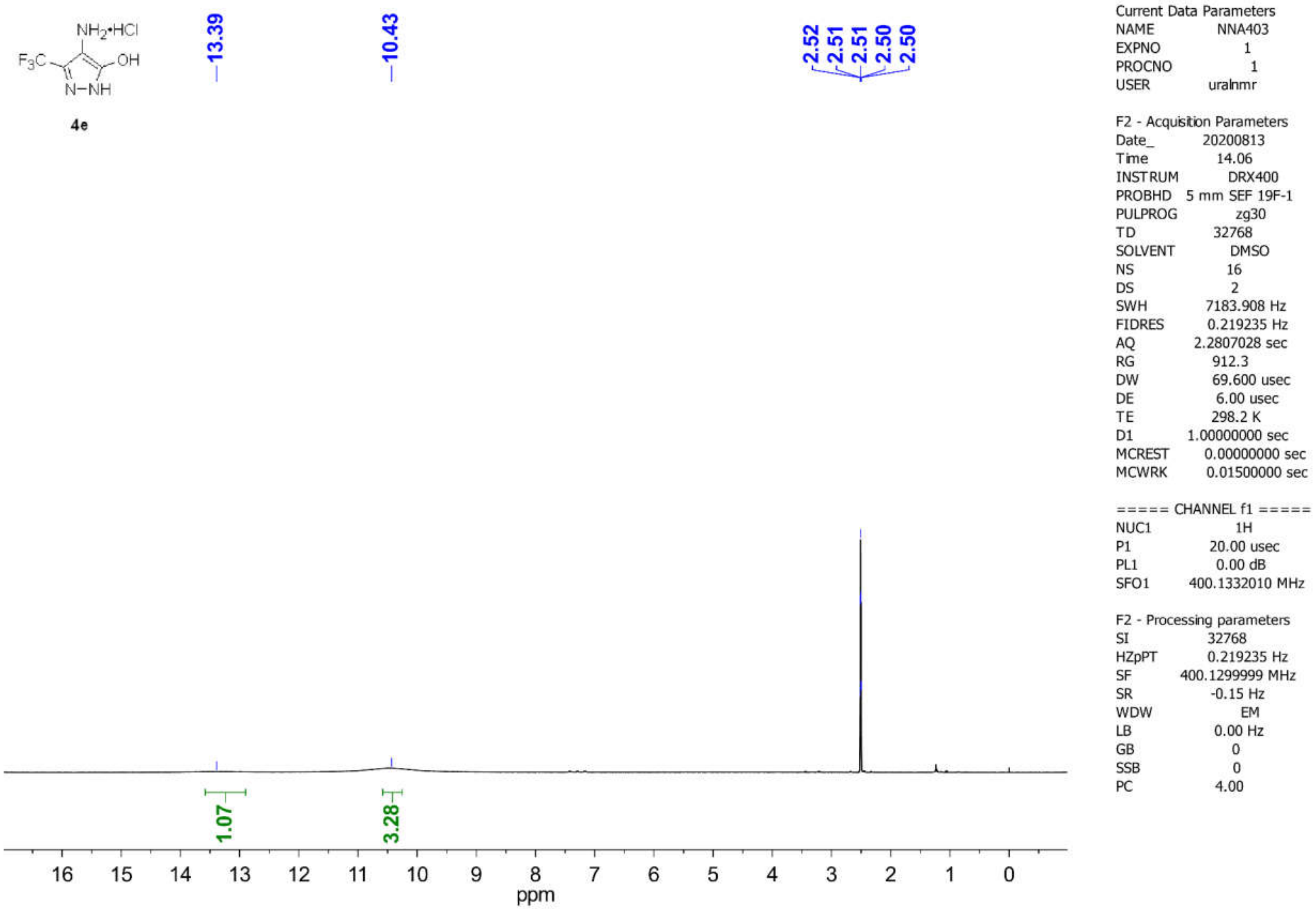

Figure S21. ^{13}C NMR spectrum of 4-amino-3-trifluoromethyl-1H-pyrazol-5-ol hydrochloride (**4e**).

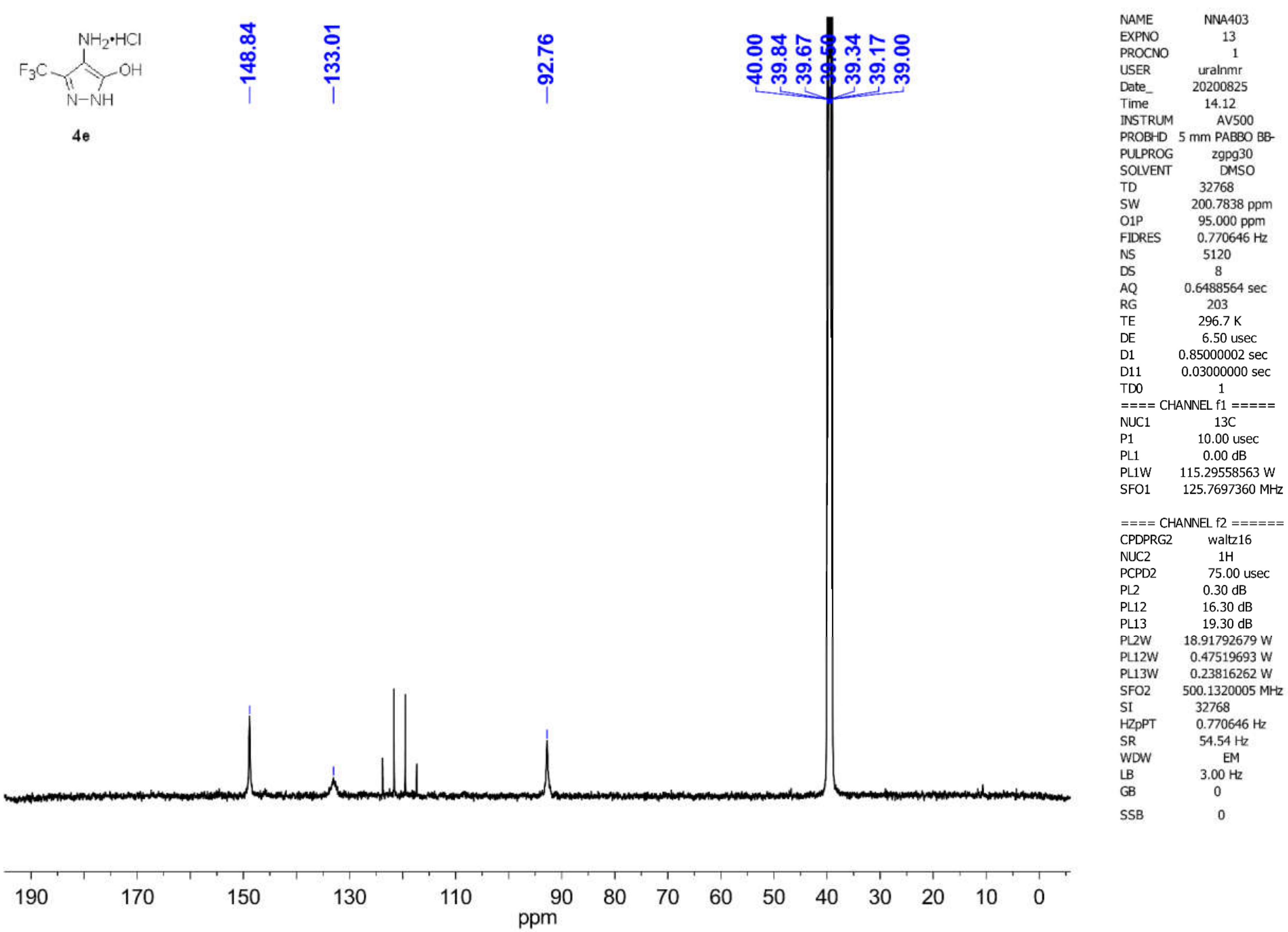

Figure S22. ^{19}F NMR spectrum of 4-amino-3-trifluoromethyl-1H-pyrazol-5-ol hydrochloride (**4e**).

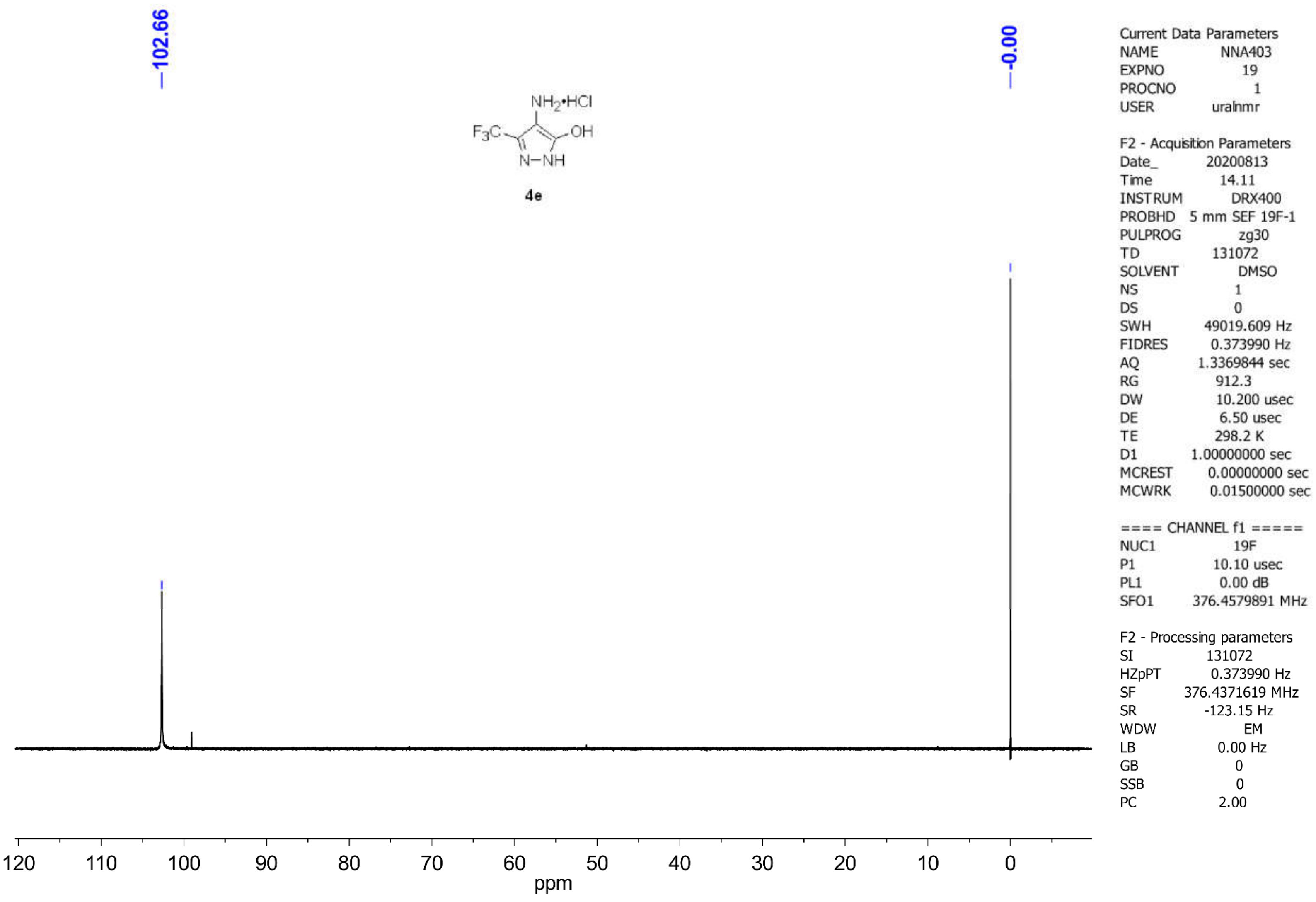

Figure S23. ^{1}H NMR spectrum of 4-amino-3-(1,1,2,2-tetrafluoroethyl)-1H-pyrazol-5-ol hydrate hydrochloride (**4f**).

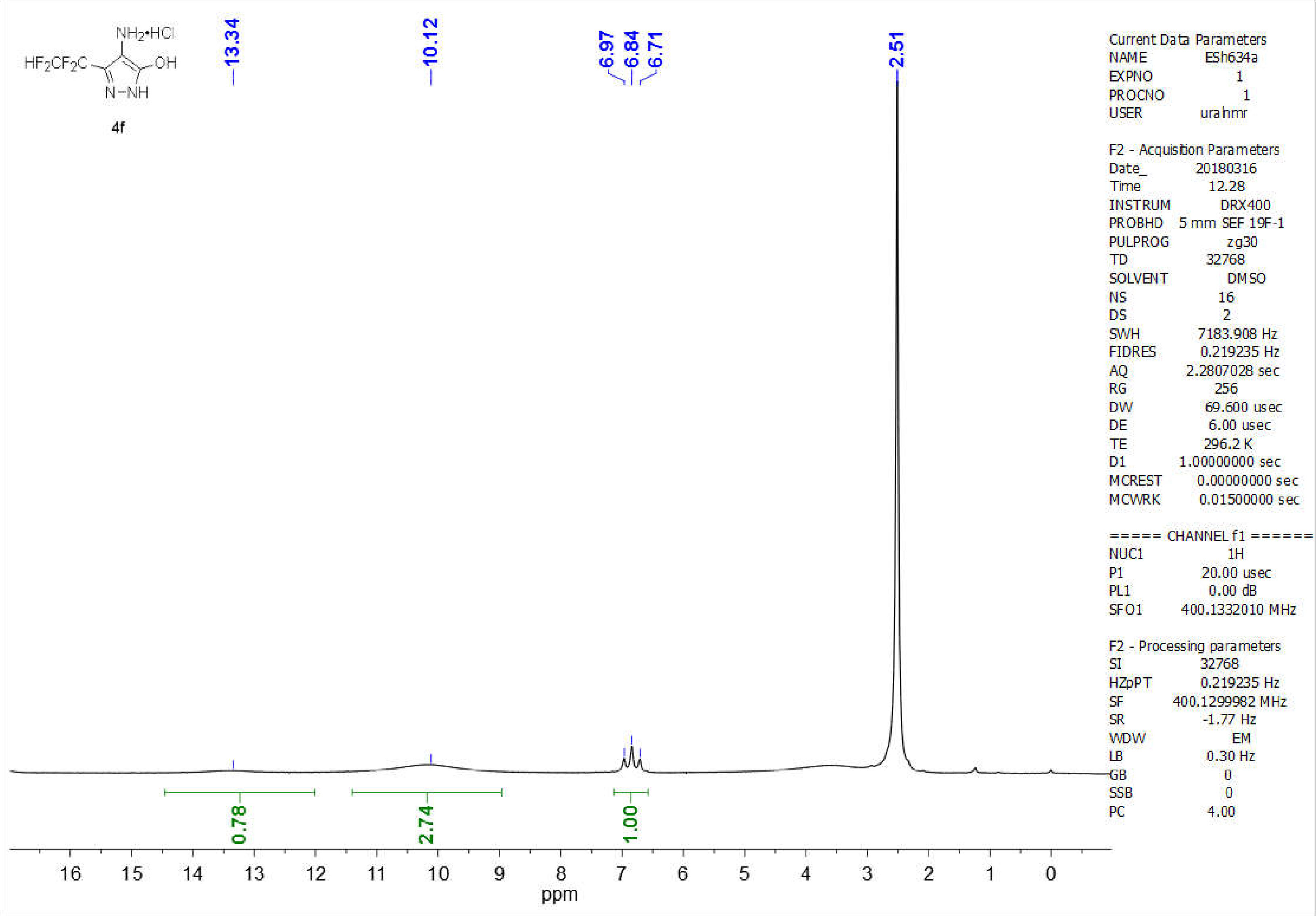

Figure S24. ^{19}F NMR spectrum of 4-amino-3-(1,1,2,2-tetrafluoroethyl)-1H-pyrazol-5-ol hydrate hydrochloride (**4f**).

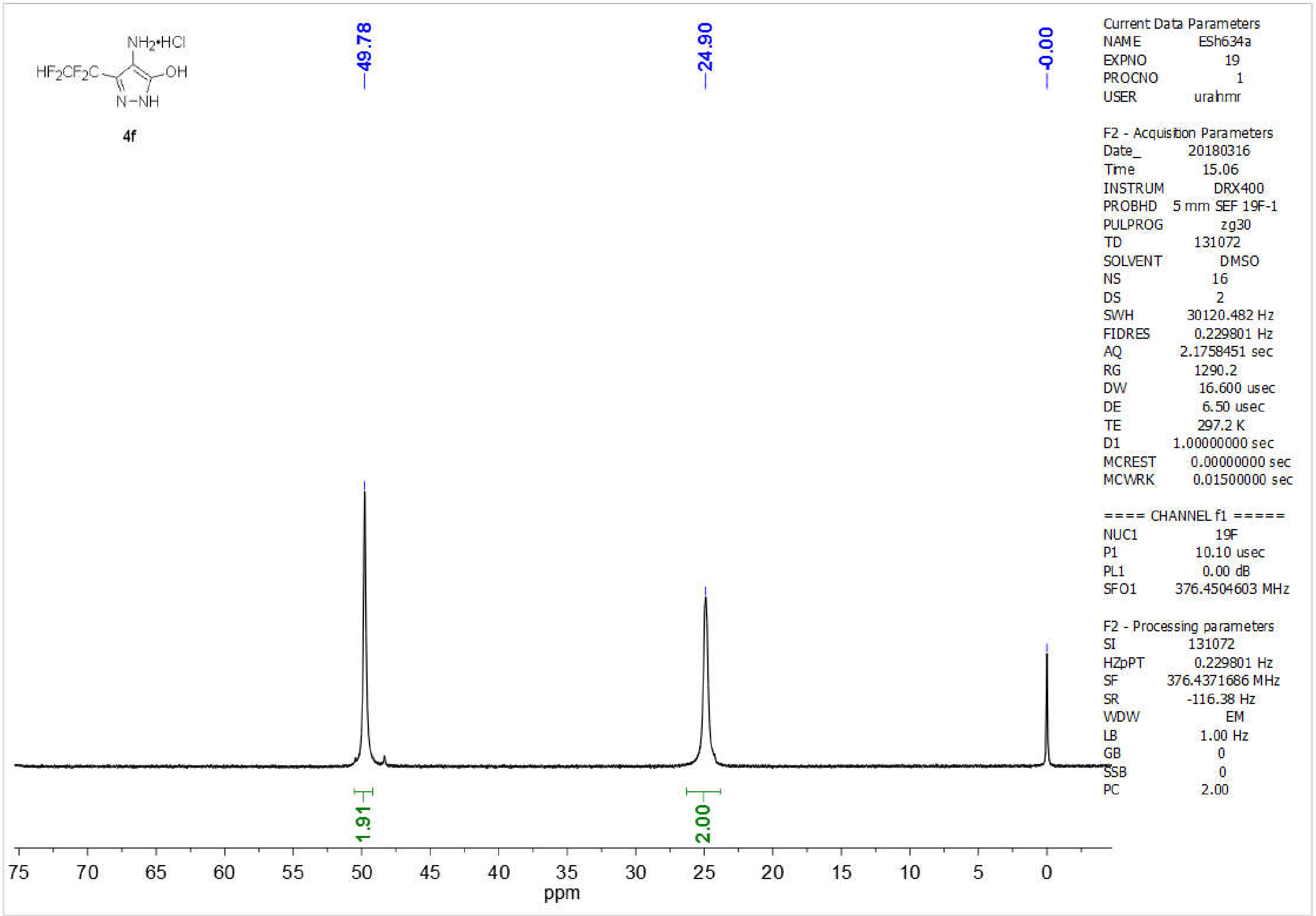

Figure S25. ^{1}H NMR spectrum of 4-amino-3-(pentafluoroethyl)-1H-pyrazol-5-ol hydrate hydrochloride (**4g**).

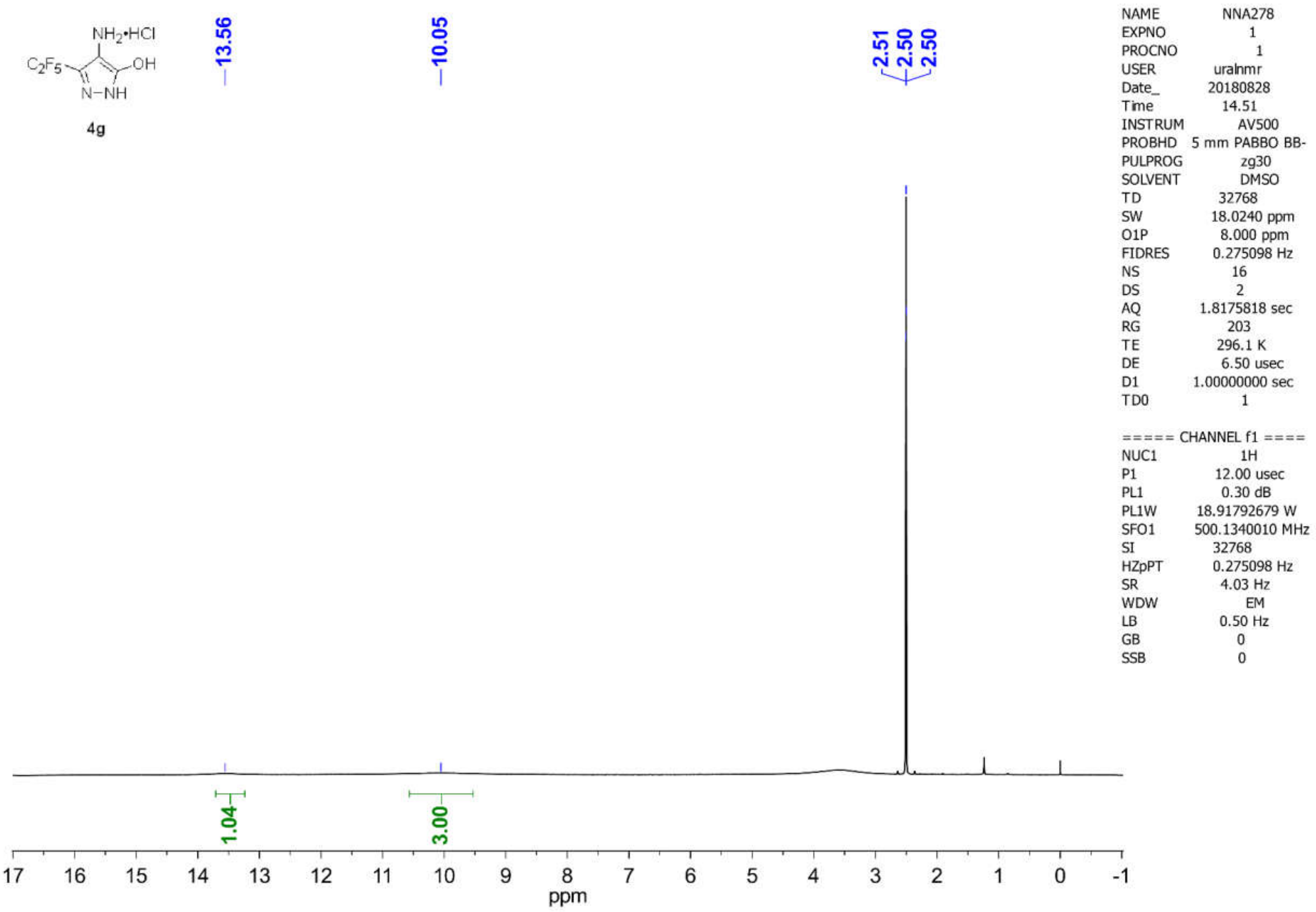

Figure S26. ^{19}F NMR spectrum of 4-amino-3-(pentafluoroethyl)-1H-pyrazol-5-ol hydrate hydrochloride (**4g**).

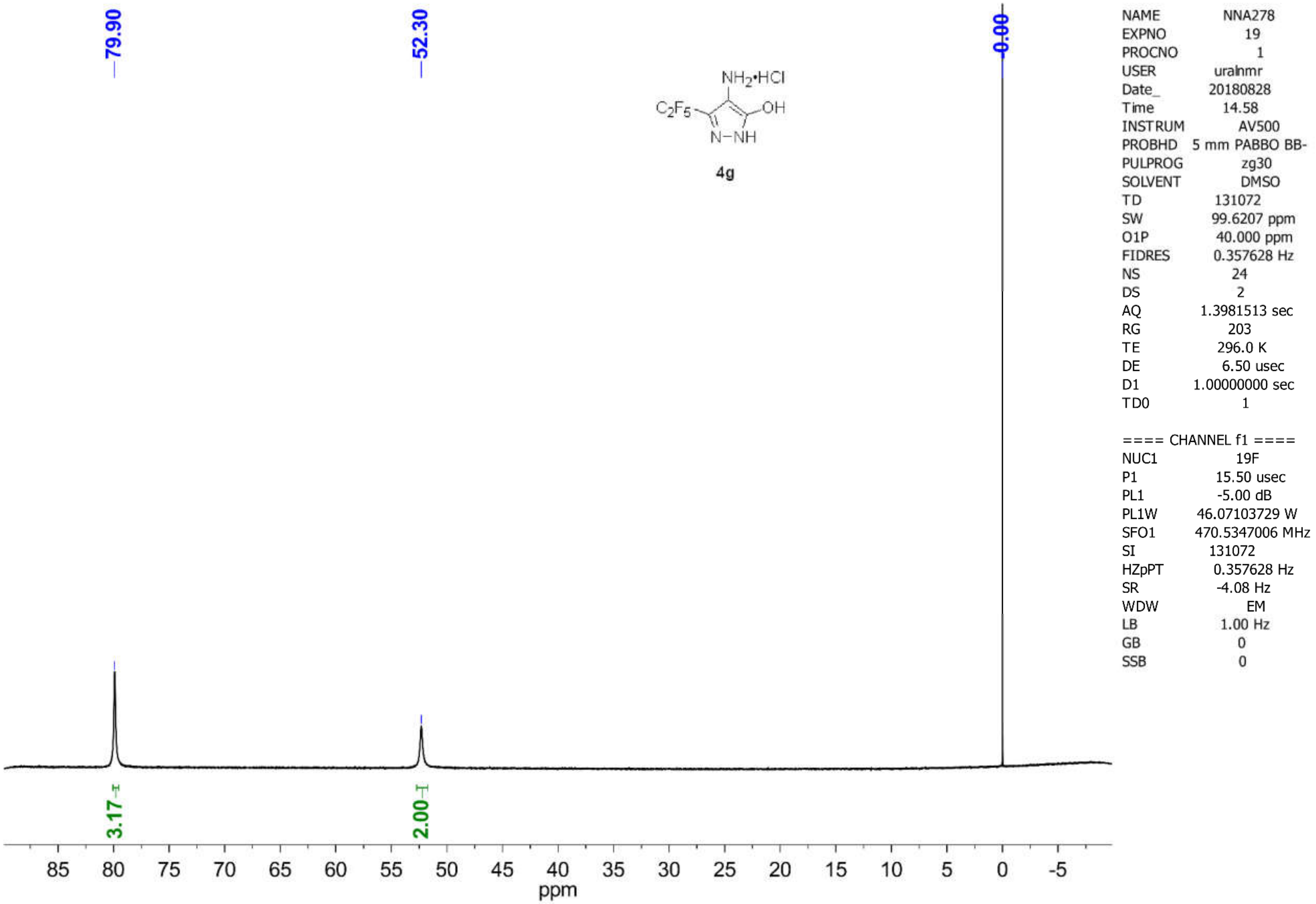

Figure S27. ^{1}H NMR spectrum of 4-amino-3-(heptafluoropropyl)-1H-pyrazol-5-ol hydrate hydrochloride (**4h**).

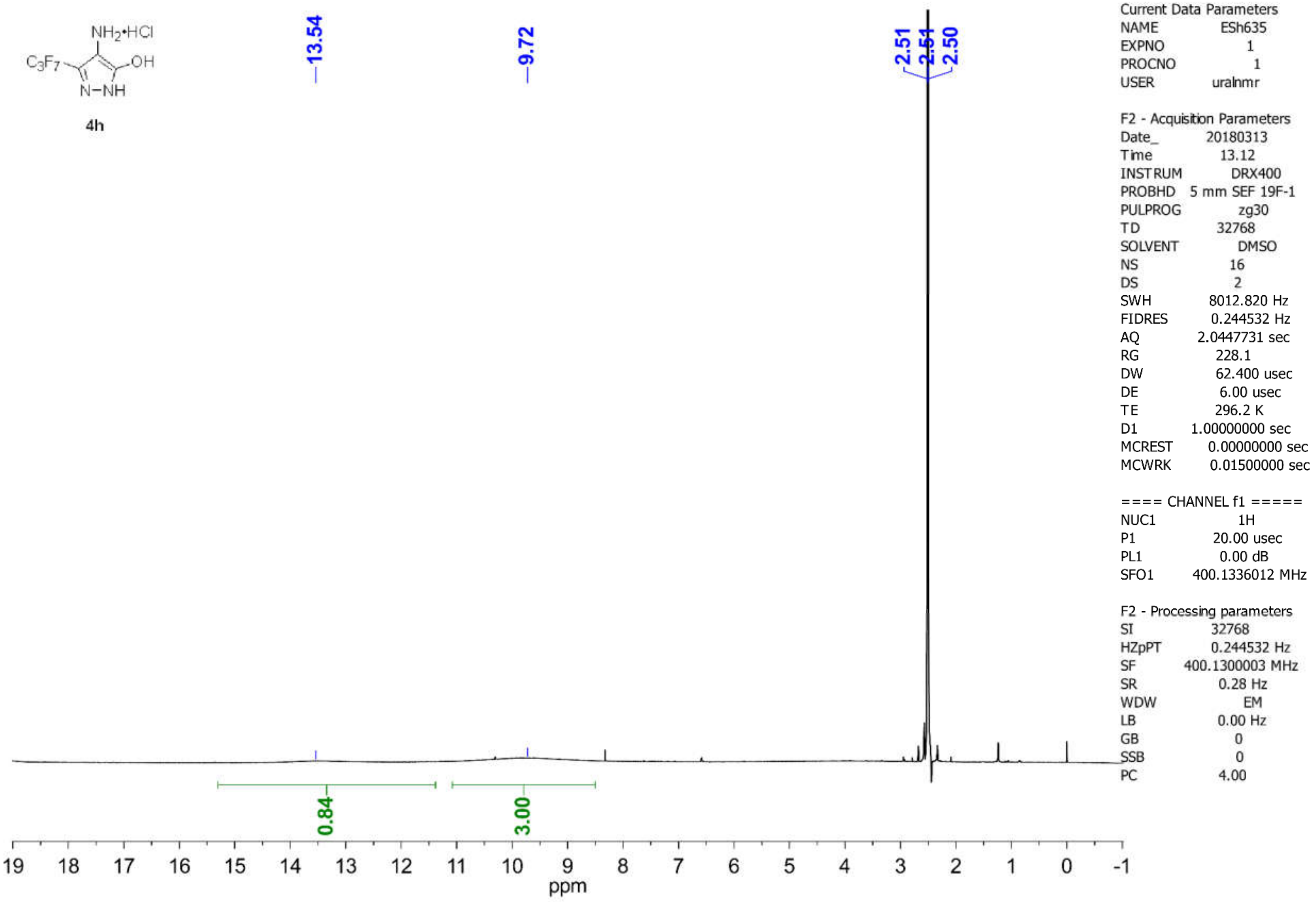

Figure S28. ^{19}F NMR spectrum of 4-amino-3-(heptafluoropropyl)-1H-pyrazol-5-ol hydrate hydrochloride (**4h**).

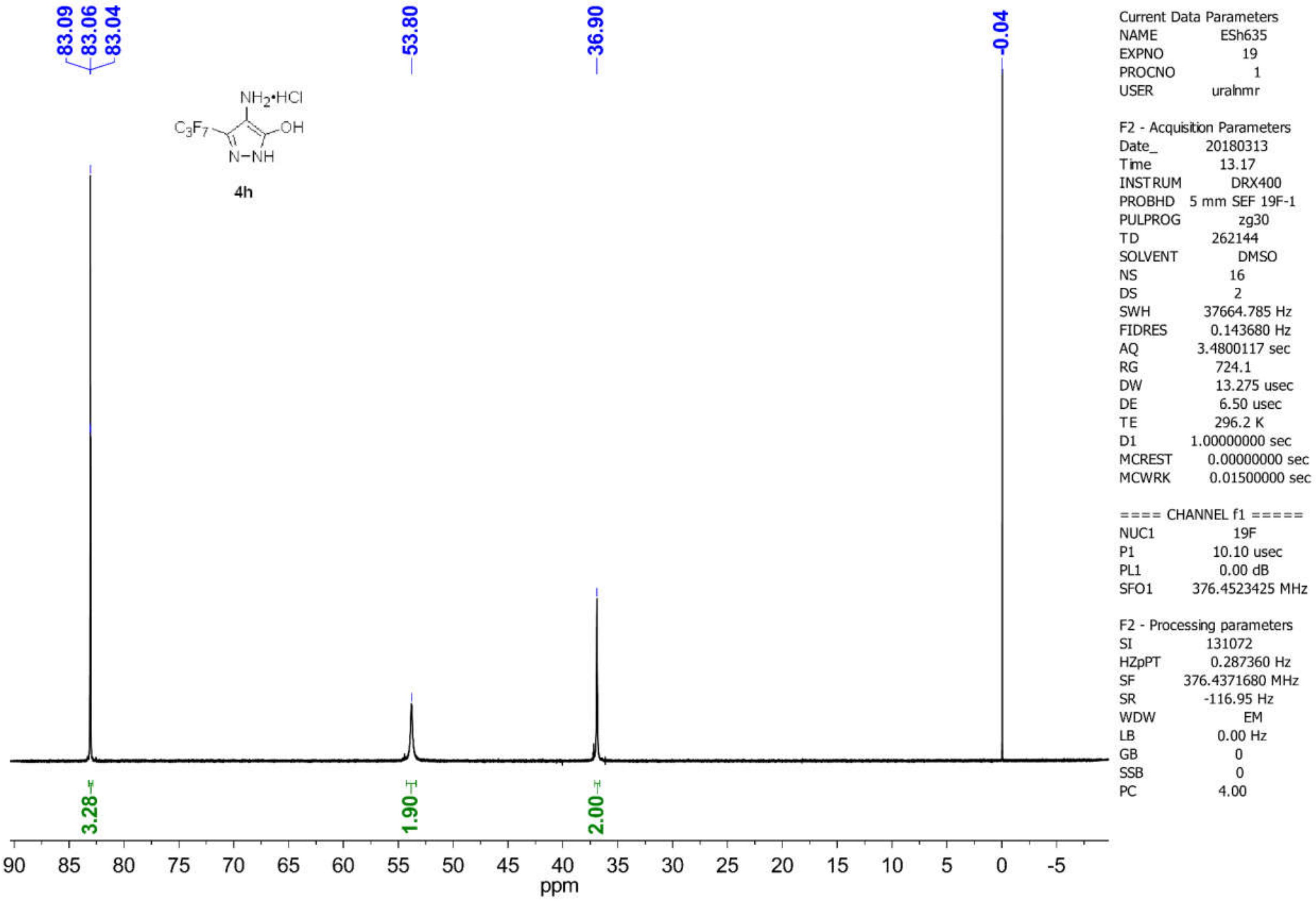

Figure S29. ^{1}H NMR spectrum of 4-amino-3-(nonafluorobutyl)-1H-pyrazol-5-ol hydrate hydrochloride (**4i**).

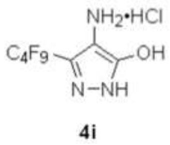

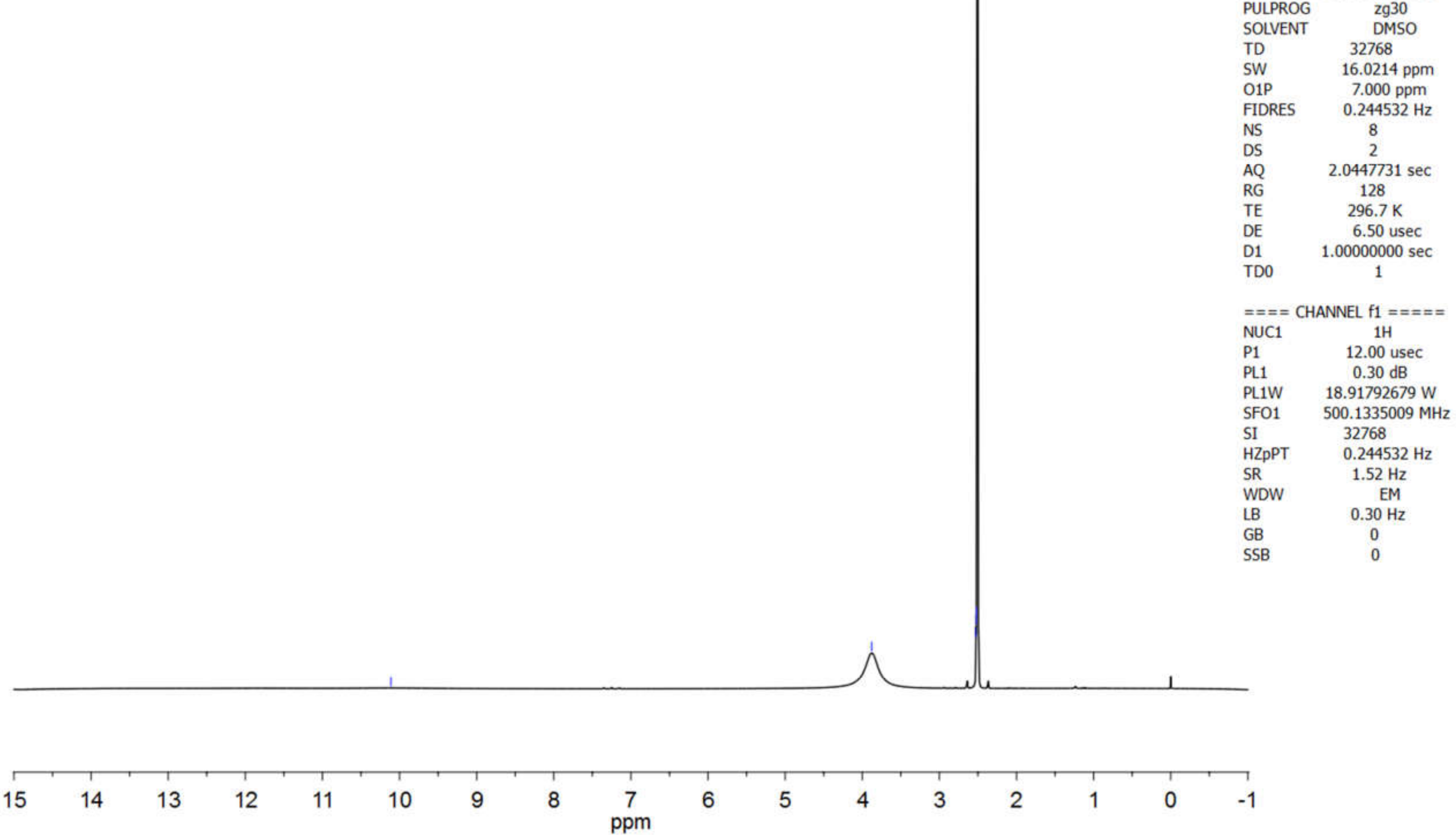

Figure S30. ^{19}F NMR spectrum of 4-amino-3-(nonafluorobutyl)-1H-pyrazol-5-ol hydrate hydrochloride (**4i**).

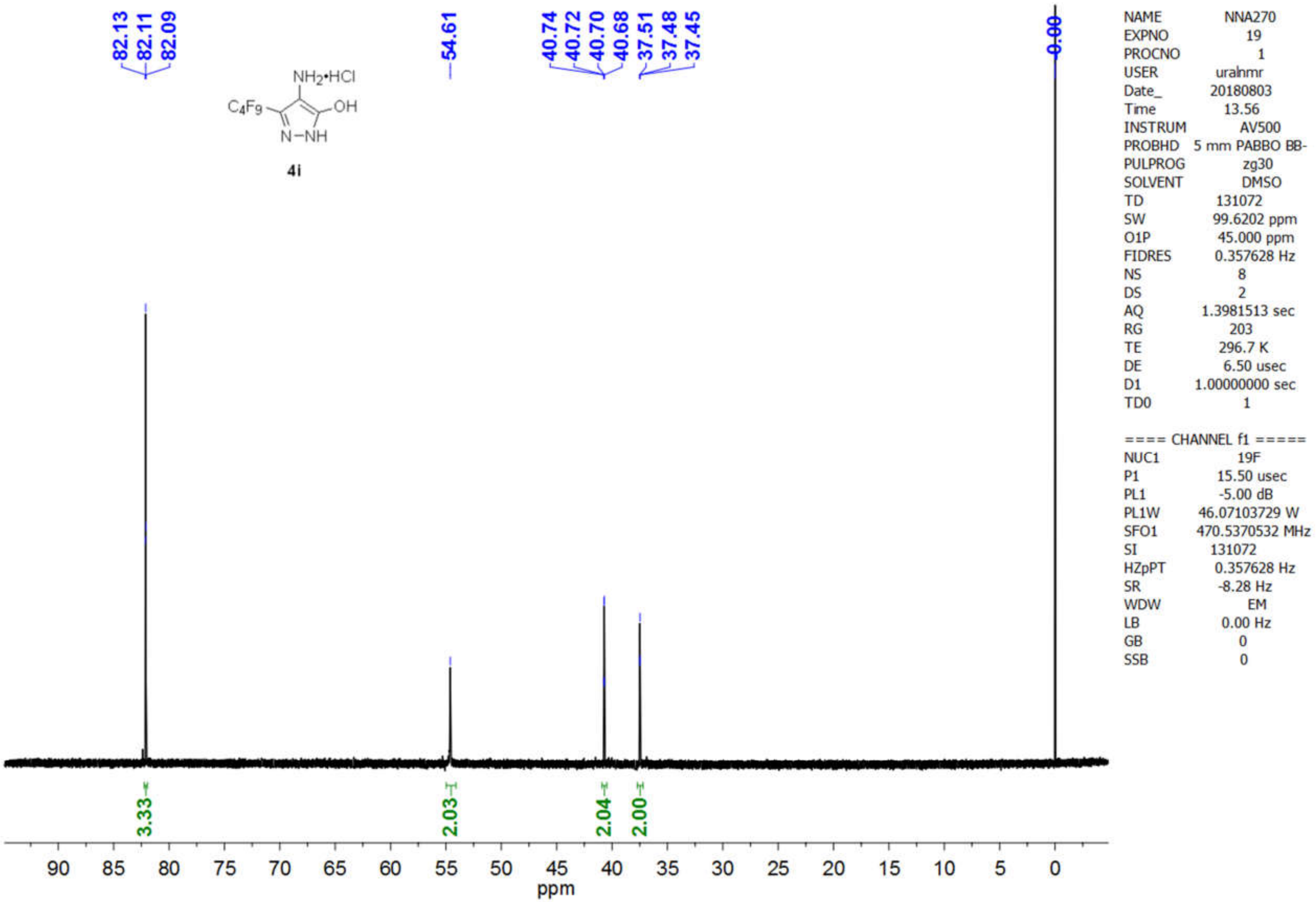

Figure S31. ^{1}H NMR spectrum of 4-amino-3-methyl-1H-pyrazol-5-ol hydrochloride (**4j**).

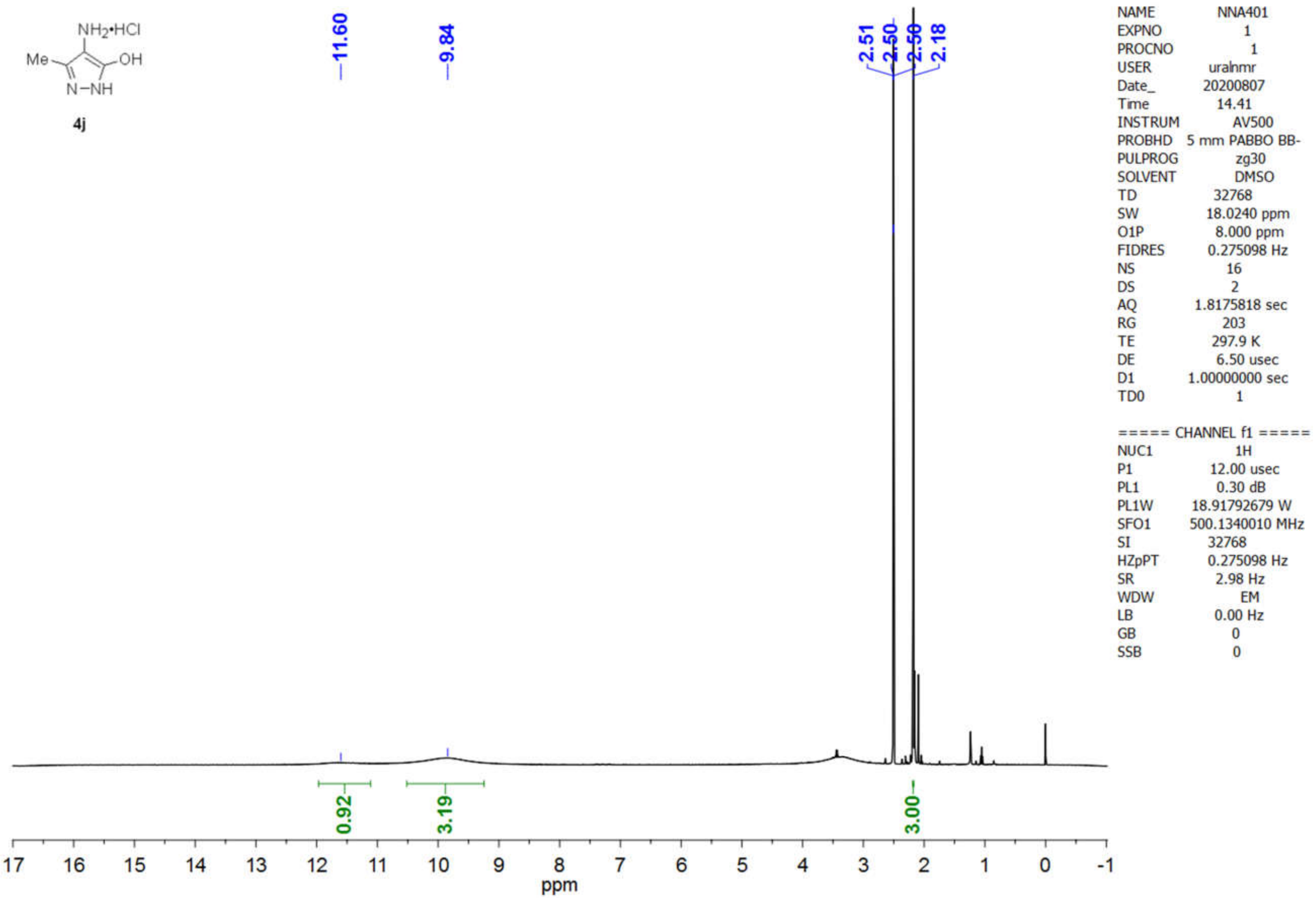

Figure S32. ^{13}C NMR spectrum of 4-amino-3-methyl-1H-pyrazol-5-ol hydrochloride (**4j**).

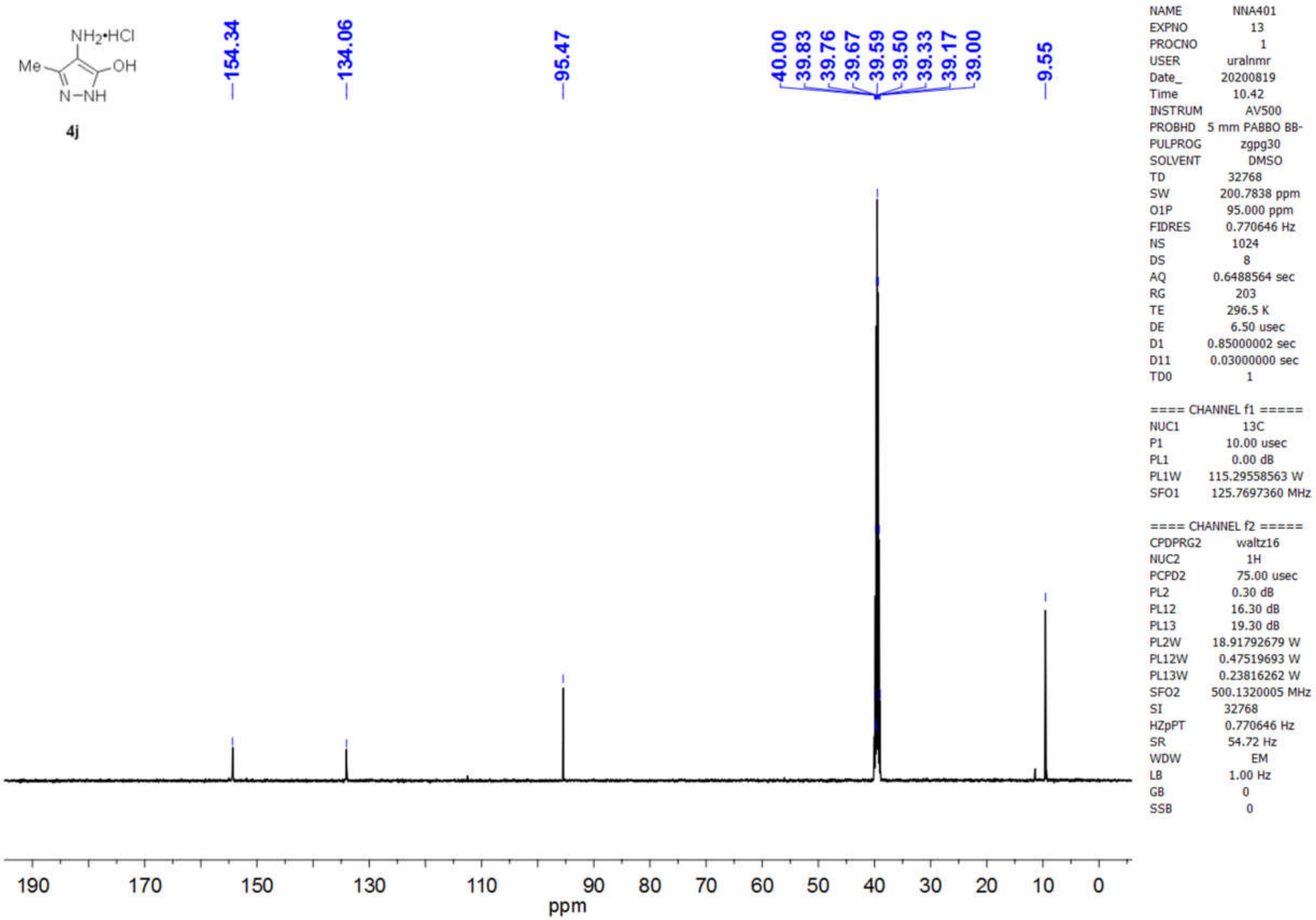

Supplement: Supplementary file 1 [file molecules-27-07722-s001.zip › molecules-2015150-supplementary.pdf]
